# Evaluating the effect of disturbed ensemble distributions on SCFG based statistical sampling of RNA secondary structures

**DOI:** 10.1186/1471-2105-13-159

**Published:** 2012-07-09

**Authors:** Anika Scheid, Markus E Nebel

**Affiliations:** 1 Department of Computer Science, University of Kaiserslautern, P.O. Box 3049, D-67653 Kaiserslautern, Germany

## Abstract

**Background:**

Over the past years, statistical and Bayesian approaches have become increasingly appreciated to address the long-standing problem of computational RNA structure prediction. Recently, a novel probabilistic method for the prediction of RNA secondary structures from a single sequence has been studied which is based on generating statistically representative and reproducible samples of the entire ensemble of feasible structures for a particular input sequence. This method samples the possible foldings from a distribution implied by a sophisticated (traditional or length-dependent) stochastic context-free grammar (SCFG) that mirrors the standard thermodynamic model applied in modern physics-based prediction algorithms. Specifically, that grammar represents an exact probabilistic counterpart to the energy model underlying the Sfold software, which employs a sampling extension of the partition function (PF) approach to produce statistically representative subsets of the Boltzmann-weighted ensemble. Although both sampling approaches have the same worst-case time and space complexities, it has been indicated that they differ in performance (both with respect to prediction accuracy and quality of generated samples), where neither of these two competing approaches generally outperforms the other.

**Results:**

In this work, we will consider the SCFG based approach in order to perform an analysis on how the quality of generated sample sets and the corresponding prediction accuracy changes when different degrees of disturbances are incorporated into the needed sampling probabilities. This is motivated by the fact that if the results prove to be resistant to large errors on the distinct sampling probabilities (compared to the exact ones), then it will be an indication that these probabilities do not need to be computed exactly, but it may be sufficient and more efficient to approximate them. Thus, it might then be possible to decrease the worst-case time requirements of such an SCFG based sampling method without significant accuracy losses. If, on the other hand, the quality of sampled structures can be observed to strongly react to slight disturbances, there is little hope for improving the complexity by heuristic procedures. We hence provide a reliable test for the hypothesis that a heuristic method could be implemented to improve the time scaling of RNA secondary structure prediction in the worst-case – without sacrificing much of the accuracy of the results.

**Conclusions:**

Our experiments indicate that absolute errors generally lead to the generation of useless sample sets, whereas relative errors seem to have only small negative impact on both the predictive accuracy and the overall quality of resulting structure samples. Based on these observations, we present some useful ideas for developing a time-reduced sampling method guaranteeing an acceptable predictive accuracy. We also discuss some inherent drawbacks that arise in the context of approximation. The key results of this paper are crucial for the design of an efficient and competitive heuristic prediction method based on the increasingly accepted and attractive statistical sampling approach. This has indeed been indicated by the construction of prototype algorithms.

## Background

In computational structural biology, a well-established probabilistic methodology towards single sequence RNA secondary structure prediction is based on modeling secondary structures by *stochastic context-free grammars (SCFGs)*. In a sense, SCFGs can be seen as a generalization of *hidden Markov models (HMMs)*, which are widely and successfully used in the large field of bioinformatics. Briefly, SCFGs extend on traditional context-free grammars (CFGs) by additionally defining a (non-uniform) probability distribution on the generated structure class which is induced by the grammar parameters that can easily be derived from a given database of sample structures via maximum likelihood techniques. Notably, different SCFG designs can be used to model the same class of structures, where flexibility in model design comes from the fact that basically all distinct substructures can be distinguished and with increasing number of distinguished features, the resulting SCFG gains in both explicitness and complexity, which may result in a more realistic distribution on the modeled structure class.

Traditionally, SCFG based prediction approaches are realized by dynamic programming algorithms (DPAs) that require $O(n^{3})$ time and $O(n^{2})$ storage for identifying the most probable folding for an input sequence of length *n*. Examples for successful applications of several lightweight (i.e. small and simple) SCFGs for RNA secondary structure prediction can be found in [1] and a popular SCFG based prediction tool is for instance given by the Pfold software [2,3].

However, for a very long time, the free energy minimization (MFE) paradigm has been the most common technique for predicting the secondary structure of a given RNA sequence. The respective methods are traditionally realized by DPAs that employ a particular thermodynamic model for the derivation of the corresponding recursions. They basically require $O(n^{3})$ time and $O(n^{2})$ storage for identifying a set of candidate structures for an input sequence of length *n*. In fact, while early methods, like [4-6], computed only one structure (the MFE structure of the molecule), several more elaborate MFE based DPAs have been developed over the years for generating a set of suboptimal foldings (see, e.g., [7-9]). Some implementations are considered state-of-the-art tools for computational structure prediction from a single sequence, for instance the Mfold software [9,10] or the Vienna package [11,12].

In the traceback steps of the corresponding DPAs, base pairs are successively generated according to the energy minimization principle, such that the predicted set of suboptimal foldings often contains many structures that are not significantly different (that have the same or very similar shapes and contain mostly the same actual base pairings). To overcome these problems, several statistical sampling methods and clustering techniques have been invented over the last years that are based on the partition function (PF) approach for computing base pair probabilities as introduced in [13]. Briefly, these methods produce a statistical sample of the thermodynamic ensemble of suboptimal foldings and rely on a statistical representation of the Boltzmann-weighted ensemble of structures for a given sequence [14]. They are implemented in the widely used Sfold package [15].

In fact, over the past years, statistical approaches to RNA secondary structure prediction have become an attractive alternative to the standard energy-based approach (which basically relies on several thousands of experimentally-determined energy parameters). In principle, many of these approaches – in contrast to Sfold – rely on (thermodynamic) parameters estimated from growing databases of structural RNAs. For instance, the CONTRAfold tool [16] is based on a *discriminative* statistical method and uses a simplified nearest neighbor model for the underlying *conditional log-linear model (CLLM)*. Briefly, CLLMs are flexible *discriminative* probabilistic models that generalize upon more intuitive *generative* probabilistic models (like vanilla SCFGs or HMMs), where any SCFG has an equivalent representation as an appropriately parameterized CLLM. The prime advantage of using discriminate instead of generative training is that more complex scoring schemes can be considered, whereas generative models are generally easier to train and use. Actually, CONTRAfold in several cases manages to provide the highest single sequence prediction accuracy to date and eventually closes the performance gap between the best thermodynamic methods and the best (lightweight) SCFGs. However, there are some benchmarks that show better performance by other methods, suggesting in the least that the performance of structure prediction can vary considerably depending on RNA family [17-19].

Notably, following CONTRAfold, several other statistical methods have been subsequently developed, such as for instance *constraint generation (CG)*[20], or ContextFold [21]. These are all classified as discriminative statistical methods which implement different variants of standard thermodynamic models. In fact, they condition on a set of RNA sequences being given (in order to obtain estimates for the free energy parameters), whereas a generative SCFG approach models the probabilities of the input RNA sequences (in order to induce corresponding ensemble distributions).

Anyway, statistical methods for RNA folding have previously been chosen to be either purely physics-based (e.g., Sfold) or discriminative and implementing a thermodynamic model (e.g., CONTRAfold), not generative. This might have been due to the misconception that SCFGs could not easily be constructed to mirror energy-based models (as mentioned e.g. in [16]), although it has been demonstrated lately that this is actually possible (see, e.g. [22]).

However, a generative statistical method for predicting RNA secondary structure has recently been proposed [23]. This method builds on a novel probabilistic sampling approach for generating random candidate structures for a given input sequence that is based on a sophisticated SCFG design. Basically, it generates a statistical sample of possible foldings for the given sequence that is guaranteed to be representative with respect to the corresponding ensemble distribution implied by the parameters of the underlying SCFG. Particularly, conditional sampling probabilities for randomly creating unpaired bases and base pairs on actual sequence fragments are considered that are calculated by using only the grammar parameters and the corresponding inside and outside probabilities for the sequence. As the underlying elaborate SCFG mirrors the thermodynamic model employed in the Sfold software, this sampling algorithm represents a probabilistic counterpart to the sampling extension of the PF approach (as implemented in Sfold). In fact, the sole difference is that it incorporates only comprehensive structural features and additional information obtained from trusted databases of real-world RNA structures instead of the recent thermodynamic parameters.

Lately, in an attempt to improve the quality of generated sample sets, this probabilistic sampling approach has been extended to being capable of additionally incorporating *length-dependencies*[24]. In particular, the employed (heavyweight) SCFG has been transformed into a corresponding *length-dependent stochastic context-free grammar (LSCFG)* and parts of the respective procedures have been modified accordingly (in order to deal with this grammar extension). LSCFGs have been formally introduced in [25], where the main difference to conventional SCFGs is that the lengths of generated substructures are taken into account when learning the grammar parameters, yielding a more explicit structure model induced by the resulting length-dependent probabilistic parameters. Note that in connection with problems related to RNA structure, the idea of considering computational methods that actually depend on the lengths of particular substructures is not only motivated by biological aspects but has also been discussed or applied by other authors (see, e.g., [26,27]).

It remains to mention that although all three sampling approaches (PF, SCFG and LSCFG based variants) need $O(n^{3})$ time and $O(n^{2})$ storage for the generation of a statistically representative sample for an input sequence of length *n*, they obviously use different ways to define a distribution on the ensemble of all feasible secondary structures for the sequence. Applications to structure prediction (with respect to sensitivity and PPV, as well as to the shapes of sampled structures and predictions) showed that none of these sampling variants generally yields the most realistic results. Actually, which one of them should be preferred seems to strongly depend on the RNA type of the input sequence, but most importantly on the quality of a corresponding training set and on the performance of the thermodynamic model on such RNAs. However, if the worst-case complexity of one of these variants could be improved without significant losses in sampling quality (that is, if any of them required less time or space than the others while it sacrificed only little predictive accuracy), then the corresponding method would be undoubtably the number one choice for RNA structure prediction, outperforming most if not all computational tools for predicting the secondary structure of a single sequence.

For these reasons, the main objective of this paper is given as follows: We will consider the (L)SCFG based statistical sampling approach from [23,24] in order to perform a comprehensive experimental analysis on the influence of disturbances (in the considered conditional sampling distributions) on the quality of generated sample sets. Particularly, we want to explore to what extend the quality of produced secondary structure samples for a given input sequence and the corresponding predictive accuracy decreases when different degrees of disturbances are incorporated into the needed sampling probabilities. Note that some exemplary intuitive first results and corresponding observations have already been presented and discussed in [28], where it is strongly suggested that a much more meaningful evaluation based on more substantial results (with respect to several reasonable applications that are of great interest in connection with sampling approaches) is needed to be able to draw reliable conclusions.

The prime motivation for such a disturbance analysis lies in the following facts: Suppose both the samples and predictive results are observed to behave rather resistant even with respect to large errors in the distinct sampling probabilities (compared to the exact values). Then it seems adequate to believe that the sampling procedure does not have to calculate these probabilities in the exact way, but it may efficiently suffice if they are only (adequately) approximated. Thus, in this case it might obviously be possible to employ an approximation algorithm (or at least a heuristic method) for sampling probability calculations in order to decrease the worst-case time (and maybe also space) requirements for statistical sampling and hence finally for structure prediction. Furthermore, to ensure that the quality of the generated sample sets and the predictive accuracy remains sufficiently high, analysis results on the effects of different disturbance levels and types should be taken into account for the development of an appropriate approximation scheme (or heuristic). From the other perspective, suppose the quality of sampled structures seems to strongly react on rather slight disturbances already. In that case, there is obviously little hope that the worst-case complexities of the sampling method can be improved by finding a suitable heuristic procedure for the computation of the needed sampling probabilities.

The aim of our study might hence be declared as to prove or disprove the hypothesis that a heuristic method could be implemented to improve the worst-case complexity of single sequence RNA structure prediction, and to discuss some potential ideas and inherent drawbacks that seem relevant in connection with still guaranteeing highly accurate results. Although existing algorithms are in practice quite fast on any sequence for which reasonable structure prediction accuracy is expected (e.g., it takes less than an hour to predict the thermodynamic PF for a 23S rRNA of 2500 nucleotides), sacrificing little accuracy might still be assumed worthwhile, given the practical speedup of efficient heuristic methods compared the corresponding exact (non-heuristic) algorithms (e.g., the conference paper [28] reports that inside-outside calculations are indeed highly accelerated by approximation).

Note that since for any input sequence, the time (and space) complexities are dominated by those of the inside-outside computations (realized by a corresponding DPA which inherently scales $O(n^{3})$ in time and needs $O(n^{2})$ storage), the most straightforward way for reducing the time complexity of the overall sampling algorithm might be based on an efficient approximation algorithm or heuristic method for deriving the inside and outside values of the input sequence. Therefore, we will incorporate disturbances into these values (that need to be derived for any input sequence) rather than into the underlying grammar parameters (transition and emission probabilities trained on a suitable RNA database). This means that in this work, the source of an error will not come from a flawed learning set, although the study of random errors in the applied grammar parameters would actually be analogous to tests performed in connection with the thermodynamic PF [29]. The justification for a disturbance study as aspired in this article is that the parameters of the (L)SCFG underlying the statistical sampling algorithm from [23,24] might be assumed to be available (or if not, can be estimated beforehand in a single training step and might then be used for numerous input sequences). For this reason, applying random errors on the inside and outside values seems to be a much better test in the context of investigations on the impact of a performance improving heuristic.

As we will see subsequently, the (L)SCFG based statistical sampling algorithm strongly reacts to any kind of rather small absolute errors already, whereas its reaction even to rather large relative disturbances is in most cases indeed fair enough to still obtain samples of acceptable quality and corresponding meaningful structure predictions. Hence, it seems possible that a reduction of the worst-case time requirements of the evaluated probabilistic sampling approach might be reached – without sacrificing too much predictive accuracy – by approximating the needed sampling probabilities in an appropriate way. Throughout this article, we will actually present some useful considerations on how a corresponding approximation scheme (or heuristic procedure) should be constructed in order to ensure that the sampling quality remains sufficiently high.

The rest of this paper is organized as follows: Section Methods introduces the formal framework, including the (L)SCFG model, definitions of various types and levels of disturbances and a corresponding recursive sampling strategy that will be considered within this article. A comprehensive disturbance analysis based on exemplary RNA data and the corresponding results will follow in Section Results and Discussion, where both the quality of generated sample sets and their applicability to the problem of RNA structure prediction are investigated. Notably, we not only compare different ways for extracting predictions from generated samples in order to assess the predictive accuracy, but also present results on the abstraction level of shapes that is of great interest and relevance for biologists. Section Results and Discussion also includes considerations on how to develop a corresponding time-reduced sampling strategy without significant losses in sampling quality. Notably, some of the key results are discussed in Section Errors Only on Particular Values. Finally, Section Conclusions concludes the paper.

## Methods

In this section, we provide all needed information and introduce the formal framework that will be used subsequently. We start by a recap of the relevant details of the probabilistic sampling method from [23,24] and proceed with formally defining how a number of different types and levels of disturbances can be incorporated into the corresponding (L)SCFG based statistical sampling variants. Last but not least, we present a modified version of the employed sampling strategy that (contrary to the original one) manages to deal with disturbed ensemble distributions.

Note that we assume the reader to be familiar with the notions and basic concepts regarding SCFGs. A fundamental introduction on stochastic context-free languages can be found in [30]. Moreover, since for the understanding of this paper, no additional information on length-dependent stochastic models is needed, we refer to [25] for details.

### Sampling based on (L)SCFG model

In general, probabilistic sampling based on a suitable (L)SCFG has two basic steps: The first step (preprocessing) computes the inside and outside probabilities for all substrings of a given input sequence based on the considered (L)SCFG model. The second step (structure generation) takes the form of a recursive sampling algorithm to randomly draw a complete secondary structure by consecutively sampling substructures (defined by base pairs and unpaired bases) according to conditional sampling probabilities for particular sequence fragments that strongly depend on the inside and outside values derived in step one.

#### Step One – Preprocessing

According to the traditional DPA approach for predicting RNA structure via (L)SCFGs, a particular underlying grammar, say $G_{r}$, must be constructed to generate all possible RNA sequences of any length (i.e., the language $L_{r}$ of all non-empty strings over the alphabet $\Sigma_{G_{r}}:=\{A,C,G,U\}$), where any derivation tree for a particular sequence $r\in L_{r}$ corresponds to one of the feasible secondary structures (according to certain structural constraints like for instance to absence of pseudoknots, as well as with respect to preliminary defined rules for base-pairing) for *r*. This means any such (inevitably ambiguous) grammar $G_{r}$ basically relies on an appropriately designed (typically unambiguous) grammar $G_{s}$ modeling the corresponding secondary structures (i.e., the language $L_{s}$ of all corresponding words over $\Sigma_{G_{s}}:=\{(\;,)\;,\;\circ \;\}$, where () and ∘ represents any of the possible base pairs and unpaired bases, respectively, see [31]). For our investigations, we decided to rely on a rather elaborate (L)SCFG design, namely the exact formal language counterpart to the thermodynamic model applied in the Sfold program, which is given as follows:

##### 

**Definition 2.1 **([23,24]). The (length-dependent) SCFG $G_{s}$ generating exactly all secondary structures is given by $G_{s}=(I_{G_{s}},\Sigma_{G_{s}},R_{G_{s}},S)$, where $I_{G_{s}}=\;\{S,T,C,A,P,L,F,H,G,B,M,O,N,U,Z\}$, $\Sigma_{G_{s}}=\{(\;,)\;,\;\circ \;\}$ and for *m*_*h*_:= min_*HL*_ ≥ 1 and *m*_*s*_:= min_hel_ ≥ 1, $R_{G_{s}}$ contains exactly the following rules:

$$\begin{array}{l} p_{1}\;:\;S\to T,⇝\text{initiate exterior loop}\\ p_{2}\;:\;T\to C,\;\;p_{3}\;:\;T\to A,\;\;p_{4}\;:\;T\to \text{CA},\;\;p_{5}\;:\;T\to \text{AT},\\ \;\;p_{6}\;:\;T\to \text{CAT},⇝\text{shape of exterior loop}\\ p_{7}\;:\;C\to \text{ZC},\;\;p_{8}\;:\;C\to Z,\;\;⇝\text{strands in exterior loop}\\ p_{9}\;:\;A\to {\left({\;}^{m_{s}}L\right)}^{m_{s}},⇝\text{initiate helix}\\ p_{10}\;:\;P\to \left(L\right),⇝\text{extend helix}\\ p_{11}:\;L\to F,\;\;p_{12}\;:\;L\to P,\;\;p_{13}\;:\;L\to G,\\ p_{14}\;:\;L\to M,⇝\text{initiate any loop}\\ p_{15}\;:\;F\to Z^{m_{h}-1}H,\;\;⇝\text{start hairpin loop}\\ p_{16}\;:\;H\to \text{ZH},\;\;p_{17}\;:\;H\to Z,⇝\text{extend hairpin loop}\\ p_{18}:\;G\to \text{BA},\;\;p_{19}\;:\;G\to \text{AB},\;\;p_{20}\;:\;G\to \text{BAB},\\ \;\;⇝\text{shape of bulge/interior loop}\\ p_{21}\;:\;B\to \text{ZB},\;\;p_{22}\;:\;B\to Z,⇝\text{strands in bulge/interior}\\ \;\;\;\text{loop}\\ p_{23}\;:\;M\to \text{UAO},⇝\text{fist substructure of multiple loop}\\ p_{24}\;:\;O\to \text{UAN},⇝\text{second substructure of multiple}\\ \;\;\text{loop}\\ p_{25}\;:\;N\to \text{UAN},\;\;p_{26}:\;N\to U,⇝k\text{th substructure of}\\ \;\;\;\text{multiple loop},k\geq 3\\ p_{27}\;:\;U\to \text{ZU},\;\;p_{28}\;:\;U\to \varepsilon ,⇝\text{strands in multiple loop}\\ p_{29}\;:\;Z\to \circ .⇝\text{unpaired base} \end{array}$$

Note that $G_{s}$ has been parameterized to impose two relevant restrictions on the class of all feasible structures: first, a minimum length of min_*HL*_ for hairpin loops and second, a minimum number of min_hel_ consecutive base pairs for helices, where common choices are min_*HL*_ ∈ {1, 3} and min_hel_ ∈ {1, 2}. However, within this work we will only consider min_*HL*_= min_hel_ = 1, which corresponds to the least restrictive (yet also most unrealistic) choice and usually yields the worst sampling results (see [23,24]).

Moreover, the needed grammar parameters (trained on a suitable RNA structure database) are splitted into a set of *transition probabilities* Pr_*tr*_(*rule*) for *rule*$\in \;R_{G_{s}}$ and two sets of *emission probabilities* Pr_*em*_(*r*_*x*_) for $r_{x}\in \Sigma_{G_{r}}$ and ${\text{Pr}}_{\text{em}}(r_{x_{1}}r_{x_{2}})$ for $r_{x_{1}}r_{x_{2}}\in \Sigma_{G_{r}}^{2}$, i.e. for the 4 unpaired bases and the 16 possible base pairings, respectively. It should be mentioned that in the length-dependent case, these probabilities depend on the length of the subwords generated, meaning we then have to use Pr_*tr*_(*rule*, *len* = len(*rule*)), where len(*rule*) denotes the length of a specific application of *rule* in a parse tree, which is defined as the length of the (terminal) subword eventually generated from *rule*. Accordingly, we need to consider Pr_*em*_(*r*_*x*_, *len* = 1) and ${\text{Pr}}_{\text{em}}(r_{x_{1}}r_{x_{2}},\text{len}=x_{2}-x_{1}+1)$, respectively. Note that for the sake of simplicity, we will omit the length (second parameter) in the sequel, hence using the same notations in either case (length-dependent or not).

However, according to [23,24], the computation of all inside probabilities

(1) $$\alpha_{X}(i,j):=\text{Pr}(X\Rightarrow_{\text{lm}}^{*}r_{i}\ldots r_{j})$$

and all outside probabilities

(2) $$\beta_{X}(i,j):=\text{Pr}(S\Rightarrow_{\text{lm}}^{*}r_{1}\ldots r_{i-1}Xr_{j+1}\ldots r_{n})$$

for a sequence *r* of size *n*, $X\in I_{G_{s}}$ and 1 ≤ *i*, *j* ≤ *n*, can be done with a special variant of an Earley-style parser (such that the considered grammar does not need to be in *Chomsky normal form (CNF)*). Notably, both sampling variants (length-dependent or not) can be implemented to require $O(n^{3})$ time and $O(n^{2})$ memory for this preprocessing step.

#### Step Two – Random structure generation

Once the preprocessing is finished, different strategies may be employed for realizing the recursive sampling step. In general, for any sampling decision (for example choice of a new base pair), a particular strategy relies on the respective set of all possible choices that might actually be formed on the currently considered fragment of the input sequence. Any of these sets contains exactly the mutually exclusive and exhaustive cases as defined by the alternative productions (of a particular intermediate symbol) of the underlying grammar. The corresponding random choice is then drawn according to the resulting conditional sampling distribution (for the considered sequence fragment). This means the respective sampling distributions are defined by the inside and outside values derived in step one (providing information on the distribution of all possible choices according to the actual input sequence) and the grammar parameters (transition probabilities).

In this work, we will only consider the well-established strategy from [23,24], which is also implemented in the corresponding second step of the physics-based sampling algorithm underlying the popular Sfold tool. Basically, a secondary structure is sampled recursively by starting with the entire RNA sequence and consecutively computing the adjacent substructures (single-stranded regions and paired substructures) of the exterior loop (from left to right), where any paired substructure is completed by successively folding other loops. In fact, the base pairs and unpaired base(s) are successively sampled according to conditional probability distributions for the considered fragment, given a partially formed structure.

For example, suppose fragment *R*_*i*, *j*_:= *r*_*i*_ … *r*_*j*_ of input sequence *r*, 1 ≤ *i*, *j* ≤ *n* = |*r*|, is to be folded, where it is known that the resulting substructure on *R*_*i*,*j*_ must correspond to a (valid) derivation of a particular intermediate symbol $X\in I_{G_{s}}$ (according to the partially formed structure). Then, the strategy considers the corresponding set *acX*(*i*, *j*) of all choices for (valid) derivations of *X* on *R*_*i*,*j*_, which actually correspond to all possible substructures on *R*_*i*,*j*_ (the mutually exclusive and exhaustive cases for *X* on *R*_*i*,*j*_). Under the assumption that the alternatives for intermediate symbol *X* are equal to *X* → *Y* and *X* → *VW*, this set is defined as follows:

(3) $$\text{acX}(i,j):={\text{acX}}_{Y}(i,j)\cup {\text{acX}}_{\text{VW}}(i,j),$$

where

$$\begin{array}{lll} \;{\text{acX}}_{Y}(i,j): & =\{\text{prob}|\text{prob}=\beta_{X}(i,j)\cdot \alpha_{Y}(i,j)\; & \;\\ & \;\times {\text{Pr}}_{\text{tr}}(X\to Y)\neq 0\}\; & \;\\ & =\{\beta_{X}(i,j)\cdot \text{prob}|\beta_{X}(i,j)\neq 0\;\text{and}\;\; & \;\\ \text{prob}\;\;\; & =\alpha_{Y}(i,j)\cdot {\text{Pr}}_{\text{tr}}(X\to Y)\neq 0\}\; & \; \end{array}$$

and

$$\begin{array}{lll} \;{\text{acX}}_{\text{VW}}(i,j) & :=\{\{k,\text{prob}\}|i\;\leq \;k\;\leq \;j\;\text{and}\;\text{prob}=\beta_{X}(i,j)\; & \;\\ & \;\times \alpha_{V}(i,k)\;\cdot \;\alpha_{W}(k+1,j)\;\cdot \;{\text{Pr}}_{\text{tr}}(X\;\to \;\text{VW})\; & \;\\ & \;\neq 0\}\; & \;\\ & =\{\{k,\beta_{X}(i,j)\cdot \text{prob}\}|i\leq k\leq j\;\text{and}\; & \;\\ \beta_{X}(i,j)\;\;\;\; & \neq 0\;\text{and}\;\text{prob}=\alpha_{V}(i,k)\cdot \alpha_{W}(k+1,j)\; & \;\\ & \;\times {\text{Pr}}_{\text{tr}}(X\to \text{VW})\neq 0\}.\; & \; \end{array}$$

Consequently, we have to sample from the corresponding conditional probability distribution induced by *acX*(*i*, *j*), that is the random choice is drawn according to the following set of sampling probabilities:

(4) $$\;\;\left\{\frac{\text{prob}}{\text{norm}}|\text{prob}\in {\text{acX}}_{Y}(i,j)\;\text{or}\;\{k,\text{prob}\}\in {\text{acX}}_{\text{VW}}(i,j)\right\},$$

where obviously,

(5) $$\underset{\text{prob}\in {\text{acX}}_{Y}(i,j)}{\sum }\frac{\text{prob}}{\text{norm}}+\underset{\left\{k,\text{prob}\}\in {\text{acX}}_{\text{VW}}(i,j\right)}{\sum }\frac{\text{prob}}{\text{norm}}=1$$

must hold, which can in general easily be guaranteed by using *norm* = *β*_*X*_(*i*,*j*) · *α*_*X*_(*i*, *j*). However, if there may occur inconstancies in the distribution induced by the underlying grammar model (for example if a particular implementation faces problems that arise from numerical imprecisions or if the distribution has been deliberately disturbed as we intend to do in the sequel), we should instead use

$$\begin{array}{lll} \;\text{norm} & =\underset{\text{prob}\in {\text{acX}}_{Y}(i,j)}{\sum }\text{prob}+\underset{\left\{k,\text{prob}\}\in {\text{acX}}_{\text{VW}}(i,j\right)}{\sum }\text{prob}\; & \;\\ & =\beta_{X}(i,j)\left(\underset{\beta_{X}(i,j)\cdot \text{prob}\in {\text{acX}}_{Y}(i,j)}{\sum }\text{prob}\right)\; & \;\\ & \;+\left(\underset{\left\{k,\beta_{X}(i,j)\cdot \text{prob}\}\in {\text{acX}}_{\text{VW}}(i,j\right)}{\sum }\text{prob}\right)\; & \;\\ & =\beta_{X}(i,j)\cdot \left(\alpha_{Y}(i,j)\cdot {\text{Pr}}_{\text{tr}}(X\to Y)+\underset{i\leq k\leq j}{\sum }\alpha_{V}(i,k)\right)\; & \;\\ & \;\alpha_{W}\left((k+1,j)\cdot {\text{Pr}}_{\text{tr}}(X\to \text{VW})\;\right)\; & \;\\ & =\beta_{X}(i,j)\cdot {\text{norm}}_{\alpha },\; & \; \end{array}$$

which then ensures that the corresponding sampling probabilities still sum up to unity, such that they indeed define a conditional probability distribution).

Note that the sampling strategy effectively works conform with the SCFG model, which means that it actually samples one of the possible parse trees of the given input sequence by randomly drawing one of the respective mutually exclusive and exhaustive cases (corresponding to the distinct grammar rules with same premise) at any point in the already partially constructed parse tree in order to generate one of the possible subtrees for the given input sequence (corresponding to one the possible substructures on the considered sequence fragment, which is currently being folded recursively).

Hence, according to the sampling process, we could have never gotten to a point where we have to consider all mutually exclusive and exhaustive cases for a particular premise $X\in I_{G_{s}}$ on an actual sequence fragment *R*_*i*,*j*_, 1 ≤ *i*, *j* ≤ *n*, if the grammar could not derive the sentential form *r*_1_ … *r*_*i*-1_*Xr*_*j*+1_ … *r*_*n*_ from the start symbol (axiom) $S\in I_{G_{s}}$, that is if the outside value *β*_*X*_(*i*, *j*) would be equal to 0. This in fact means that the respective probability distribution (conditioned on the considered fragment *R*_*i*,*j*_) from which the strategy randomly samples one of the possible substructures (one valid subtree of the already partially constructed parse tree) is not influenced by the corresponding outside probability, due to the fact that *β*_*X*_(*i*, *j*) > 0 indeed only represents a scaling factor common to all sampling probabilities for the relevant mutually exclusive and exhaustive cases. For this reason, we can obviously without loss of information remove the outside values from the definitions of the needed sampling probabilities. The correctness of this simplification can easily be formally proven by considering the above defined set *acX*(*i*, *j*) of all choices for possible derivations of intermediate symbol *X* on sequence fragment *R*_*i*,*j*_. In fact, the sampling strategy randomly draws one of the elements from *acX*(*i*, *j*) according to the corresponding distribution induced by normalizing the probabilities of the elements in *acX*(*i*, *j*) such that they sum up to unity. Particularly, we have

$$\begin{array}{lll} 1 & =\underset{\beta_{X}(i,j)\cdot \text{prob}\in {\text{acX}}_{Y}(i,j)}{\sum }\frac{\beta_{X}(i,j)\cdot \text{prob}}{\beta_{X}(i,j)\cdot {\text{norm}}_{\alpha }}\; & \;\\ & \;+\underset{\left\{k,\beta_{X}(i,j)\cdot \text{prob}\}\in {\text{acX}}_{\text{VW}}(i,j\right)}{\sum }\frac{\beta_{X}(i,j)\cdot \text{prob}}{\beta_{X}(i,j)\cdot {\text{norm}}_{\alpha }}\; & \;\\ & =\frac{1}{{\text{norm}}_{\alpha }}\cdot \left(\underset{\beta_{X}(i,j)\cdot \text{prob}\in {\text{acX}}_{Y}(i,j)}{\sum }\text{prob}\right)\; & \;\\ & \;+\left(\underset{\left\{k,\beta_{X}(i,j)\cdot \text{prob}\}\in {\text{acX}}_{\text{VW}}(i,j\right)}{\sum }\text{prob}\right)\; & \;\\ & =\frac{1}{{\text{norm}}_{\alpha }}\cdot \left(\underset{\text{prob}\in {\text{acX}}_{Y}(i,j)}{\sum }\frac{\text{prob}}{\beta_{X}(i,j)}\right)\; & \;\\ & \;+\left(\underset{\left\{k,\text{prob}\}\in {\text{acX}}_{\text{VW}}(i,j\right)}{\sum }\frac{\text{prob}}{\beta_{X}(i,j)}\right),\; & \; \end{array}$$

since *β*_*X*_(*i*, *j*) ≠ 0 holds (due to the definitions of *acX*_*Y*_(*i*, *j*) and *acX*_*VW*_(*i*, *j*)).

Formal definitions of all corresponding sets *acX*(*i*, *j*), $X\in I_{G_{s}}$ and 1 ≤ *i*, *j* ≤ *n*, that are considered by the recursive sampling strategy for any input sequence of length *n*, including formulae for deriving the respective conditional sampling probabilities, can be found in Section Sm-I^a^ (of Additional file 1). Notably, all those formulae only depend on some of the parameters of the underlying (L)SCFG model and the corresponding inside values, such that after a preprocessing of the given sequence (which includes the complete inside computation and needs $O(n^{3})$ time in the worst-case), a random candidate structure can be generated in $O(n^{2})$ time.

### Considered disturbance types and levels

Obviously, under the assumption of a particular (L)SCFG model (trained beforehand on arbitrary RNA data), the most straightforward way for improving the performance of the corresponding overall sampling algorithm seems to be by reducing the worst-case complexity of the inside calculations. Therefore, we decided to quantify to which extend the algorithm reacts to different types and degrees of disturbances incorporated into the considered inside probabilities in order the get evidence if it could actually be possible to find a corresponding approximation algorithm (or at least an appropriate heuristic method) that eventually requires less time but causes only acceptable losses in accuracy. In fact, with respect to developing a suitable heuristic method to be applied in practice, it is necessary to know about the effects of different disturbance levels and types to get an idea on how precisely the respective values need to be approximated in order to guarantee sufficiently good results and to find out which types of errors pose fundamental problems and which ones are negligible.

For these reasons, given an arbitrary input sequence *r* of length *n*, we decided to consider (more or less) skewed inside probabilities^b^

(6) $${\hat{\alpha }}_{X}(i,j):=\max(\min(\alpha_{X}(i,j)+\alpha_{X}^{\text{err}}(i,j),1),0),$$

for $X\in I_{G_{s}}$ and 1 ≤ *i*, *j* ≤ *n*, rather than the corresponding correct values *α*_*X*_(*i*, *j*) (obtained in the preprocessing step for *r*) for defining the needed sampling probabilities. More precisely, we want to incorporate different stages of (more or less grave) randomly chosen errors into particular inside values for the given sequence, that is into preliminary chosen subsets of the set of all precomputed inside probabilities *α*_*X*_(*i*, *j*), $X\in I_{G_{s}}$ and 1 ≤ *i*, *j* ≤ *n*. Note that is actually suffices to consider $X\in I_{G_{s}}^{\alpha }:=\{T,C,A,P,F,G,B,M,O,N,U\}\subset I_{G_{s}}$, since only those intermediate symbols are needed for defining the diverse sampling probabilities that are used by the employed sampling strategy for obtaining the distinct conditional distributions for drawing particular random choices.

However, in order to reach our previously declared goal, for any fixed value *prob* ∈ (0, 1]^c^, we decided to draw $\alpha_{X}^{\text{err}}(i,j)$ (uniformly) at random from either of the following sets:

(7) $$\;\text{fun}c_{I}^{\text{win},\text{op}}(\text{prob}):=\left\{\begin{array}{l} \text{Interval}(\text{func}),\;\text{if}\;X\in I\subseteq I_{G_{s}}^{\alpha }\\ \text{and}\;[(j\;-\;i\;+\;1\;>\;\text{win}\;\text{and}\;\text{op}\;=\;+)\;\text{or}\\ \;\;\;(j-i+1\leq \text{win}\;\text{and}\;\text{op}=-)]\;,\\ \{0\},\;\text{else}, \end{array}\right)$$

such that only inside values of particularly chosen intermediate symbols that lie outside (*op*= +) or within (*op*= -) a considered window of preliminary fixed size are actually disturbed, that is only for those values ${\hat{\alpha }}_{X}(i,j)\neq \alpha_{X}(i,j)$ might result. Notably, *Interval*(*func*) is not centered on *α*_*X*_(*i*, *j*), as it actually describes the set of error values $\alpha_{X}^{\text{err}}(i,j)$ that might be drawn (uniformly) at random – which are then added to *α*_*X*_(*i*, *j*). Anyway, in the sequel, we will basically consider either

(8) $$\text{fun}c^{\text{win},\text{op}}(\text{prob}):=\text{fun}c_{I_{G_{s}}^{\alpha }}^{\text{win},\text{op}}(\text{prob})$$

(i.e., disturbances only inside or outside fix-sized window, but for all intermediate symbols),

(9) $${\text{func}}_{I}(\text{prob}):=\text{fun}c_{I}^{n,+}(\text{prob})=\text{fun}c_{I}^{-1,-}(\text{prob})$$

(i.e., errors for all subword lengths, but only for particular intermediate symbols), or simply

(10) $$\text{func}(\text{prob}):=\text{fun}c_{I_{G_{s}}^{\alpha }}^{n,+}(\text{prob})=\text{fun}c_{I_{G_{s}}^{\alpha }}^{-1,-}(\text{prob})$$

(i.e., disturbances on all considered inside values).

Moreover, *func* ∈ {mep, fep, mev, fev} denotes the actual disturbance type. Principally, we distinguish between two degrees of errors: relative and absolute ones. To generate relative errors, we might either use *func* = mep (which stands for *maximum allowed error percentage*, with respect to the corresponding correct value) or *func* = fep (for *fixed error percentage*, which is ought to force greater and hence more severe random errors). Formally, this means that either

(11) $$\text{Interval}(\text{mep}):=[-\text{prob}\cdot \alpha_{X}(i,j),+\text{prob}\cdot \alpha_{X}(i,j)]$$

or

(12) $$\text{Interval}(\text{fep}):=\{-\text{prob}\cdot \alpha_{X}(i,j),+\text{prob}\cdot \alpha_{X}(i,j)\}$$

might be employed for randomly drawing a relative error $\alpha_{X}^{\text{err}}(i,j)$, where *prob* ∈ (0, 1] indeed defines the desired percentage. Note that the consideration of symmetric intervals (as defined by *Interval*(mep)) is of interest as it models the case that all errors $\alpha_{X}^{\text{err}}(i,j)$ are bounded but do not need to admit the maximum value possible (according to *prob*). When studying relative errors in connection with this variant, this basically corresponds to assuming a particular approximation ratio of the underlying algorithm. The consideration of discrete sets (as defined by *Interval*(fep)) corresponds to the case that any error takes on the maximum value possible (according to *prob*). This variant hence explicitly describes the worst-case (by means of magnitudes of incorporated errors) of the symmetric interval variant and is actually of interest as it enables a more reliable study of the influence of disturbances, particularly in cases where the extenuated symmetric interval variant defined by mep seems to have no effect on the resulting accuracy.

For similar reasons, in order to randomly choose an absolute error $\alpha_{X}^{\text{err}}(i,j)$ for obtaining a (potentially) disturbed probability ${\hat{\alpha }}_{X}(i,j)$, we might equivalently consider either

(13) Interval(mev):=[-prob,+prob]

or

(14) Interval(fev):={-prob,+prob},

with *prob* ∈ (0, 1] being a preliminary fixed value. This means we may use *func* = mev (which stands for *maximum allowed error value*, independent on the corresponding correct value) and *func* = fev (for *fixed error value*, usually resulting in more grave disturbances) for causing absolute disturbances.

Note that random errors on all outside probabilities *β*_*X*_(*i*, *j*), $X\in I_{G_{s}}$ and 1 ≤ *i*, *j* ≤ *n*, could be generated in basically the same way, but since those values can be deliberately excluded from the definition of sampling probabilities (according to the employed sampling strategy), this is obviously not necessary for the subsequent investigations.

Finally, it should be clear that for *func* ∈ {mep, fep} (resulting in relative errors), only the magnitudes of the corresponding sampling probabilities (with respect to the implied skewed conditional sampling distributions) change, such that the exact same structures are possible as in the undisturbed case. Hence, we might expect that only the consideration of sufficiently large percentages *prob* ∈ (0, 1] for generating errors according to $\text{fun}c_{I}^{\text{win},\text{op}}(\text{prob})$ can cause an actual shifting in the ensemble distribution, resulting in significant quality losses. The contrary holds for absolute errors created according to $\text{fun}c_{I}^{\text{win},\text{op}}(\text{prob})$ with *func* ∈ {mev, fev}. In fact, since the (cardinalities of the) respective sets of relevant sampling choices implied by the skewed ensemble distribution generally differ (to a more or less severe extent) from the corresponding exact ones, it must be expected that only rather small fixed error values of *prob* ∈ (0, 1] are reasonable choices for our purpose. However, since for distinct subword lengths *j* - *i* + 1, 1 ≤ *i*, *j* ≤ *n*, the corresponding probabilities *α*_*X*_(*i*, *j*) for any $X\in I_{G_{s}}^{\alpha }$ usually imply different orders of magnitudes^d^, it seems practically impossible to tell how to find an appropriate fixed error value for creating absolute disturbances.

### Resulting modified sampling strategy

It should be clear that after the desired errors (according to any of the previously specified variants of either mep,fep,mev or fev) have been incorporated into the precomputed exact inside (and outside) values for a given sequence, the needed conditional sampling distributions (as considered by a particular strategy) are induced by the exact grammar parameters and the disturbed inside (and outside) probabilities for that sequence. This, however, might create the need to (slightly) modify the respective particularly employed sampling strategy such that it finally gets capable to deal with these skewed distributions.

As for this work, consider the previously sketched recursive sampling strategy from [23,24]. Without any errors in the conditional probability distributions (i.e. by using the exact probabilistic parameters for the given input sequence, particularly the corresponding inside values), it always successfully generates the sampled loop type for a considered sequence fragment. For example, suppose the sampling procedure decides that base pair *r*_*i*_.*r*_*j*_ should close a multiloop, then the sequence fragment *R*_*i*+1,*j*-1_:= *r*_*i*+1_ … *r*_*j*-1_ is guaranteed to be folded into an admissible multiloop that by definition contains at least two helical regions radiating out from this loop. However, by using disturbed sampling probabilities (given by the exact parameters of the underlying (L)SCFG model and disturbed inside values for input sequence *r*, derived by incorporating any sort of errors), the sampling algorithm may choose to form a particular substructure on the fragment *R*_*i*+1,*j*-1_, although this would actually not be possible.

**Figure 1:**
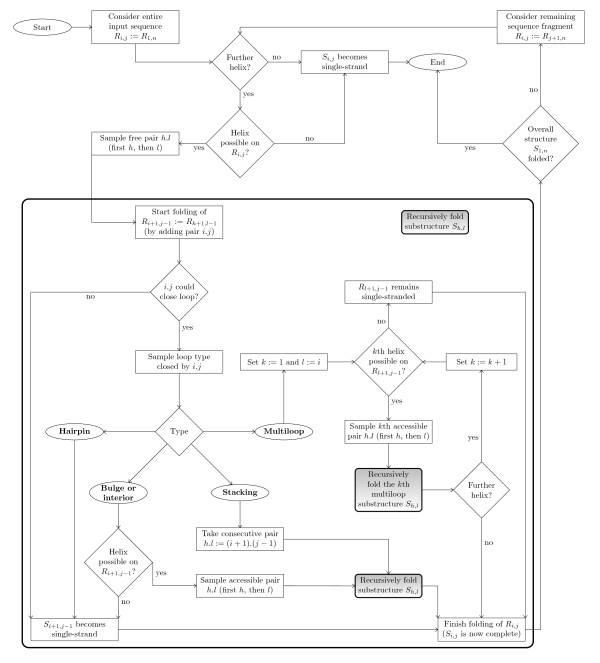
**Flowchart for recursive sampling of an RNA secondary structure *S***_**1** ***,n ***_**for a given input sequence *r *** **of length *n *** **according to an inherently controlled strategy with predetermined order, similar to that of **[14]**,**[23]**.**

Therefore, we had to slightly modify the sampling procedure such that in any case where the chosen substructure type can not be successfully generated, it settles for the partially formed substructure. That is, it either leaves the complete fragment unpaired (if the desired base pairs could not be sampled at all), or else it for example only creates a bulge/interior loop although a multiloop should have been constructed (but only one helix has been successfully sampled). The resulting modified versions of the distinct sampling steps (in pseudocode) are given in Section Sm-I (of Additional file 1), Figure 1 gives a schematic overview of the overall sampling process.

Note that alternatively, the algorithm could have been modified to revise any decisions that lead to incompletely generated substructures, resulting in some sort of backtracking procedures that obviously would have to be applied in order to sample more realistic overall structures for a given RNA sequence. However, as this effectively results in much more complex modifications and eventually yields significant losses in performance, we opted for the simpler and more straightforward first variant to get rid of the described problem.

## Results and discussion

The aim of this section is to perform a comprehensive experimental analysis on the influence of disturbances (in the ensemble distribution for a given input sequence) on the quality of sample sets generated by the (L)SCFG based statistical sampling approach from [23,24]. In fact, we want to explore to what extend the quality of produced secondary structure samples for a given input sequence and the corresponding predictive accuracy decreases when different degrees of errors are incorporated into the needed sampling probabilities.

### RNA structure data

For our examinations, we decided to consider different sets of trusted RNA secondary structure data for which the (L)SCFG based sampling approach yields good quality results when no disturbances are included in the respective sampling distributions for a given sequence. Therefore, we took the same tRNA database (of 2163 distinct tRNA structures with lengths in [64, 93] and about 76 on average, derived from [32]) and the identical 5S rRNA data set (of 1149 distinct sequences with lengths in [102, 135] and about 119 on average, retrieved from [33]) as collected in [23]. These two rich data sets of trusted RNA secondary structures will be exclusively used as the basis for the following applications, such that the results can easily be opposed to the corresponding ones presented in [24].

### Probability profiling for specific loop types

A statistical sample of all possible secondary structures for a given RNA sequence can be used for sampling estimates of the probabilities of any structural motifs. Actually, *probability profiling* for unpaired bases within particular loop types can easily be applied for this purpose. In principle, for each nucleotide position *i*, 1 ≤ *i* ≤ *n*, of a given sequence of length *n*, one computes the probabilities that *i* is an unpaired base within a specific loop type. These probabilities are given by the observed frequencies in a random sample set.

**Figure 2:**
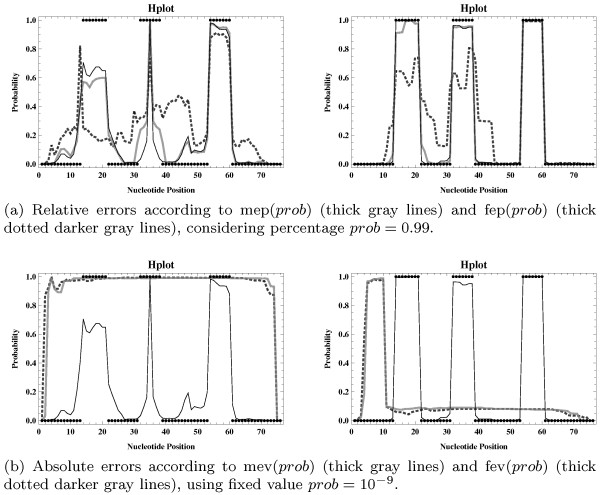
**Hairpin loop profiles for *E.coli *tRNA**^***Ala***^**, calculated from a random sample of 1000 structures generated with the SCFG (figures on the left) and LSCFG (figures on the right) approach, respectively (under the assumption of the less restrictive grammar parameters min**_***hel ***_**= 1 and min**_***HL ***_**= 1).** The exact (undisturbed) results are displayed by the thin black lines, and the correct hairpin loops in *E.coli* tRNA^*Ala*^ are illustrated by the black points.

**Figure 3:**
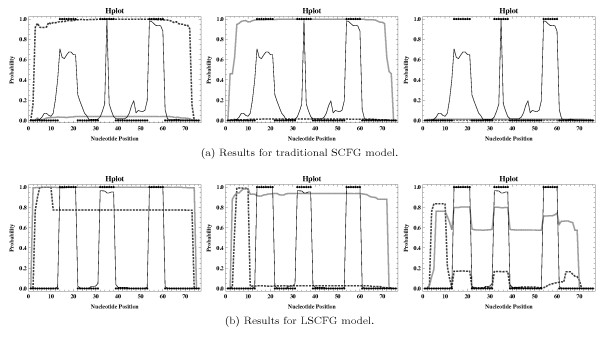
**Hairpin loop profiles corresponding to those presented in Figure **2**b, where absolute errors were derived according to mev**^***win*** **,+**^**(*prob*) (thick gray lines) and fev**^***win*** **,+**^**(*prob*) (thick dotted darker gray lines), respectively, with *prob *** **= 10**^**-9 **^**and *win *** **∈ {15, 38, 60} (figures from left to right).**

**Figure 4:**
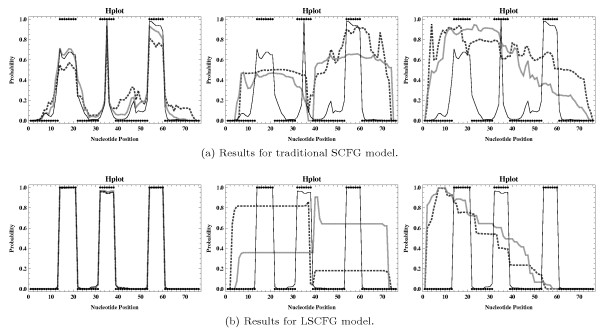
**Hairpin loop profiles corresponding to those presented in Figure **2**b, where absolute errors were derived according to mev**^***win*** **,-**^**(*prob*) (thick gray lines) and fev**^***win*** **,-**^**(*prob*) (thick dotted darker gray lines), respectively, with *prob *** **= 10**^**-9 **^**and *win *** **∈ {15, 38, 60} (figures from left to right).**

Since this application is rather intuitive, we decided to use it as a starting point for our disturbance analysis. Particularly, we derived a number of statistical samples for the well-known *Escherichia coli* tRNA^*Ala*^ sequence by applying the sampling strategy from Section Resulting Modified Sampling Strategy on the basis of diverse sets of probabilistic parameters (inside probabilities disturbed according to several particular variants as defined in Section Considered Disturbance Types and Levels) for that sequence and calculated corresponding probability profiles. All relevant results are displayed in Additional file 1: Figures S1 to S14 of Section Sm-II. Some of the potentially most interesting ones are presented in Figures 2, 3 and 4.

#### Errors on all values

Let us first consider the profiles displayed in Figure 2 (and in Additional file 1: Figures S1 and S2). Obviously, even if large relative errors on all inside probabilities and hence on the needed conditional sampling probabilities are generated, the sampled structures still exhibit the typical cloverleaf structure of tRNAs, especially for the length-dependent sampling approach where relative disturbances seem to have no significant negative effect on the sampling quality (see Figure 2a). However, Figure 2b perfectly demonstrates that if the disturbances have been created by adding absolute errors to all inside values, then – even for rather small absolute error values – the resulting samples obtained with both the SCFG and LSCFG approach are useless.

Note that for any given input sequence, it seems to be usually much more important for the employed sampling strategy to be able to identify which ones of the (combinatorially) possible substructures can actually be (validly) formed on the considered sequence fragment rather than to know their exact probabilities (according to the conditional distribution for the respective fragment), for two contrary reasons: First, in order to avoid drawing practically impossible choices, which later forces it to leave the considered sequence fragment (at least partially) unpaired^e^. Second, for ensuring that none of the actually valid choices is prohibited during the folding process, such that the sampling procedure might inevitably prefer other (potentially even impossible) substructures.

Consequently, in order to prevent a decline in accuracy of generated structures and a reduction of the overall sampling quality, it seems to be of great importance that the sampling strategy is capable of distinguishing between inside values and especially sampling probabilities that are equal and unequal to zero according to the exact (undisturbed) ensemble distribution for the given input sequence. By adding absolute errors, however, inside or sampling probabilities being equal (unequal) to zero in the exact case might often become unequal (equal) to zero according to the resulting skewed (disturbed) distributions, whereas by incorporating relative errors, all considered inside and sampling probabilities obviously stay equal or unequal to zero (as in the exact case), which intuitively explains the basic observations made from Figure 2.

#### Relevant sampling probabilities

Nevertheless, in order to draw more detailed conclusions, we counted and compared the relevant (i.e., greater than zero) inside and sampling probabilities that were considered for obtaining the profiles presented in Figure 2. The results are collected in Additional file 1: Tables S1 and S2 of Section Sm-II.

First, it seems obvious that due to the more explicit length-dependent version of the considered grammar parameters (length-dependently trained transition and emission probabilities), there should generally result a much smaller number of relevant inside values and sampling probabilities when applying the LSCFG model rather than the conventional one. Tables S1 and S2 exemplarily prove this intuitive assumption. Note that this effect might indeed be responsible for the observation that the LSCFG based sampling approach reacts considerably less to large relative errors than the conventional length-independent variant, as indicated by Figure 2a: less inside probabilities are effectively disturbed, such that the extend of the relative errors imposed on the corresponding sampling probabilities is inevitably smaller for the LSCFG variant than for the length-independent one.

Moreover, there are much more relevant exact inside and sampling probabilities than corresponding relevant disturbed values for basically any (intermediate) symbol when considering the traditional SCFG model, whereas for the LSCFG variant the contrary holds, that is generally way more inside and sampling probabilities are relevant in the disturbed cases than in the exact case. Actually, in both cases (length-dependent and not), the numbers of relevant disturbed inside values ${\hat{\alpha }}_{X}(i,j)$, 1 ≤ *i*, *j* ≤ *n*, are rather similar (for basically all $X\in I_{G_{s}}^{\alpha }$), in contrast to the numbers of relevant sampling probabilities (corresponding to valid choices for substructures) for the distinct sampling steps which are in general to a large extend greater when using the traditional SCFG approach than under the assumption of the corresponding LSCFG model. This behavior might be the reason for the fundamental differences in the resulting (albeit useless) loop profiles presented in Figure 2b.

Finally, it remains to mention that under the assumption of the conventional SCFG model, it happens that for any $X\in I_{G_{s}}^{\alpha }$, most inside values are relevant in both the exact and the disturbed case, whereas significantly less are relevant only in the exact case and very few are only relevant in the disturbed case (see Table S1a). Considering the LSCFG variant, however, for any $X\in I_{G_{s}}^{\alpha }$ the least inside values are relevant only in the exact case, as indicated by Table S1b. Obviously, this seems to be the natural consequence of the previously formulated observations.

#### Errors only on particular values

Now, in an attempt to find out in which cases particular absolute errors have a very significant (negative) impact on the resulting sampling quality and to identify potentially existing situations where they barely influence the output of the applied statistical sampling algorithm, we want to consider some of the more specialized variants for generating absolute disturbances (as defined in Section Considered Disturbance Types and Levels). The corresponding profiles are basically shown in Figures 3 and 4 (as well as in Additional file 1: Figures S3 to S14).

Notably, even if absolute disturbances may only occur for inside values *α*_*X*_(*i*, *j*), $X\in I_{G_{s}}^{\alpha }$, with *j* - *i* + 1 > *win* (i.e., for substructure lengths greater than a particular fixed value *win*), the corresponding sampling results are of no practical use at all (see Figure 3). In fact, there seem to be no noticeable improvements when considering increasing values of *win*, which means that even if more inside values *α*_*X*_(*i*, *j*), $X\in I_{G_{s}}^{\alpha }$, namely those satisfying *j* - *i* + 1 ≤ *win*, are guaranteed to be exact (contain no relative or absolute errors), the resulting samples might not be expected to gain in quality. This observation is actually unfortunate as regards the derivation of a corresponding heuristic version of the inside algorithm, since the inside computation starts by calculating the respective values for small sequence fragments and subsequently considers larger ones, meaning the straightforward approach of deriving all values *α*_*X*_(*i*, *j*), $X\in I_{G_{s}}^{\alpha }$, with *j* - *i* + 1 ≤ *win* in the exact way and approximating only the remaining ones (i.e., using a constant window size *win* for exact calculations) might not yield results of acceptable quality if absolute errors can not be ruled out (completely).

Nevertheless, as we can see from Figure 4, if absolute disturbances may only occur for inside values *α*_*X*_(*i*, *j*), $X\in I_{G_{s}}^{\alpha }$, with *j* - *i* + 1 ≤ *win* (i.e., for substructure lengths less than or equal to a particular fixed value *win*), the corresponding sampling results might actually be of acceptable quality, but seemingly only for rather small values of *win*. This means in order to obtain a practically applicable heuristic, it seems a good idea to consider a constant (small enough) window of size *win* and compute all values *α*_*X*_(*i*, *j*), $X\in I_{G_{s}}^{\alpha }$, with *j* - *i* + 1 > *win* in the exact way, thus approximating only those satisfying *j* - *i* + 1 ≤ *win*. However, due to the contrary course of action of traditional inside calculations, this approach can obviously not be realized. Consequently, this observation does not contribute to developing an appropriate heuristic variant of the preprocessing step, but it actually motivates the construction of an innovative sampling strategy that takes on a reverse sampling direction (that constructs substructures in an inside-to-outside fashion, contrary to the generation of corresponding derivation trees according to the underlying grammar).

Finally, for the sake of completeness, it should be noted that by incorporating absolute errors (for all subword lengths) only for any of the distinct intermediate symbols $X\in I_{G_{s}}^{\alpha }$ at once (i.e., by disturbing only the inside values *α*_*X*_(*i*, *j*), 1 ≤ *i*, *j* ≤ *n*, for a particular $X\in I_{G_{s}}^{\alpha }$), we found out that some are more sensitive with respect to disturbances in the underlying ensemble distribution than others (see Additional file 1: Figures S7 to S14 of Section Sm-II). In principle, the strongest (negative) reactions to the influence of the generated absolute errors were observed for symbols *T*, *C*, *A*, *F* (for the traditional SCFG model), *G* and *U*, whereas less severe quality losses basically resulted for intermediates *M*, *O*, *N* and *P*. Moreover, for two symbols, namely *F* (for the LSCFG model) and *B*, we recognized no noticeable impact of the caused disturbances to the accuracy of the generated sample sets.

### Prediction accuracy – Sensitivity and PPV

In connection with sampling approaches, there exist diverse (more or less) efficient well-defined principles for extracting a particular structure prediction from a generated set of candidate structures for a given input sequence. In fact, under the condition that a corresponding folding can be calculated in $O(n^{3})$ time and with $O(n^{2})$ storage (i.e., has the same worst-case complexities as the preprocessing step), the statistical sampling method considered in this work can easily be applied to single sequence secondary structure prediction without significant losses in performance and its predictive power can easily be measured by means of *sensitivity* (Sens.) and *positive predictive value* (PPV)^f^. Briefly, these two common measures are widely used in order to quantify the accuracy of RNA secondary structure prediction methods and are usually defined as follows (see e.g. [34]): 

• Sens. is the relative frequency of correctly predicted pairs among all position pairs that are actually paired in a stem of native foldings, whereas

• PPV is defined as the relative frequency of correctly predicted pairs among all position pairs that were predicted to be paired with each other.

Formally, they are given by Sens. = *TP* · (*TP* + *FN*)^-1^ and PPV = *TP* · (*TP* + *FP*)^-1^, where *TP* is the number of correctly predicted base pairs (*true positives*), *FN* is the number of base pairs in the native structure that were not predicted (*false negatives*) and *FP* is the number of incorrectly predicted base pairs (*false positives*).

In order to investigate to what extend the accuracy of predicted foldings changes when different dimensions of relative disturbances are incorporated into the needed sampling probabilities, we decided to perform a series of cross-validation experiments based on the same partitions of the tRNA and 5S rRNA databases into 10 approximately equal-sized folds, respectively, as considered in [23,24]. In particular, for each sequence, we generated several sample sets on the basis of different relative error types and values, where from each of the produced samples, we derived corresponding predictions according to a number of competing reasonable selection principles and construction schemes (which can all be applied to the respective sample set without increasing the worst-case complexity of the overall algorithm).

Briefly, we employed two different well-defined selection procedures in order to identify one particular structure from the produced sample as prediction: First, we picked the most likely secondary structure (i.e., the one with the highest probability among all feasible structures for the input sequence according to the induced (L)SCFG model), in strong analogy to traditional SCFG based probabilistic structure prediction methods. This choice will be denoted by *most probable (MP)* structure subsequently. Additionally, as one of the most straightforward and reasonable choices for statistically representative samples of the overall structure ensemble, we took the most frequently sampled folding (i.e., the one with the highest number of occurrences among all candidate structures within the generated sample set), which will be named *most frequent (MF)* structure subsequently.

Note that if the samples are indeed representative with respect to the underlying ensemble distribution (i.e., if a sufficiently large number of candidate foldings is randomly generated on the basis of the corresponding conditional probability distributions considered by the employed strategy), then these two predictions should be rather identical in most cases, at least if no disturbances are considered (i.e., under the condition that the exact inside probabilities are used for deriving the respective conditional sampling distributions). In fact, any representative set of candidate structures for a given input sequence obtained by (L)SCFG based statistical sampling obviously reflects the probability distribution on all feasible foldings of that sequence which strongly depends on the corresponding inside probabilities. Thus, if the preprocessed inside values contain any errors, then the MF structure of a particular statistically representative sample set corresponds to the most likely folding of the given sequence with respect to the skewed ensemble distribution induced by the disturbed inside values, whereas the MP structure of that sample is indeed equal to the most likely folding (among all generated candidate structures) with respect to the exact ensemble distribution^g^. Hence, the results for MP and MF structure predictions might differ in the disturbed cases, especially as the gravity of generated disturbances grows.

However, we decided to additionally apply two different commonly used construction schemes for computing a new structure as predicted folding, where the predicted structure itself must not necessarily be contained in the given sample. Particularly, we first determined a *maximum expected accuracy (MEA)* structure of the generated sample set as defined in [23], which maximizes the number of correctly unpaired and paired positions with respect to the true folding and is computed on the basis of the considered sample (rather than on the basis of the entire structure ensemble for the sequence as done for example in the Pfold [3] and CONTRAfold [16] programs). Furthermore, we calculated the unique consensus structure of the produced sample, called the *centroid* structure, which effectively reflects the overall behavior of the sample set and is actually formed by all base pairs that occur in more than 50% of the sampled structures (for details, see e.g. [35]). Note that for similar reasons as discussed above for MF structure predictions, MEA and centroid structures obtained from statistically representative sample sets can only reflect the skewed ensemble distribution rather than the exact one in the disturbed case.

Last but not least, we derived two different sets of so-called *γ*_t-o_-MEA and *γ*_t-o_-centroid structures for the produced samples, respectively, as defined in [23] (in connection with sampling algorithms), where *γ*_t-o_ ∈ [0, *∞*) is a trade-off parameter for controlling the sensitivity and PPV of the predicted foldings. Note that the default choice *γ*_t-o_ = 1 serves as the neutral element with respect to the prediction, meaning the prediction is neither biased towards a better sensitivity nor to a better PPV and corresponds to the above described well-known MEA or unique centroid structure, respectively. Notably, by measuring the performance at several different settings of *γ*_t-o_ (i.e. by determining the (adjusted) sensitivity and PPV for various values of *γ*_t-o_), it becomes possible to derive a corresponding *receiver operating characteristic (ROC)* curve^h^ and to calculate the estimated *area under this curve (AUC)*, for both the MEA and the centroid prediction principle, respectively. This obviously allows for a much more informative and reliable comparison of the predictive powers of the different sampling variants than considering only the corresponding results for the default choice *γ*_t-o_ = 1.

However, the (unadjusted) sensitivity and PPV measures obtained by considering the four different (unparameterized) prediction principles sketched above are listed in Additional file 1: Tables S3a and S5a^i^, where a few selected ones are presented in Table 1. The corresponding AUC values obtained by varying instances of *γ*_t-o_ are all collected in Additional file 1: Tables S3b and S5b, some of them are presented in Table 2. Note that in accordance with [23,24], we considered any value of *γ*_t-o_ ∈ {1.25^*k*^∣ - 12 ≤ *k* ≤ - 1}∪{2^*k*^∣0 ≤ *k* ≤ 12} in order to obtain appropriate ROC curves and corresponding AUC values. Plots of some of the resulting curves can be found in Additional file 1: Figures S15 to S18 of Section Sm-II.

**Table 1:** Prediction results by means of sensitivity and PPV

				**(a)For our tRNA database**					
**Approach**	**Errors**	**MP struct.**		**MF struct.**		**MEA struct.**		**Centroid**	
		Sens.	PPV	Sens.	PPV	Sens.	PPV	Sens.	PPV
SCFG	—	0.7818	0.8437	0.7792	0.8445	0.7324	0.8939	0.6754	0.9158
	mep (0.5)	0.7822	0.8447	0.7599	0.8370	0.7169	0.8927	0.6607	0.9140
	mep (0.99)	0.7590	0.8388	0.6768	0.8004	0.6414	0.8877	0.5817	0.9127
	fep (0.5)	0.7798	0.8440	0.7234	0.8184	0.6864	0.8896	0.6292	0.9134
	fep (0.99)	0.4101	0.7295	0.2864	0.5590	0.2532	0.7776	0.2157	0.8291
LSCFG	—	0.8545	0.9534	0.8542	0.9535	0.8335	0.9736	0.8250	0.9783
	mep (0.5)	0.8545	0.9534	0.8429	0.9524	0.8236	0.9731	0.8150	0.9773
	mep (0.99)	0.8519	0.9533	0.7988	0.9413	0.7833	0.9676	0.7735	0.9726
	fep (0.5)	0.8548	0.9536	0.8224	0.9486	0.8029	0.9707	0.7940	0.9758
	fep (0.99)	0.7530	0.9325	0.5769	0.8623	0.5668	0.9075	0.5567	0.9195
				**(b) For our 5S rRNA database**					
**Approach**	**Errors**	**MP struct.**		**MF struct.**		**MEA struct.**		**Centroid**	
		Sens.	PPV	Sens.	PPV	Sens.	PPV	Sens.	PPV
SCFG	—	0.4251	0.5372	0.4251	0.5363	0.3403	0.6967	0.2689	0.8044
	mep (0.5)	0.4143	0.5280	0.4160	0.5290	0.3334	0.6987	0.2643	0.8051
	mep (0.99)	0.3897	0.5227	0.3894	0.5216	0.2957	0.7069	0.2362	0.8072
	fep (0.5)	0.4055	0.5203	0.4049	0.5198	0.3209	0.7068	0.2532	0.8087
	fep (0.99)	0.2043	0.4410	0.1756	0.3788	0.1066	0.6867	0.0814	0.7666
LSCFG	—	0.8993	0.9412	0.8997	0.9409	0.8959	0.9513	0.8873	0.9574
	mep (0.5)	0.8993	0.9412	0.8909	0.9380	0.8903	0.9478	0.8819	0.9541
	mep (0.99)	0.8989	0.9414	0.8639	0.9269	0.8659	0.9408	0.8574	0.9482
	fep (0.5)	0.8993	0.9412	0.8796	0.9328	0.8798	0.9445	0.8716	0.9515
	fep (0.99)	0.8251	0.9052	0.7162	0.8375	0.7148	0.8661	0.6986	0.8879

All values have been computed by 10-fold cross-validation procedures, using sample size 1000 and **min**_**hel **_**= min**_***HL ***_**= 1**.

**Table 2:** Prediction results by means of AUC values

	**(a) For our tRNA database**		
**Approach**	**Errors**	**MEA struct.**	**Centroid**
SCFG	—	0.828522	0.833894
	mep (0.5)	0.819658	0.823811
	mep (0.99)	0.786645	0.788478
	fep (0.5)	0.805999	0.807240
	fep (0.99)	0.440021	0.422778
LSCFG	—	0.936285	0.919736
	mep (0.5)	0.932121	0.916321
	mep (0.99)	0.916540	0.896024
	fep (0.5)	0.924191	0.908943
	fep (0.99)	0.752030	0.722737
	**(b) For our 5S rRNA database.**		
**Approach**	**Errors**	**MEA struct.**	**Centroid**
SCFG	—	0.409278	0.408549
	mep (0.5)	0.401914	0.400515
	mep (0.99)	0.376683	0.375488
	fep (0.5)	0.400827	0.397566
	fep (0.99)	0.189628	0.182902
LSCFG	—	0.914801	0.918933
	mep (0.5)	0.911963	0.915503
	mep (0.99)	0.902330	0.905126
	fep (0.5)	0.906507	0.911063
	fep (0.99)	0.776239	0.777355

All values have been computed by 10-fold cross-validation procedures, using sample size 1000 and **min**_**hel **_**= min**_***HL ***_**= 1**.

Let us first consider the results reported in Table 1. As we can see, the PPV is principally not affected by the different dimensions of disturbances caused according to mep(*prob*), as only in the case of MF structure prediction one can observe a slight change for the worse. However, with increasing value of mep, there results a moderate decline in sensitivity (with respect to all four prediction schemes) of up to about 10% for the traditional and 5% for the length-dependent sampling approach in the case of tRNAs, whereas for 5S rRNAs, the sensitivity values only decrease up to about 3% to 4% for both sampling variants. Unsurprisingly, for both RNA data, the change for the worse by means of measured sensitivity is less significant when considering MP structure predictions than when employing any of the other three principles, especially in the case of the LSCFG model. This is due to the fact that MP structures are always extracted by relying on the exact distribution (see discussion above). Altogether, these observations indicate that relative disturbances caused by mep do not have a significant negative effect on the predictive accuracy.

Moreover, Table 1 indicates that generating errors according to the fep(*prob*) variant (unsurprisingly) yields greater losses in the accuracies of selected predictions. In fact, as *prob* gets greater, there generally result considerably smaller PPV values for all four prediction schemes (mostly for MF structures) than in the corresponding undisturbed case. Furthermore, the respective sensitivity values degrade enormously, albeit again comparatively less in connection with MP structure predictions. However, these changes for the worse are obviously less significant when using the length-dependent sampling approach instead of the more general conventional variant, which matches the observations made above for disturbances caused by mep(*prob*). Nevertheless, errors produced according to fep(*prob*) for moderate percentages *prob* seem to generally have only a rather small influence on the resulting prediction accuracy. In most cases, only marginal losses in performance can be expected when disturbances are generated by fep(*prob*) with values *prob* of up to about 0.5, whereas for percentages of up to about 0.75, there should usually still result an acceptable accuracy of selected predictions (according to any of the four considered extraction principles).

Finally, it should be mentioned that all these observations and conclusions are actually affirmed by comparing the more reliable AUC results given in Table 2, which draw a rather similar picture of the behavior of both sampling approaches under the influence of the considered types and dimensions of relative disturbances in the underlying ensemble distribution.

#### Sampling quality – Specific values related to shapes

Obviously, the sensitivity and PPV measures used in the last section for assessing the accuracy of predicted foldings depend only on the numbers of correctly and incorrectly predicted base pairs (compared to the trusted database structure). For biologists, however, it is usually much more important to get the correct *shape* of the native folding. This is due to the fact that a predicted set of suboptimal foldings calculated by modern computational structure prediction methods generally contains lots of similar foldings but for biologists, only those with significant structural differences are of interest. According to these aspects, the concept of *abstract shapes* was introduced [36-38], which are defined as morphic images of secondary structures such that each shape comprises a class of analogical foldings. Notably, there are five different shape levels which have been proven to gradually increase abstraction by disregarding certain unpaired regions or combining nested helices (see e.g. [39]), where secondary structures can accordingly be considered level 0 shapes.

For these reasons, we decided to complete our analysis of the influence of disturbances to the quality of probabilistic statistical sampling by considering the following meaningful specific values related to the shapes of predictions and sampled structures as defined in [23,24]: 

• Frequency of prediction of correct structure (CSP_freq_): In how many cases is the predicted secondary structure (or its shape) equal to the correct structure (or the correct shape)?

• Frequency of correct shape occurring in a sample (CSO_freq_): In how many cases can the correct shape (on different levels) be found in the generated sample set?

• Number of occurrences of correct shape in a sample (CS_num_): How many times can the correct shape be found in the generated sample set?

• Number of different shapes in a sample (DS_num_): How many different secondary structures (or shapes) can be found in the generated sample set?

We can easily compute the respective values from the predicted structures and the corresponding sample sets that were derived for the calculation of the sensitivity and PPV measures in the last section. The obtained results are collected in Additional file 1: Tables S7a to S8g of Section Sm-II. Some of the most interesting ones are recorded in Tables 3 and 4.

**Table 3:** Comparison of sampling quality for tRNAs

		**(a) CSP**_**freq **_**values (for selection principle MP struct.)**					
**Approach**	**Errors**			**Shape level**			
		0	1	2	3	4	5
SCFG	—	0.2413	0.4082	0.5548	0.5548	0.5552	0.6278
	mep (0.5)	0.2409	0.4068	0.5548	0.5548	0.5552	0.6265
	mep (0.99)	0.1877	0.3551	0.5382	0.5382	0.5386	0.6075
	fep (0.5)	0.2339	0.4017	0.5511	0.5511	0.5516	0.6269
	fep (0.99)	0.0014	0.0384	0.1979	0.1979	0.1984	0.2326
LSCFG	—	0.3324	0.4956	0.6574	0.6574	0.6579	0.7351
	mep (0.5)	0.3324	0.4956	0.6574	0.6574	0.6579	0.7351
	mep (0.99)	0.3236	0.4892	0.6560	0.6560	0.6565	0.7332
	fep (0.5)	0.3324	0.4966	0.6588	0.6588	0.6593	0.7369
	fep (0.99)	0.0624	0.2626	0.6246	0.6250	0.6250	0.6967
		**(b) CSP**_**freq **_**values (for selection principle MF struct.)**						
**Approach**	**Errors**			**Shape level**				
		0	1	2	3	4	5	
SCFG	—	0.2099	0.3699	0.5594	0.5594	0.5599	0.6302	
	mep (0.5)	0.1683	0.3301	0.5372	0.5372	0.5377	0.6047	
	mep (0.99)	0.0522	0.1822	0.4517	0.4517	0.4517	0.5215	
	fep (0.5)	0.1049	0.2547	0.5155	0.5155	0.5160	0.5793	
	fep (0.99)	0.0000	0.0125	0.1110	0.1110	0.1119	0.2062	
LSCFG	—	0.3269	0.4892	0.6560	0.6565	0.6565	0.7337	
	mep (0.5)	0.2534	0.4235	0.6708	0.6708	0.6713	0.7485	
	mep (0.99)	0.1137	0.2954	0.6801	0.6801	0.6801	0.7568	
	fep (0.5)	0.1794	0.3653	0.6704	0.6704	0.6709	0.7531	
	fep (0.99)	0.0023	0.1262	0.6334	0.6334	0.6357	0.7240	
		**(c) CSP**_**freq **_**values (for selection principle MEA struct.)**						
**Approach**	**Errors**			**Shape level**				
		0	1	2	3	4	5	
SCFG	—	0.0555	0.2094	0.4193	0.4193	0.4207	0.4679	
	mep (0.5)	0.0416	0.1817	0.4045	0.4045	0.4055	0.4489	
	mep (0.99)	0.0125	0.0989	0.3112	0.3112	0.3126	0.3570	
	fep (0.5)	0.0245	0.1364	0.3662	0.3662	0.3666	0.4059	
	fep (0.99)	0.0000	0.0014	0.0245	0.0245	0.0250	0.0546	
LSCFG	—	0.1854	0.3574	0.4919	0.4919	0.4919	0.5465	
	mep (0.5)	0.1405	0.3056	0.4998	0.4998	0.4998	0.5567	
	mep (0.99)	0.0730	0.2191	0.4753	0.4753	0.4753	0.5284	
	fep (0.5)	0.1003	0.2556	0.4836	0.4836	0.4836	0.5409	
	fep (0.99)	0.0009	0.0781	0.3902	0.3902	0.3921	0.4508	
		**(d) CSP**_**freq **_**values (for selection principle Centroid)**						
**Approach**	**Errors**			**Shape level**				
		0	1	2	3	4	5	
SCFG	—	0.0374	0.1276	0.2973	0.2973	0.2977	0.3130	
	mep (0.5)	0.0273	0.1045	0.2779	0.2779	0.2783	0.2908	
	mep (0.99)	0.0083	0.0541	0.2007	0.2007	0.2007	0.2173	
	fep (0.5)	0.0134	0.0795	0.2473	0.2473	0.2473	0.2603	
	fep (0.99)	0.0000	0.0009	0.0120	0.0120	0.0120	0.0227	
LSCFG	—	0.1729	0.3158	0.4300	0.4300	0.4300	0.4762	
	mep (0.5)	0.1322	0.2728	0.4374	0.4374	0.4374	0.4859	
	mep (0.99)	0.0693	0.1914	0.4101	0.4101	0.4101	0.4558	
	fep (0.5)	0.0957	0.2261	0.4207	0.4207	0.4207	0.4642	
	fep (0.99)	0.0009	0.0633	0.3264	0.3264	0.3269	0.3648	

Tables record specific values related to shapes of predictions and sampled structures, obtained from our tRNA database. All results were computed by 10-fold cross-validation procedures, using sample size 1000 and **min**_**hel **_**= min**_***HL ***_**= 1**.

**Table 4:** Comparison of sampling quality for 5S rRNAs

	**(a) CSP**_**freq **_**values (for selection principle MP struct.)**						
**Approach**	**Errors**	**Shape level**					
		0	1	2	3	4	5
SCFG	—	0.0000	0.0026	0.0052	0.0131	0.0366	0.7110
	mep (0.5)	0.0000	0.0009	0.0026	0.0113	0.0287	0.7128
	mep (0.99)	0.0000	0.0026	0.0044	0.0095	0.0227	0.6919
	fep (0.5)	0.0000	0.0017	0.0043	0.0113	0.0374	0.6954
	fep (0.99)	0.0000	0.0000	0.0000	0.0017	0.0096	0.5474
LSCFG	—	0.2141	0.4256	0.4744	0.4900	0.9408	0.9843
	mep (0.5)	0.2141	0.4256	0.4744	0.4900	0.9408	0.9843
	mep (0.99)	0.1941	0.4221	0.4761	0.4892	0.9452	0.9852
	fep (0.5)	0.2124	0.4248	0.4726	0.4883	0.9417	0.9852
	fep (0.99)	0.0209	0.3029	0.3725	0.4186	0.8529	0.9809
	**(b) CSP**_**freq **_**values (for selection principle MF struct.)**						
**Approach**	**Errors**	**Shape level**					
		0	1	2	3	4	5
SCFG	—	0.0000	0.0026	0.0052	0.0131	0.0357	0.7128
	mep (0.5)	0.0000	0.0009	0.0026	0.0122	0.0305	0.7180
	mep (0.99)	0.0000	0.0026	0.0044	0.0105	0.0235	0.6902
	fep (0.5)	0.0000	0.0017	0.0043	0.0113	0.0383	0.6971
	fep (0.99)	0.0000	0.0000	0.0000	0.0035	0.0200	0.5439
LSCFG	—	0.2002	0.4256	0.4700	0.4866	0.9417	0.9861
	mep (0.5)	0.1332	0.3960	0.4439	0.4587	0.9434	0.9869
	mep (0.99)	0.0365	0.3630	0.4308	0.4491	0.9304	0.9861
	fep (0.5)	0.0801	0.3847	0.4404	0.4561	0.9400	0.9861
	fep (0.99)	0.0035	0.1497	0.2106	0.3325	0.5440	0.9730
	**(c) CSP**_**freq **_**values (for selection principle MEA struct.)**						
**Approach**	**Errors**	**Shape level**					
		0	1	2	3	4	5
SCFG	—	0.0000	0.0000	0.0000	0.0000	0.0261	0.3821
	mep (0.5)	0.0000	0.0000	0.0000	0.0000	0.0209	0.3698
	mep (0.99)	0.0000	0.0000	0.0000	0.0000	0.0122	0.3003
	fep (0.5)	0.0000	0.0000	0.0000	0.0000	0.0252	0.3438
	fep (0.99)	0.0000	0.0000	0.0000	0.0000	0.0026	0.0444
LSCFG	—	0.1062	0.3891	0.4291	0.4378	0.9051	0.9835
	mep (0.5)	0.1010	0.3751	0.4134	0.4239	0.8921	0.9782
	mep (0.99)	0.0392	0.3429	0.3986	0.4213	0.8712	0.9791
	fep (0.5)	0.0740	0.3839	0.4239	0.4387	0.8877	0.9791
	fep (0.99)	0.0017	0.1358	0.1863	0.2942	0.4970	0.9634
	**(d) CSP**_**freq **_**values (for selection principle Centroid)**						
**Approach**	**Errors**	**Shape level**					
		0	1	2	3	4	5
SCFG	—	0.0000	0.0000	0.0000	0.0000	0.0104	0.1097
	mep (0.5)	0.0000	0.0000	0.0000	0.0000	0.0104	0.1062
	mep (0.99)	0.0000	0.0000	0.0000	0.0000	0.0078	0.0827
	fep (0.5)	0.0000	0.0000	0.0000	0.0000	0.0061	0.0932
	fep (0.99)	0.0000	0.0000	0.0000	0.0000	0.0009	0.0078
LSCFG	—	0.0966	0.2916	0.3238	0.3316	0.8703	0.9686
	mep (0.5)	0.0879	0.3142	0.3516	0.3621	0.8625	0.9686
	mep (0.99)	0.0322	0.2924	0.3377	0.3595	0.8294	0.9651
	fep (0.5)	0.0662	0.3194	0.3551	0.3638	0.8512	0.9695
	fep (0.99)	0.0017	0.1053	0.1471	0.2219	0.4831	0.9339

Tables record specific values related to shapes of predictions and sampled structures, obtained from our 5S rRNA database. All results were computed by 10-fold cross-validation procedures, using sample size 1000 and **min**_**hel **_**= min**_***HL ***_**= 1**.

First, as regards tRNAs, we observe that for MP predictions, disturbances caused by mep(*prob*) do generally not have a noticeable negative impact on the frequency of correct structure predictions (see Table S7a), and for the three other extraction principles, such disturbances do at least not yield a significant decline of the corresponding CSP_freq_ value for shape levels 2 to 5 and under the assumption of the LSCFG approach, where for MF structures, there indeed results a slightly higher CSP_freq_ value with increasing relative error percentage *prob* (see Tables S7b to S7d). When the more intensive variant as defined by fep(*prob*) is used for incorporating random errors into the considered sampling probabilities, the LSCFG based sampling algorithm still yields acceptable results with respect to CSP_freq_ on abstraction levels 2 to 5, where for MP and MF structure predictions it obviously behaves quite resistant to the imposed distributions even for large values of *prob*.

Similar results are observed for 5S rRNAs (see Additional file 1: Tables S8a to S8d, where for all four prediction selecting principles, the CSP_freq_ values (for all shape levels in case of MP predictions and at least for shape levels 1 to 5 for all other prediction types) generally do not get significantly worse when applying the LSCFG sampling approach with inside values disturbed according to mep(*prob*) for any percentage *prob*∈(0,1) or according to the more intense relative disturbance variant fep(*prob*) for moderate values *prob*∈(0,1) (of up to about *prob*=0.75).

Moreover, comparing the discussed CSP_freq_ results for the LSCFG variant to the corresponding ones for the conventional SCFG approach, we get additional evidence that the length-independent sampling method reacts stronger to relative disturbances in the underlying ensemble distribution for a given sequence than its length-dependent counterpart. As already mentioned, this is due to the fact that the ensemble distribution considered in the length-dependent case is much more centered due to the more explicit (length-dependently trained) grammar parameters, such that randomly generated errors on particular probabilities carry less weight.

Now, let us consider the three remaining specific values CSO_freq_, CS_num_ and DS_num_ that can eventually be used to assess the overall quality of generated sample sets rather than the accuracy of corresponding selected predictions. Basically, the obtained CSO_freq_ and CS_num_ results for tRNAs and 5S rRNAs (as reported in Tables S7e to S7f and Tables S8e to S8f), respectively, show a similar picture and thus yield similar conclusions as the corresponding CSP_freq_ values discussed above. As a consequence to the fact that for larger relative error percentages *prob*, for the less intensive disturbance variant defined by mep(*prob*) and especially for the more grave version implied by fep(*prob*), the resulting values for CSO_freq_ and CS_num_ usually get smaller, the corresponding DS_num_ values inevitably increase with growing disturbance influences imposed by mep(*prob*) and especially fep(*prob*) (see Tables S7g and S8g). This actually means that the diversity within the generated sample sets generally gets greater as the overall sampling quality (with respect to occurrences of the correct structure in the sample) decreases, which could be fully expected.

## Conclusions

In this article, we performed a comprehensive experimental analysis on the effect of disturbances in the ensemble distribution for a given sequence to the quality of corresponding sets of candidate structures generated with the (L)SCFG based statistical sampling method studied in [23,24]. Basically, two different levels of errors were considered for randomly creating disturbances on all inside values for a given input sequence according to the underlying grammar model: relative and absolute ones.

During our analysis (on the basis of trusted sets of tRNA and 5S rRNA data), we immediately observed that even incorporating only rather small absolute errors into (all or particular instances of the) inside values causes problematic disturbances of the resulting sampling probabilities that generally lead to the generation of useless sample sets. This can be assumed to be due to the fact that the installation of absolute errors usually makes it impossible for the employed sampling strategy to identify which ones of the considered inside probabilities for a given input sequence must originally (i.e., in the exact case) have been equal or unequal to zero, which inevitably results in a misguided behavior of the strategy, as it is no longer ensured that it creates only reasonable substructures for a considered sequence fragment.

However, both SCFG approaches (length-dependent and traditional one) behave rather resistant to disturbances of the needed conditional sampling probabilities that are caused by generating (moderate) relative errors on all (and also only on particular) inside values for a given input sequence. In general, even large relative errors seem to have no enormous negative impact on both the predictive accuracy and the overall quality of generated sample sets. That is, the reaction of the (L)SCFG based statistical sampling algorithm to the relative disturbances is fair enough to still obtain meaningful structure predictions (especially if the most likely structure of the sample is selected as predicted folding, in strong analogy to conventional SCFG based DPAs), and the overall quality of the resulting sample sets is still acceptable such that they might often also be used for further applications (like, e.g. probability profiling for specific loop types).

Consequently, it seems reasonable to believe that the needed sampling probabilities do not necessarily have to be computed in the exact way, but it may probably suffice to only (adequately) approximate them. In fact, the worst-case time complexity of any particular (L)SCFG based sampling method could potentially be reduced by developing a suitable approximation procedure (or at least an adequate heuristic method) for the computation of the needed sampling probabilities, where an appropriate approximation ratio (or at least an acceptable ratio of correctly and incorrectly computed zero values) should be attempted to ensure that the sampling quality remains sufficiently high, as indicated by the experimental disturbance analysis results discussed within this article.

## Endnotes

^a^ All references starting with Sm are references to the supplementary material available at http://wwwagak.informatik.uni-kl.de/research/publications/.^b^ Note that the function max(min(*x*,1),0)= min(max(*x*, 0),1) ensures that the resulting value is still a probability, i.e. a real value from [0,1].^c^ Note that *prob*∈(0,1] is must be preliminary chosen and is then assumed to be fixed. This effectively facilitates the study of disturbances of different magnitudes.^d^ In general, longer words tend to be generated with smaller probability since we have to apply more grammar rules, each implying a factor (typically) less than 1 to the probability.^e^ If those decisions are not revised by employing backtracking procedures, see the description of the modifications incorporated into the sampling algorithm in order to deal with such situations as given in Section Resulting Modified Sampling Strategy.^f^ Note that the positive predictive value is often called *specificity*, although this measure formally obeys to a slightly different definition.^g^ This is due to the fact that the probability of a particular folding of a given RNA sequence (i.e., the probability of the corresponding derivation tree) depends only on the considered set of grammar parameters (transition and emission probabilities).^h^ Note that we here assume sensitivity as a function of PPV is an ROC curve, although correctly an ROC curve is sensitivity as a function of specificity.^i^ Note that the corresponding standard deviations on sensitivity values and PPV are recorded in Additional file 1: Tables S4 and S6; these allow for a reader to acknowledge which values are different and which ones are identical/close.

## Competing interests

Both authors declare that they have no competing interests.

## Authors’ contributions

AS developed and implemented the algorithms for generating statistical samples based on disturbed ensemble distributions. AS performed all experiments and evaluated the decline of sampling quality implied by considering the diverse kinds of disturbances. MEN supervised the work and development of ideas. AS drafted the manuscript; a revision and its final version have also been prepared by AS. Both authors have read and approved the final manuscript.

## Supplementary Material

Additional file 1 Supplementary Material. Click here for file
