# Supplementary material for: Evaluating the effect of disturbed ensemble distributions on SCFG based statistical sampling of RNA secondary structures
Source: BMC Bioinformatics. 2012 Jul 9;13:159. doi: 10.1186/1471-2105-13-159 (PMC3871765; doi:10.1186/1471-2105-13-159)
Supplement: Additional file 1 — Supplementary Material. [file 1471-2105-13-159-S1.pdf]

Supplementary Material

Sm-I Formal Description of the Sampling Process

In the sequel, given an RNA molecule r consisting of n nucleotides, we denote the corresponding sequence fragment from position i to position j, $1 \leq i \leq j \leq n$, by $R_{i,j} = r_i r_{i+1} \ldots r_{j-1} r_j$. Additionally, by $S_{i,j}$ we denote a structure on the sequence fragment $R_{i,j}$ that meets all the constraints of our definition of RNA secondary structures.

Briefly, according to [24, 28], a complete secondary structure $S_{1,n}$ for a given input sequence r of length n can be sampled in the following recursive way: Start with the entire RNA sequence $R_{1,n}$ and consecutively compute the adjacent substructures (single-stranded regions and paired substructures) of the exterior loop (from left to right). Any (paired) substructure on fragment $R_{i,j}$, $1 \leq i < j \leq n$, is folded by recursively constructing substructures (hairpins, stacked pairs, bulges, interior and multibranched loops) on smaller fragments $R_{l,h}$, $i \leq l < h \leq j$.

Note that this sampling process is similar to the traceback algorithm employed in MFE based dynamic programming algorithms. Actually, the main difference is that in those algorithms, base pairings are selected by the minimum free energy principle for the fragments $R_{i,j}$, $1 \leq i, j \leq n$ whereas here, base pairs are randomly sampled according to conditional probability distributions for the corresponding fragments. These distributions are derived from definitions of probabilities for particular choices (such as paired and unpaired bases or specific loop types). Notably, they only depend on the precomputed (skewed) inside probabilities $\widehat{\alpha}_X(i,j)$ for input sequence r, the thereof additionally precalculated probabilities

$$\widehat{\alpha}_{AT}(h,j) := \sum\nolimits_{l=(h-1)+\min_{\text{ps}}}^{(j-1)} \widehat{\alpha}_A(h,l) \cdot \widehat{\alpha}_T(l+1,j), \tag{15}$$

$$\widehat{\alpha}_{AB}(h,j) := \sum\nolimits_{l=(h-1)+\min_{\text{ps}}}^{(j-2)} \widehat{\alpha}_A(h,l) \cdot \widehat{\alpha}_B(l+1,j-1), \tag{16}$$

$$\widehat{\alpha}_{AO}(h,j) := \sum\nolimits_{l=(h-1)+\min_{\text{ps}}}^{(j-1)-\min_{\text{ps}}} \widehat{\alpha}_A(h,l) \cdot \widehat{\alpha}_O(l+1,j-1), \tag{17}$$

$$\widehat{\alpha}_{AN}(h,j) := \sum\nolimits_{l=(h-1)+\min_{\text{ps}}}^{(j-1)} \widehat{\alpha}_A(h,l) \cdot \widehat{\alpha}_N(l+1,j-1), \tag{18}$$

corresponding to inside values for combined intermediate symbols, where $i \leq h \leq j$, and of course the trained grammar parameters (transition probabilities only).

Algorithms 1 to 4 formally describe how the sampling strategy works. Note that the type (or shape) and actual composition (of accessible base pairs and unpaired bases) of a particular substructure (corresponding to a valid derivation tree) on a given fragment $R_{i,j}$ are randomly drawn according to the conditional probability distributions induced by the respective sets of all (valid) choices for the unique intermediate symbol of the grammar that generates such substructures (that represents the root of the corresponding subtree). Principally, each of the presented algorithms describing the employed sampling strategy relies on a moderate number of formal set definitions for the respective mutually exclusive and exhaustive cases in order to perform the needed random choices, which basically all obey to the same scheme.

Particularly, for sampling shape and actual composition (free base pairs and unpaired bases) of the exterior loop, Algorithm 1 considers the following sets:

$$acT(i,j) := \{\{x, prob\} \mid x \in \{C, A, CA, AT, CAT\} \text{ and } prob = \sum\nolimits_{\{y,pr\} \in acT_x(i,j)} pr \neq 0\}, \tag{19}$$

where

$$acT_C(i,j) := \{\{0, prob\} \mid prob = \widehat{\alpha}_C(i,j) \cdot \Pr_{tr}(T \to C) \neq 0\}, \tag{20}$$

$$acT_A(i,j) := \{\{0, prob\} \mid prob = \widehat{\alpha}_A(i,j) \cdot \Pr_{tr}(T \to A) \neq 0\}, \tag{21}$$

$$\begin{aligned} acT_{CA}(i,j) := \{\{h, prob\} \mid\ & (i+1) \leq h \leq (j+1) - \min_{\text{ps}} \text{ and} \\ & prob = \widehat{\alpha}_C(i,h-1) \cdot \widehat{\alpha}_A(h,j) \cdot \Pr_{tr}(T \to CA) \neq 0\}, \end{aligned} \tag{22}$$

$$\begin{aligned} acT_{AT}(i,j) := \{\{l, prob\} \mid\ & (i-1) + \min_{\text{ps}} \leq l \leq (j-1) \text{ and} \\ & prob = \widehat{\alpha}_A(i,l) \cdot \widehat{\alpha}_T(l+1,j) \cdot \Pr_{tr}(T \to AT) \neq 0\}, \end{aligned} \tag{23}$$

$$\begin{aligned} acT_{CAT}(i,j) := \{\{h, prob\} \mid\ & (i+1) \leq h \leq j - \min_{\text{ps}} \text{ and} \\ & prob = \widehat{\alpha}_C(i,h-1) \cdot \widehat{\alpha}_{AT}(h,j) \cdot \Pr_{tr}(T \to CAT) \neq 0\}, \end{aligned} \tag{24}$$

Algorithm 1 Sampling an entire secondary structure

Input: RNA sequence r of length $n \geq 1$,
trained transition probabilities $\Pr_{tr}(rule)$, for $rule \in \mathcal{R}_{\mathcal{G}_s}$,
precomputed inside probabilities $\widehat{\alpha}_X(i,j)$, for $X \in \mathcal{I}_{\mathcal{G}_s} \cup \{AT, AB, AO, AN\}$ and $1 \leq i, j \leq n$.

Output: $helices = \big\{\{i,j,k\} \mid 1 \leq i < j \leq n$ and $k \geq \min_{\text{hel}}$ and
$i.j, (i+1).(j-1), \ldots, (i+(k-1)).(j-(k-1))$ are consecutive base pairs$\big\}$.

procedure ComputeRandomExteriorLoop()
  $helices = \emptyset$
  $i = 1$, $j = n$
  while $(j - i + 1) \neq 0$ **do**
    /*Sample next substructure on $R_{i,j}$ according to $acT(i,j)$, i.e. construct paired substructure starting with free base pair $h.l$, for $i \leq h < l \leq j$, or leave $R_{i,j}$ unpaired:*/
    $extLoopType$ = Sample exterior loop substructure type for $R_{i,j}$ according to $acT(i,j)$
    if $extLoopType = C$ **then**
      /*$R_{i,j}$ becomes single-stranded:*/
      return $helices$
    else if $extLoopType = A$ **then**
      /*$R_{i,j}$ becomes paired structure:*/
      $h = i$, $l = j$
    else if $extLoopType = CA$ **then**
      /*$R_{i,j}$ becomes paired structure preceded by single-strand:*/
      Sample h according to $acT_{CA}(i,j)$
      $l = j$
    else if $extLoopType = AT$ **then**
      /*$R_{i,j}$ becomes paired structure followed by further structure(s):*/
      $h = i$
      Sample l according to $acT_{AT}(i,j)$
    else if $extLoopType = CAT$ **then**
      /*$R_{i,j}$ becomes paired structure preceded by single-strand and followed by further structure(s):*/
      Sample h according to $acT_{CAT}(i,j)$
      Sample l according to $ac^*_{AT}(h,j)$
    end if
    if $extLoopType \in \{A, CA, AT, CAT\}$ **and** $h.l$ successfully sampled **then**
      /*Recursively fold substructures on $R_{h,l}$:*/
      $helices = helices \cup \{\{h, l, \min_{\text{hel}}\}\}$
      $helices$ = ComputeRandomLoop($h + (\min_{\text{hel}} - 1), l - (\min_{\text{hel}} - 1), helices$)
      /*Consider the remaining fragment $R_{(l+1),j}$:*/
      $i = l + 1$
    else
      /*Sampling failed (as there exist no valid choices), so stop folding the loop (such that $R_{i,j}$ becomes single-stranded):*/
      return $helices$
    end if
  end while
  return $helices$
end procedure

Algorithm 2 Sampling any substructure

```
procedure ComputeRandomLoop(i, j, helices)
loopType = Sample loop type closed by i.j according to acL(i, j)
if loopType = F then
    /*Pair i.j closes hairpin loop:*/
    return helices
else if loopType = P then
    /*Pair i.j closes stacked pair:*/
    helices[−1, 3] = helices[−1, 3] + 1 /*increments length of last added helix*/
    helices = ComputeRandomLoop(i + 1, j − 1, helices)
else if loopType = G then
    /*Pair i.j closes bulge or interior loop:*/
    helices = ComputeRandomBulgeInteriorLoop(i, j, helices)
else if loopType = M then
    /*Pair i.j closes multiloop:*/
    helices = ComputeRandomMultiLoop(i, j, helices)
else
    /*Sampling failed (as there exist no valid choices), so stop folding the loop (such that R_{i+1,j−1} becomes single-stranded
    hairpin loop):*/
    return helices
end if
return helices
end procedure
```

Algorithm 3 Sampling a particular bulge or interior loop

```
procedure ComputeRandomBulgeInteriorLoop(i, j, helices)
/*Note that the following allows max_{bulge} = ∞ (then no restrictions are applied):*/
loopType = Sample bulge or interior loop type on R_{i+1,j−1} according to acG(i, j)
if loopType = BA then
    /*Bulge on the left:*/
    Sample h according to acG_{BA}(i, j)
    l = j
else if loopType = AB then
    /*Bulge on the right:*/
    h = i
    Sample l according to acG_{AB}(i, j)
else if loopType = BAB then
    /*Interior loop:*/
    Sample h according to acG_{BAB}(i, j)
    Sample l according to ac*_{AB}(h, j)
end if
if loopType ∈ {BA, AB, BAB} and h.l successfully sampled then
    /*Recursively fold substructures on R_{h,l}:*/
    helices = helices ∪ {{h, l, min_hel}}
    helices = ComputeRandomLoop(h + (min_hel − 1), l − (min_hel − 1), helices)
else
    /*Sampling failed (as there exist no valid choices), so stop folding the loop (such that R_{i+1,j−1} becomes single-stranded
    hairpin loop):*/
    return helices
end if
return helices
end procedure
```

Algorithm 4 Sampling a complete multiloop

```
procedure ComputeRandomMultiLoop(i, j, helices)
  k = 0, l_k = i
  while (j − l_k − 1) ≥ min_ps do
    /*Create (k + 1)th paired substructure on R_{l_k+1,j−1}, starting with accessible base pair h_{k+1}.l_{k+1}, for l_k < h_{k+1} < l_{k+1} < j:*/
    if (k + 1) = 1 then
      Sample h according to acM_{UAO}(l_k, j)
      Sample l according to ac*_{AO}(h, j)
    else if (k + 1) = 2 then
      Sample h according to acO_{UAN}(l_k, j)
      Sample l according to ac*_{AN}(h, j)
    else if (k + 1) ≥ 3 then
      Sample h according to acN_{UAN}(l_k, j)
      Sample l according to ac*_{AN}(h, j)
    end if
    if h.l successfully sampled then
      h_{k+1} = h, l_{k+1} = l
      /*Recursively fold substructures on R_{h_{k+1},l_{k+1}}:*/
      helices = helices ∪ {{h_{k+1}, l_{k+1}, min_hel}}
      helices = ComputeRandomLoop(h_{k+1} + (min_hel − 1), l_{k+1} − (min_hel − 1), helices)
      /*Decide whether to leave the remaining fragment R_{l_{k+1}+1,j−1} unpaired or not:*/
      if (k + 1) ≥ 2 then
        Uniformly draw real value random ∈ (0, 1]
        if random ∈ (0, dec_U(l_{k+1}, j)] then
          /*No additional base pairs:*/
          return helices
        else if random ∈ (dec_U(l_{k+1}, j), 1] then
          /*At least one more paired substructure:*/
          k = k + 1
        end if
      end if
    else
      /*Sampling failed (as there exist no valid choices), so stop folding the loop (such that R_{l_k+1,j−1} becomes single-stranded):*/
      return helices
    end if
  end while
  return helices
end procedure
```

and

$$ac^*_{AT}(h,j) := \{\{l, prob\} \mid (h-1)+\min_{\rm ps} \leq l \leq (j-1) \text{ and } prob = \widehat{\alpha}_A(h,l)\cdot\widehat{\alpha}_T(l+1,j) \neq 0\}. \quad (25)$$

For sampling the type of the loop closed by a given base pair $i.j$, Algorithm 2 relies on

$$acL(i,j) := \{\{x, prob\} \mid x \in \{F,P,G,M\} \text{ and } prob = \widehat{\alpha}_x(i+1,j-1)\cdot \Pr_{tr}(L \to x) \neq 0\}. \quad (26)$$

Algorithm 3 employs the following sets in order to sample a particular bulge or interior loop (closed by a given base pair $i.j$) on the considered sequence fragment $R_{i+1,j-1}$:

$$acG(i,j) := \{\{x, prob\} \mid x \in \{BA, AB, BAB\} \text{ and } prob = \sum\nolimits_{\{y,pr\}\in acG_x(i,j)} pr \neq 0\}, \quad (27)$$

where

$$\begin{aligned} acG_{BA}(i,j) &:= \{\{h, prob\} \mid (i+2) \leq h \leq j-\min_{\rm ps} \text{ and} \\ &\qquad prob = \widehat{\alpha}_B(i+1,h-1)\cdot\widehat{\alpha}_A(h,j-1)\cdot\Pr_{tr}(G\to BA)\neq 0\}, \quad (28)\\ acG_{AB}(i,j) &:= \{\{l, prob\} \mid i+\min_{\rm ps} \leq l \leq (j-2) \text{ and} \\ &\qquad prob = \widehat{\alpha}_A(i+1,l)\cdot\widehat{\alpha}_B(l+1,j-1)\cdot\Pr_{tr}(G\to AB)\neq 0\}, \quad (29)\\ acG_{BAB}(i,j) &:= \{\{h, prob\} \mid (i+2) \leq h \leq j-\min_{\rm ps}-1 \text{ and} \\ &\qquad prob = \widehat{\alpha}_B(i+1,h-1)\cdot\widehat{\alpha}_{AB}(h,j)\cdot\Pr_{tr}(G\to BAB)\neq 0\}, \quad (30) \end{aligned}$$

and

$$ac^*_{AB}(h,j) := \{\{h, prob\} \mid (h-1)+\min_{\rm ps} \leq l \leq (j-2) \text{ and } prob = \widehat{\alpha}_A(h,l)\cdot\widehat{\alpha}_B(l+1,j-1) \neq 0\}. \quad (31)$$

Finally, for sampling a complete multiloop (closed by a given base pair $i.j$) on the considered sequence fragment $R_{i+1,j-1}$, the following formal definitions are used by Algorithm 4:

$$\begin{aligned} acM_{UAO}(i,j) &:= \{\{h, prob\} \mid (i+1) \leq h \leq j-2\cdot\min_{\rm ps} \text{ and} \\ &\qquad prob = \widehat{\alpha}_U(i+1,h-1)\cdot\widehat{\alpha}_{AO}(h,j)\cdot\Pr_{tr}(M\to UAO)\neq 0\}, \quad (32)\\ acO_{UAN}(l_k,j) &:= \{\{h, prob\} \mid (l_k+1) \leq h \leq j-\min_{\rm ps} \text{ and} \\ &\qquad prob = \widehat{\alpha}_U(l_k+1,h-1)\cdot\widehat{\alpha}_{AN}(h,j)\cdot\Pr_{tr}(O\to UAN)\neq 0\}, \quad (33)\\ acN_{UAN}(l_k,j) &:= \{\{h, prob\} \mid (l_k+1) \leq h \leq j-\min_{\rm ps} \text{ and} \\ &\qquad prob = \widehat{\alpha}_U(l_k+1,h-1)\cdot\widehat{\alpha}_{AN}(h,j)\cdot\Pr_{tr}(N\to UAN)\neq 0\}, \quad (34) \end{aligned}$$

as well as

$$ac^*_{AO}(h,j) := \{\{l, prob\} \mid (h-1)+\min_{\rm ps} \leq l \leq (j-1)-\min_{\rm ps} \text{ and } prob = \widehat{\alpha}_A(h,l)\cdot\widehat{\alpha}_O(l+1,j-1) \neq 0\}, \quad (35)$$

$$ac^*_{AN}(h,j) := \{\{l, prob\} \mid (h-1)+\min_{\rm ps} \leq l \leq (j-1) \text{ and } prob = \widehat{\alpha}_A(h,l)\cdot\widehat{\alpha}_N(l+1,j-1) \neq 0\}, \quad (36)$$

and finally (for deciding whether an additional substructure should be added or not),

$$dec_U(l_{k+1},j) := \frac{\widehat{\alpha}_U(l_{k+1}+1,j-1)\cdot\Pr_{tr}(N\to U)}{\widehat{\alpha}_U(l_{k+1}+1,j-1)\cdot\Pr_{tr}(N\to U)+\sum_{\{h,prob\}\in acN(l_{k+1},j)} prob}. \quad (37)$$

It remains to mention that after a preprocessing of the given input sequence (including the complete dynamic programming method for deriving all inside probabilities $\widehat{\alpha}_X(i,j)$, for $X \in \mathcal{I}_{\mathcal{G}_s}$ and $1 \leq i,j \leq n$, as well as the subsequent calculation of the additionally needed probabilities $\widehat{\alpha}_x(h,j)$, for $x \in \{AT, AB, AO, AN\}$ and $1 \leq h,j \leq n$, which both take $\mathcal{O}(n^3)$ time and require $\mathcal{O}(n^2)$ storage [10]), each of the probabilities *prob* defined for a particular choice of a paired base (h or l) in the respective subset ($acX_y(i,j)$ or $ac^*_z(h,j)$) of all possible choices can be derived in constant time. Furthermore,

[10] Note that if we modify the considered SCFG $\mathcal{G}_s$ such that each occurrence of any pattern $x \in \{AT, AB, AO, AN\}$ (in the conclusions of the production rules of $\mathcal{G}_s$) is replaced by a new intermediate symbol $Y \notin \mathcal{I}_{\mathcal{G}_s}$ corresponding to the respective pattern x, then $\widehat{\alpha}_x(i,j)$, $1 \leq i,j \leq n$, is equal to the inside probability $\widehat{\alpha}_Y(i,j)$ of this new intermediate symbol Y and is automatically derived during the inside value computations.

according to their definitions, none of these subsets contains more than n choices for a particular paired base in the worst-case, that is $\texttt{card}(acX_y(i,j)) \in \mathcal{O}(n)$ and $\texttt{card}(ac^*_z(h,j)) \in \mathcal{O}(n)$. Hence, the sampling strategy needs $\mathcal{O}(n)$ time for deriving the respective probability distribution and drawing a corresponding random choice.

Additionally, due to the cardinalities of $\mathcal{O}(n)$ for each of the $\mathcal{O}(1)$ distinct subsets $acX_y(i,j)$ (corresponding to production rules $X \to y$) for any main set $acX(i,j)$ (for premise X), each of the probabilities defined for a particular choice of the shape of a random substructure (corresponding to one of its subsets and hence to the respective production $X \to y$ applied from the considered intermediate symbol X in order to generate that shape) can be computed in $\mathcal{O}(n)$ time (since for each of the $\mathcal{O}(1)$ rules $X \to y$, we have to compute the sum of $\texttt{card}(acX_y(i,j)) \in \mathcal{O}(n)$ terms, where each term is obtained in constant time, see above). Then, the respective probability distribution employed for (shape or loop type) sampling can be derived in constant time (as $\texttt{card}(acX(i,j)) \in \mathcal{O}(1)$). For example, the distribution for sampling the exterior loop substructure type according to $acT(i,j)$ can be derived in $2 \cdot \mathcal{O}(1) + 3 \cdot \mathcal{O}(n)$ time.

Altogether, there obviously results $\mathcal{O}(n)$ time complexity for sampling a random base pair $h.l$ (by first sampling the substructure type (if needed), then the leftmost base h and finally the rightmost base l) on $R_{i,j}$, $1 \leq i \leq h < l \leq j \leq n$. Thus, since any structure of size n can have at most $\lfloor \frac{n-\min_{HL}}{2} \rfloor \in \mathcal{O}(n)$ base pairs and any base pair can be sampled in linear time, the time requirements of the sampling strategy for constructing a complete secondary structure $S_{1,n}$ is bounded by $\mathcal{O}(n^2)$.

Sm-II Tables and Figures

X	card($\mathcal{X}^e$)	card($\mathcal{X}^d$)		card($\mathcal{X}^e \cap \mathcal{X}^d$)		card($\mathcal{X}^e \setminus \mathcal{X}^d$)		card($\mathcal{X}^d \setminus \mathcal{X}^e$)	
		mev	fev	mev	fev	mev	fev	mev	fev
A	2649	1733	1698	1547	1529	1102	1120	186	169
B	2926	1704	1741	1660	1709	1266	1217	44	32
C	2926	1847	1847	1806	1808	1120	1118	41	39
F	2926	1873	1891	1840	1849	1086	1077	33	42
G	2696	1777	1781	1616	1617	1080	1079	161	164
M	2548	1597	1573	1357	1338	1191	1210	240	235
N	3002	1957	1945	1957	1945	1045	1057	0	0
O	2770	1838	1818	1727	1692	1043	1078	111	126
P	2649	1721	1745	1553	1563	1096	1086	168	182
T	2926	1865	1905	1822	1869	1104	1057	43	36
U	3002	1938	1913	1938	1913	1064	1089	0	0
AT	2697	2699	2698	2697	2697	0	0	2	1
AB	2552	2554	2553	2552	2552	0	0	2	1
AO	2478	2482	2481	2478	2478	0	0	4	3
AN	2697	2699	2698	2697	2697	0	0	2	1

(a) Traditional SCFG model.

X	card($\mathcal{X}^e$)	card($\mathcal{X}^d$)		card($\mathcal{X}^e \cap \mathcal{X}^d$)		card($\mathcal{X}^e \setminus \mathcal{X}^d$)		card($\mathcal{X}^d \setminus \mathcal{X}^e$)	
		mev	fev	mev	fev	mev	fev	mev	fev
A	469	1587	1630	325	326	144	143	1262	1304
B	1651	1996	1980	1337	1310	314	341	659	670
C	1096	2001	1987	1053	1025	43	71	948	962
F	871	1888	1850	819	801	52	70	1069	1049
G	729	1603	1583	457	457	272	272	1146	1126
M	359	1517	1525	170	184	189	175	1347	1341
N	1331	1601	1626	786	758	545	573	815	868
O	690	1524	1527	357	355	333	335	1167	1172
P	435	1612	1565	312	306	123	129	1300	1259
T	708	1772	1752	614	594	94	114	1158	1158
U	1571	2038	2059	1323	1322	248	249	715	737
AT	1394	2630	2613	1394	1394	0	0	1236	1219
AB	1829	2485	2469	1829	1829	0	0	656	640
AO	499	2308	2291	499	499	0	0	1809	1792
AN	1832	2620	2602	1831	1832	1	0	789	770

(b) LSCFG model.

Table S1: **Comparison of relevant inside probabilities.** Tabulated values are the numbers of relevant inside probabilities (being greater than zero) that were considered for obtaining the profiles presented in Figure 2b (and Figure S2), where $\mathcal{X}^e := \{\{i,j\} \mid 1 \leq i,j \leq n \text{ and } \alpha_X(i,j) \neq 0\}$ and $\mathcal{X}^d := \{\{i,j\} \mid 1 \leq i,j \leq n \text{ and } \widehat{\alpha}_X(i,j) \neq 0\}$.

$\mathcal{X}$	$\texttt{card}(\mathcal{X}^e)$	$\texttt{card}(\mathcal{X}^d)$		$\texttt{card}(\mathcal{X}^e \cap \mathcal{X}^d)$		$\texttt{card}(\mathcal{X}^e \setminus \mathcal{X}^d)$		$\texttt{card}(\mathcal{X}^d \setminus \mathcal{X}^e)$	
		mev	fev	mev	fev	mev	fev	mev	fev
$\mathcal{T}_C$	76	36	39	36	39	40	37	0	0
$\mathcal{T}_A$	54	33	41	22	32	32	22	11	9
$\mathcal{T}_{CA}$	1829	766	856	480	673	1349	1156	286	183
$\mathcal{T}_{AT}$	2595	999	961	966	924	1629	1671	33	37
$\mathcal{T}_{CAT}$	2628	1644	1646	1644	1646	984	982	0	0
$\mathcal{AT}$	62102	25675	25693	24940	24671	37162	37431	735	1022
$\mathcal{L}_F$	2775	1750	1777	1750	1777	1025	998	0	0
$\mathcal{L}_P$	2522	1533	1542	1487	1486	1035	1036	46	56
$\mathcal{L}_G$	2552	1543	1539	1540	1536	1012	1016	3	3
$\mathcal{L}_M$	2408	1290	1265	1288	1261	1120	1147	2	4
$\mathcal{G}_{BA}$	59580	23057	23288	22390	22547	37190	37033	667	741
$\mathcal{G}_{AB}$	0	0	0	0	0	0	0	0	0
$\mathcal{G}_{BAB}$	59476	36453	37479	36428	37461	23048	22015	25	18
$\mathcal{AB}$	1041908	454132	457662	441016	442856	600892	599052	13116	14806
$\mathcal{M}_{UAO}$	56901	41296	40054	41208	40010	15693	16891	88	44
$\mathcal{AO}$	980735	488660	456896	473742	442002	506993	538733	14918	14894
$\mathcal{O}_{UAN}$	56999	41352	40087	41329	40066	15670	16933	23	21
$\mathcal{N}_{UAN}$	49970	36636	35377	36615	35356	13355	14614	21	21
$\mathcal{AN}$	985172	511715	490277	497364	474716	487808	510456	14351	15561

(a) Traditional SCFG model.

$\mathcal{X}$	$\texttt{card}(\mathcal{X}^e)$	$\texttt{card}(\mathcal{X}^d)$		$\texttt{card}(\mathcal{X}^e \cap \mathcal{X}^d)$		$\texttt{card}(\mathcal{X}^e \setminus \mathcal{X}^d)$		$\texttt{card}(\mathcal{X}^d \setminus \mathcal{X}^e)$	
		mev	fev	mev	fev	mev	fev	mev	fev
$\mathcal{T}_C$	7	7	7	7	7	0	0	0	0
$\mathcal{T}_A$	1	3	1	0	0	1	1	3	1
$\mathcal{T}_{CA}$	33	280	198	13	7	20	26	267	191
$\mathcal{T}_{AT}$	55	256	298	33	25	22	30	223	273
$\mathcal{T}_{CAT}$	161	477	461	157	153	4	8	320	308
$\mathcal{AT}$	2936	17032	26734	1916	1870	1020	1066	15116	24864
$\mathcal{L}_F$	845	795	780	795	780	50	65	0	0
$\mathcal{L}_P$	409	603	581	295	292	114	117	308	289
$\mathcal{L}_G$	669	431	429	428	423	241	246	3	6
$\mathcal{L}_M$	308	152	162	152	162	156	146	0	0
$\mathcal{G}_{BA}$	401	844	881	351	355	50	46	493	526
$\mathcal{G}_{AB}$	0	0	0	0	0	0	0	0	0
$\mathcal{G}_{BAB}$	5074	11964	11884	4002	3916	1072	1158	7962	7968
$\mathcal{AB}$	173376	457279	487255	109429	110855	63947	62521	347850	376400
$\mathcal{M}_{UAO}$	4229	10068	10201	3926	3939	303	290	6142	6262
$\mathcal{AO}$	19149	279648	298214	8090	8203	11059	10946	271558	290011
$\mathcal{O}_{UAN}$	11284	16633	16787	9773	9863	1511	1421	6860	6924
$\mathcal{N}_{UAN}$	11880	18444	18496	10324	10491	1556	1389	8120	8005
$\mathcal{AN}$	89494	306125	329250	45081	44257	44413	45237	261044	284993

(b) LSCFG model.

Table S2: **Comparison of relevant sampling probabilities.** Tabulated values are the numbers of relevant sampling probabilities (being greater than zero) that were considered for obtaining the profiles presented in Figure 2b (and Figure S2), where $\mathcal{X}_y^z := \bigcup_{1 \leq i,j \leq n} acX_y(i,j)$ and $\mathcal{Y}^z := \bigcup_{1 \leq i \leq h \leq j \leq n} ac_Y^*(h,j)$, with $z = e$ and $z = d$ denoting the exact and disturbed values, respectively.

Approach	Errors	MP struct.		MF struct.		MEA struct.		Centroid	
		Sens.	PPV	Sens.	PPV	Sens.	PPV	Sens.	PPV
SCFG	—	0.7818	0.8437	0.7792	0.8445	0.7324	0.8939	0.6754	0.9158
	mep(0.5)	0.7822	0.8447	0.7599	0.8370	0.7169	0.8927	0.6607	0.9140
	mep(0.75)	0.7793	0.8431	0.7303	0.8217	0.6935	0.8917	0.6356	0.9123
	mep(0.9)	0.7699	0.8409	0.7075	0.8117	0.6715	0.8893	0.6097	0.9115
	mep(0.99)	0.7590	0.8388	0.6768	0.8004	0.6414	0.8877	0.5817	0.9127
	fep(0.5)	0.7798	0.8440	0.7234	0.8184	0.6864	0.8896	0.6292	0.9134
	fep(0.75)	0.7442	0.8313	0.6414	0.7736	0.6066	0.8802	0.5507	0.9032
	fep(0.9)	0.6644	0.8106	0.5257	0.7229	0.4934	0.8652	0.4375	0.8952
	fep(0.99)	0.4101	0.7295	0.2864	0.5590	0.2532	0.7776	0.2157	0.8291
LSCFG	—	0.8545	0.9534	0.8542	0.9535	0.8335	0.9736	0.8250	0.9783
	mep(0.5)	0.8545	0.9534	0.8429	0.9524	0.8236	0.9731	0.8150	0.9773
	mep(0.75)	0.8542	0.9533	0.8281	0.9485	0.8098	0.9709	0.8018	0.9758
	mep(0.9)	0.8546	0.9539	0.8104	0.9425	0.7978	0.9697	0.7889	0.9744
	mep(0.99)	0.8519	0.9533	0.7988	0.9413	0.7833	0.9676	0.7735	0.9726
	fep(0.5)	0.8548	0.9536	0.8224	0.9486	0.8029	0.9707	0.7940	0.9758
	fep(0.75)	0.8524	0.9532	0.7763	0.9323	0.7674	0.9620	0.7589	0.9687
	fep(0.9)	0.8315	0.9492	0.7223	0.9162	0.7131	0.9523	0.7038	0.9601
	fep(0.99)	0.7530	0.9325	0.5769	0.8623	0.5668	0.9075	0.5567	0.9195

(a) Sensitivity and PPV.

Approach	Errors	MEA struct.	Centroid
SCFG	—	0.828522	0.833894
	mep(0.5)	0.819658	0.823811
	mep(0.75)	0.810331	0.813818
	mep(0.9)	0.801393	0.801842
	mep(0.99)	0.786645	0.788478
	fep(0.5)	0.805999	0.807240
	fep(0.75)	0.761806	0.759493
	fep(0.9)	0.682057	0.676879
	fep(0.99)	0.440021	0.422778
LSCFG	—	0.936285	0.919736
	mep(0.5)	0.932121	0.916321
	mep(0.75)	0.925639	0.907926
	mep(0.9)	0.919747	0.900505
	mep(0.99)	0.916540	0.896024
	fep(0.5)	0.924191	0.908943
	fep(0.75)	0.900592	0.884400
	fep(0.9)	0.872742	0.848190
	fep(0.99)	0.752030	0.722737

(b) AUC values.

Table S3: **Prediction results for our tRNA database.** They have been computed by 10-fold cross-validation procedures, using sample size 1000 and $\min_{\text{hel}} = \min_{HL} = 1$.

Approach	Errors	MP struct.		MF struct.		MEA struct.		Centroid	
		Sens.	PPV	Sens.	PPV	Sens.	PPV	Sens.	PPV
SCFG	—	0.2631	0.1887	0.2580	0.1850	0.1935	0.1430	0.2068	0.1313
	mep(0.5)	0.2622	0.1870	0.2597	0.1892	0.1957	0.1450	0.2064	0.1332
	mep(0.75)	0.2621	0.1876	0.2598	0.1969	0.1933	0.1475	0.2035	0.1391
	mep(0.9)	0.2627	0.1884	0.2577	0.2019	0.1992	0.1552	0.2060	0.1445
	mep(0.99)	0.2636	0.1886	0.2591	0.2057	0.2012	0.1621	0.2093	0.1453
	fep(0.5)	0.2628	0.1875	0.2589	0.1978	0.1991	0.1497	0.2077	0.1387
	fep(0.75)	0.2664	0.1941	0.2568	0.2204	0.1972	0.1639	0.2035	0.1553
	fep(0.9)	0.2702	0.2086	0.2459	0.2587	0.2052	0.2143	0.2047	0.2023
	fep(0.99)	0.2456	0.3097	0.2103	0.3327	0.1814	0.3519	0.1741	0.3415
LSCFG	—	0.1657	0.0895	0.1647	0.0869	0.1541	0.0706	0.1566	0.0635
	mep(0.5)	0.1657	0.0895	0.1650	0.0867	0.1519	0.0704	0.1545	0.0655
	mep(0.75)	0.1666	0.0908	0.1649	0.0913	0.1529	0.0727	0.1559	0.0662
	mep(0.9)	0.1656	0.0890	0.1695	0.0977	0.1554	0.0744	0.1582	0.0692
	mep(0.99)	0.1664	0.0903	0.1668	0.0970	0.1581	0.0785	0.1622	0.0742
	fep(0.5)	0.1655	0.0894	0.1674	0.0939	0.1562	0.0766	0.1591	0.0703
	fep(0.75)	0.1667	0.0896	0.1669	0.1013	0.1572	0.0831	0.1597	0.0791
	fep(0.9)	0.1688	0.0908	0.1764	0.1197	0.1682	0.0946	0.1717	0.0910
	fep(0.99)	0.1764	0.1004	0.1969	0.1803	0.1880	0.1636	0.1911	0.1571

Table S4: **Standard deviations on sensitivity values and PPV for our tRNA database.** Presented results correspond to those presented in Table S3a.

Approach	Errors	MP struct.		MF struct.		MEA struct.		Centroid	
		Sens.	PPV	Sens.	PPV	Sens.	PPV	Sens.	PPV
SCFG	—	0.4251	0.5372	0.4251	0.5363	0.3403	0.6967	0.2689	0.8044
	mep(0.5)	0.4143	0.5280	0.4160	0.5290	0.3334	0.6987	0.2643	0.8051
	mep(0.75)	0.4113	0.5303	0.4105	0.5289	0.3234	0.7031	0.2566	0.8098
	mep(0.9)	0.4071	0.5311	0.4064	0.5297	0.3120	0.7007	0.2466	0.8050
	mep(0.99)	0.3897	0.5227	0.3894	0.5216	0.2957	0.7069	0.2362	0.8072
	fep(0.5)	0.4055	0.5203	0.4049	0.5198	0.3209	0.7068	0.2532	0.8087
	fep(0.75)	0.3713	0.5070	0.3708	0.5050	0.2795	0.7121	0.2247	0.8183
	fep(0.9)	0.3321	0.4953	0.3261	0.4858	0.2296	0.7344	0.1829	0.8161
	fep(0.99)	0.2043	0.4410	0.1756	0.3788	0.1066	0.6867	0.0814	0.7666
LSCFG	—	0.8993	0.9412	0.8997	0.9409	0.8959	0.9513	0.8873	0.9574
	mep(0.5)	0.8993	0.9412	0.8909	0.9380	0.8903	0.9478	0.8819	0.9541
	mep(0.75)	0.8993	0.9411	0.8816	0.9348	0.8822	0.9459	0.8746	0.9528
	mep(0.9)	0.8993	0.9414	0.8745	0.9323	0.8739	0.9438	0.8666	0.9500
	mep(0.99)	0.8989	0.9414	0.8639	0.9269	0.8659	0.9408	0.8574	0.9482
	fep(0.5)	0.8993	0.9412	0.8796	0.9328	0.8798	0.9445	0.8716	0.9515
	fep(0.75)	0.8963	0.9400	0.8548	0.9217	0.8560	0.9346	0.8480	0.9432
	fep(0.9)	0.8854	0.9353	0.8240	0.9065	0.8260	0.9234	0.8170	0.9338
	fep(0.99)	0.8251	0.9052	0.7162	0.8375	0.7148	0.8661	0.6986	0.8879

(a) Sensitivity and PPV.

Approach	Errors	MEA struct.	Centroid
SCFG	—	0.409278	0.408549
	mep(0.5)	0.401914	0.400515
	mep(0.75)	0.397622	0.396770
	mep(0.9)	0.383750	0.383935
	mep(0.99)	0.376683	0.375488
	fep(0.5)	0.400827	0.397566
	fep(0.75)	0.363824	0.363257
	fep(0.9)	0.326873	0.325467
	fep(0.99)	0.189628	0.182902
LSCFG	—	0.914801	0.918933
	mep(0.5)	0.911963	0.915503
	mep(0.75)	0.908958	0.911579
	mep(0.9)	0.905646	0.908203
	mep(0.99)	0.902330	0.905126
	fep(0.5)	0.906507	0.911063
	fep(0.75)	0.893417	0.895371
	fep(0.9)	0.875529	0.877256
	fep(0.99)	0.776239	0.777355

(b) AUC values.

Table S5: **Prediction results for our 5S rRNA database.** They have been computed by 10-fold cross-validation procedures, using sample size 1000 and $\min_{\text{hel}} = \min_{HL} = 1$.

Approach	Errors	MP struct.		MF struct.		MEA struct.		Centroid	
		Sens.	PPV	Sens.	PPV	Sens.	PPV	Sens.	PPV
SCFG	—	0.2125	0.2606	0.2123	0.2599	0.1451	0.2332	0.1164	0.2049
	mep(0.5)	0.2061	0.2543	0.2055	0.2533	0.1420	0.2370	0.1153	0.2075
	mep(0.75)	0.2071	0.2566	0.2072	0.2559	0.1374	0.2348	0.1118	0.2127
	mep(0.9)	0.2013	0.2569	0.2015	0.2567	0.1347	0.2354	0.1135	0.2209
	mep(0.99)	0.1931	0.2545	0.1928	0.2527	0.1307	0.2404	0.1065	0.2225
	fep(0.5)	0.2050	0.2569	0.2043	0.2552	0.1356	0.2333	0.1112	0.2098
	fep(0.75)	0.1880	0.2575	0.1877	0.2559	0.1228	0.2421	0.1015	0.2155
	fep(0.9)	0.1761	0.2570	0.1743	0.2544	0.1128	0.2652	0.1017	0.2504
	fep(0.99)	0.1420	0.2812	0.1300	0.2584	0.0862	0.3650	0.0790	0.3562
LSCFG	—	0.1260	0.0993	0.1249	0.0993	0.1174	0.0840	0.1210	0.0779
	mep(0.5)	0.1261	0.0991	0.1231	0.0960	0.1193	0.0855	0.1229	0.0793
	mep(0.75)	0.1259	0.0991	0.1241	0.0984	0.1216	0.0858	0.1256	0.0802
	mep(0.9)	0.1257	0.0986	0.1208	0.0981	0.1185	0.0870	0.1220	0.0824
	mep(0.99)	0.1252	0.0986	0.1238	0.1009	0.1200	0.0850	0.1253	0.0795
	fep(0.5)	0.1259	0.0994	0.1206	0.0993	0.1197	0.0875	0.1243	0.0824
	fep(0.75)	0.1246	0.0983	0.1266	0.1052	0.1226	0.0908	0.1266	0.0854
	fep(0.9)	0.1252	0.0999	0.1333	0.1111	0.1272	0.0931	0.1321	0.0874
	fep(0.99)	0.1381	0.1126	0.1613	0.1424	0.1570	0.1238	0.1632	0.1145

Table S6: **Standard deviations on sensitivity values and PPV for our 5S rRNA database.** Presented results correspond to those presented in Table S5a.

Approach	Errors	Shape Level					
		0	1	2	3	4	5
SCFG	—	0.2413	0.4082	0.5548	0.5548	0.5552	0.6278
	mep(0.5)	0.2409	0.4068	0.5548	0.5548	0.5552	0.6265
	mep(0.75)	0.2335	0.3990	0.5506	0.5506	0.5511	0.6246
	mep(0.9)	0.2159	0.3809	0.5446	0.5446	0.5451	0.6135
	mep(0.99)	0.1877	0.3551	0.5382	0.5382	0.5386	0.6075
	fep(0.5)	0.2339	0.4017	0.5511	0.5511	0.5516	0.6269
	fep(0.75)	0.1586	0.3269	0.5257	0.5257	0.5261	0.5908
	fep(0.9)	0.0564	0.1979	0.4401	0.4401	0.4401	0.4952
	fep(0.99)	0.0014	0.0384	0.1979	0.1979	0.1984	0.2326
LSCFG	—	0.3324	0.4956	0.6574	0.6574	0.6579	0.7351
	mep(0.5)	0.3324	0.4956	0.6574	0.6574	0.6579	0.7351
	mep(0.75)	0.3329	0.4952	0.6579	0.6579	0.6584	0.7351
	mep(0.9)	0.3315	0.4901	0.6574	0.6574	0.6579	0.7351
	mep(0.99)	0.3236	0.4892	0.6560	0.6560	0.6565	0.7332
	fep(0.5)	0.3324	0.4966	0.6588	0.6588	0.6593	0.7369
	fep(0.75)	0.3232	0.4827	0.6551	0.6551	0.6556	0.7341
	fep(0.9)	0.2358	0.4055	0.6394	0.6399	0.6399	0.7166
	fep(0.99)	0.0624	0.2626	0.6246	0.6250	0.6250	0.6967

(a) CSP_{freq} values (for selection principle MP struct.).

Approach	Errors	Shape Level					
		0	1	2	3	4	5
SCFG	—	0.2099	0.3699	0.5594	0.5594	0.5599	0.6302
	mep(0.5)	0.1683	0.3301	0.5372	0.5372	0.5377	0.6047
	mep(0.75)	0.1128	0.2700	0.5062	0.5062	0.5067	0.5682
	mep(0.9)	0.0712	0.2173	0.4808	0.4808	0.4813	0.5511
	mep(0.99)	0.0522	0.1822	0.4517	0.4517	0.4517	0.5215
	fep(0.5)	0.1049	0.2547	0.5155	0.5155	0.5160	0.5793
	fep(0.75)	0.0231	0.1317	0.4087	0.4087	0.4092	0.4623
	fep(0.9)	0.0032	0.0518	0.2918	0.2918	0.2918	0.3505
	fep(0.99)	0.0000	0.0125	0.1110	0.1110	0.1119	0.2062
LSCFG	—	0.3269	0.4892	0.6560	0.6565	0.6565	0.7337
	mep(0.5)	0.2534	0.4235	0.6708	0.6708	0.6713	0.7485
	mep(0.75)	0.1872	0.3666	0.6741	0.6741	0.6745	0.7550
	mep(0.9)	0.1502	0.3384	0.6694	0.6694	0.6699	0.7545
	mep(0.99)	0.1137	0.2954	0.6801	0.6801	0.6801	0.7568
	fep(0.5)	0.1794	0.3653	0.6704	0.6704	0.6709	0.7531
	fep(0.75)	0.0726	0.2492	0.6708	0.6717	0.6713	0.7596
	fep(0.9)	0.0301	0.1933	0.6847	0.6852	0.6857	0.7688
	fep(0.99)	0.0023	0.1262	0.6334	0.6334	0.6357	0.7240

(b) CSP_{freq} values (for selection principle MF struct.).

Approach	Errors	Shape Level					
		0	1	2	3	4	5
SCFG	—	0.0555	0.2094	0.4193	0.4193	0.4207	0.4679
	mep(0.5)	0.0416	0.1817	0.4045	0.4045	0.4055	0.4489
	mep(0.75)	0.0222	0.1456	0.3694	0.3699	0.3703	0.4147
	mep(0.9)	0.0148	0.1179	0.3555	0.3560	0.3583	0.4031
	mep(0.99)	0.0125	0.0989	0.3112	0.3112	0.3126	0.3570
	fep(0.5)	0.0245	0.1364	0.3662	0.3662	0.3666	0.4059
	fep(0.75)	0.0069	0.0712	0.2682	0.2686	0.2705	0.3070
	fep(0.9)	0.0005	0.0240	0.1655	0.1655	0.1669	0.2006
	fep(0.99)	0.0000	0.0014	0.0245	0.0245	0.0250	0.0546
LSCFG	—	0.1854	0.3574	0.4919	0.4919	0.4919	0.5465
	mep(0.5)	0.1405	0.3056	0.4998	0.4998	0.4998	0.5567
	mep(0.75)	0.1128	0.2760	0.4864	0.4873	0.4864	0.5432
	mep(0.9)	0.0924	0.2478	0.4827	0.4827	0.4827	0.5377
	mep(0.99)	0.0730	0.2191	0.4753	0.4753	0.4753	0.5284
	fep(0.5)	0.1003	0.2556	0.4836	0.4836	0.4836	0.5409
	fep(0.75)	0.0532	0.2011	0.4771	0.4776	0.4771	0.5423
	fep(0.9)	0.0213	0.1341	0.4508	0.4517	0.4508	0.5095
	fep(0.99)	0.0009	0.0781	0.3902	0.3902	0.3921	0.4508

(c) $\mathrm{CSP}_{\mathrm{freq}}$ values (for selection principle MEA struct.).

Approach	Errors	Shape Level					
		0	1	2	3	4	5
SCFG	—	0.0374	0.1276	0.2973	0.2973	0.2977	0.3130
	mep(0.5)	0.0273	0.1045	0.2779	0.2779	0.2783	0.2908
	mep(0.75)	0.0139	0.0716	0.2362	0.2362	0.2362	0.2520
	mep(0.9)	0.0074	0.0656	0.2354	0.2354	0.2354	0.2502
	mep(0.99)	0.0083	0.0541	0.2007	0.2007	0.2007	0.2173
	fep(0.5)	0.0134	0.0795	0.2473	0.2473	0.2473	0.2603
	fep(0.75)	0.0037	0.0360	0.1609	0.1609	0.1609	0.1734
	fep(0.9)	0.0000	0.0069	0.0865	0.0865	0.0869	0.0939
	fep(0.99)	0.0000	0.0009	0.0120	0.0120	0.0120	0.0227
LSCFG	—	0.1729	0.3158	0.4300	0.4300	0.4300	0.4762
	mep(0.5)	0.1322	0.2728	0.4374	0.4374	0.4374	0.4859
	mep(0.75)	0.1100	0.2469	0.4258	0.4258	0.4258	0.4748
	mep(0.9)	0.0874	0.2140	0.4189	0.4189	0.4189	0.4660
	mep(0.99)	0.0693	0.1914	0.4101	0.4101	0.4101	0.4558
	fep(0.5)	0.0957	0.2261	0.4207	0.4207	0.4207	0.4642
	fep(0.75)	0.0481	0.1688	0.4046	0.4046	0.4046	0.4559
	fep(0.9)	0.0199	0.1146	0.3828	0.3833	0.3828	0.4262
	fep(0.99)	0.0009	0.0633	0.3264	0.3264	0.3269	0.3648

(d) $\mathrm{CSP}_{\mathrm{freq}}$ values (for selection principle Centroid).

Approach	Errors	Shape Level					
		0	1	2	3	4	5
SCFG	—	0.6838	0.9459	0.9903	0.9903	0.9908	0.9995
	mep(0.5)	0.6274	0.9376	0.9880	0.9884	0.9889	0.9995
	mep(0.75)	0.5724	0.9274	0.9898	0.9908	0.9908	1.0000
	mep(0.9)	0.4707	0.9219	0.9866	0.9871	0.9875	1.0000
	mep(0.99)	0.3837	0.9057	0.9898	0.9903	0.9908	0.9995
	fep(0.5)	0.5534	0.9293	0.9884	0.9889	0.9889	0.9995
	fep(0.75)	0.2903	0.8849	0.9852	0.9857	0.9861	0.9995
	fep(0.9)	0.0883	0.8077	0.9838	0.9843	0.9843	0.9995
	fep(0.99)	0.0018	0.4808	0.9556	0.9575	0.9603	0.9931
LSCFG	—	0.8234	0.9288	0.9723	0.9750	0.9727	0.9986
	mep(0.5)	0.8169	0.9311	0.9658	0.9681	0.9663	0.9986
	mep(0.75)	0.7827	0.9260	0.9732	0.9760	0.9737	0.9986
	mep(0.9)	0.7291	0.9191	0.9718	0.9732	0.9723	0.9986
	mep(0.99)	0.6653	0.9122	0.9709	0.9746	0.9713	0.9986
	fep(0.5)	0.7735	0.9173	0.9704	0.9741	0.9709	0.9986
	fep(0.75)	0.6191	0.9048	0.9686	0.9713	0.9690	0.9986
	fep(0.9)	0.3777	0.8604	0.9732	0.9750	0.9736	0.9986
	fep(0.99)	0.0763	0.7106	0.9663	0.9686	0.9667	0.9986

(e) CSO_{freq} values.

Approach	Errors	Shape Level					
		0	1	2	3	4	5
SCFG	—	16.202	98.357	327.26	327.27	327.51	418.80
	mep(0.5)	13.511	90.408	314.52	314.53	314.83	405.33
	mep(0.75)	9.9097	77.641	295.10	295.12	295.47	387.67
	mep(0.9)	7.1723	66.885	278.33	278.35	278.81	373.45
	mep(0.99)	5.2356	56.709	255.51	255.54	256.20	354.90
	fep(0.5)	9.7356	77.552	294.89	294.91	295.31	387.38
	fep(0.75)	3.0058	48.215	239.50	239.53	240.34	333.77
	fep(0.9)	0.5193	22.122	171.61	171.72	173.31	270.84
	fep(0.99)	0.0028	5.3030	62.460	62.587	66.606	168.64
LSCFG	—	101.69	326.26	708.52	708.94	709.42	805.87
	mep(0.5)	90.408	307.25	712.29	712.73	713.23	810.52
	mep(0.75)	75.220	288.57	710.49	710.94	711.54	810.28
	mep(0.9)	62.276	270.87	708.82	709.20	709.93	809.72
	mep(0.99)	51.262	252.51	708.00	708.35	709.20	807.00
	fep(0.5)	70.493	281.00	710.79	711.18	711.75	810.24
	fep(0.75)	40.142	229.20	704.79	705.33	706.27	807.32
	fep(0.9)	20.373	193.27	695.77	696.29	698.20	802.86
	fep(0.99)	1.6771	118.55	612.73	612.89	620.77	752.68

(f) CS_{num} values.

Approach	Errors	Shape Level					
		0	1	2	3	4	5
SCFG	—	802.27	244.52	60.504	60.030	59.916	28.764
	mep(0.5)	805.91	250.25	63.247	62.668	62.520	29.780
	mep(0.75)	812.80	259.32	67.778	67.072	66.880	31.417
	mep(0.9)	812.01	267.01	72.572	71.611	71.323	32.755
	mep(0.99)	822.99	285.70	81.360	80.006	79.564	35.462
	fep(0.5)	813.01	261.18	68.849	68.053	67.839	31.848
	fep(0.75)	820.06	289.39	86.049	84.434	83.880	37.363
	fep(0.9)	823.37	338.60	120.77	116.73	114.89	47.031
	fep(0.99)	787.70	437.67	225.13	209.08	198.18	68.691
LSCFG	—	238.30	15.045	5.6854	5.4122	5.1806	3.2274
	mep(0.5)	237.12	15.061	5.7478	5.4543	5.2231	3.1970
	mep(0.75)	234.02	15.304	5.9289	5.6123	5.3695	3.2496
	mep(0.9)	230.18	15.572	6.0783	5.7631	5.5047	3.2811
	mep(0.99)	226.25	16.097	6.3746	6.0086	5.7673	3.3176
	fep(0.5)	234.80	15.367	6.0292	5.7213	5.4755	3.2501
	fep(0.75)	215.02	15.921	6.4449	6.0982	5.8435	3.2944
	fep(0.9)	199.10	17.503	7.5125	7.0207	6.7294	3.4531
	fep(0.99)	164.88	22.047	10.309	9.5385	9.1512	3.7212

(g) $\mathrm{DS}_{\mathrm{num}}$ values.

Table S7: **Comparison of sampling quality for tRNAs.** Tables record specific values related to shapes of predictions and sampled structures, obtained from our tRNA database. All results were computed by 10-fold cross-validation procedures, using sample size 1000 and $\min_{\mathrm{hel}} = \min_{HL} = 1$.

Approach	Errors	Shape Level					
		0	1	2	3	4	5
SCFG	—	0.0000	0.0026	0.0052	0.0131	0.0366	0.7110
	mep(0.5)	0.0000	0.0009	0.0026	0.0113	0.0287	0.7128
	mep(0.75)	0.0000	0.0017	0.0035	0.0105	0.0322	0.7050
	mep(0.9)	0.0000	0.0009	0.0017	0.0078	0.0331	0.7180
	mep(0.99)	0.0000	0.0026	0.0044	0.0095	0.0227	0.6919
	fep(0.5)	0.0000	0.0017	0.0043	0.0113	0.0374	0.6954
	fep(0.75)	0.0000	0.0000	0.0009	0.0113	0.0321	0.6710
	fep(0.9)	0.0000	0.0009	0.0009	0.0052	0.0261	0.6536
	fep(0.99)	0.0000	0.0000	0.0000	0.0017	0.0096	0.5474
LSCFG	—	0.2141	0.4256	0.4744	0.4900	0.9408	0.9843
	mep(0.5)	0.2141	0.4256	0.4744	0.4900	0.9408	0.9843
	mep(0.75)	0.2141	0.4248	0.4726	0.4892	0.9399	0.9843
	mep(0.9)	0.2089	0.4274	0.4761	0.4926	0.9399	0.9843
	mep(0.99)	0.1941	0.4221	0.4761	0.4892	0.9452	0.9852
	fep(0.5)	0.2124	0.4248	0.4726	0.4883	0.9417	0.9852
	fep(0.75)	0.1898	0.4213	0.4674	0.4831	0.9408	0.9843
	fep(0.9)	0.1314	0.4013	0.4518	0.4726	0.9321	0.9869
	fep(0.99)	0.0209	0.3029	0.3725	0.4186	0.8529	0.9809

(a) CSP_{freq} values (for selection principle MP struct.).

Approach	Errors	Shape Level					
		0	1	2	3	4	5
SCFG	—	0.0000	0.0026	0.0052	0.0131	0.0357	0.7128
	mep(0.5)	0.0000	0.0009	0.0026	0.0122	0.0305	0.7180
	mep(0.75)	0.0000	0.0017	0.0043	0.0113	0.0331	0.7067
	mep(0.9)	0.0000	0.0009	0.0017	0.0078	0.0357	0.7215
	mep(0.99)	0.0000	0.0026	0.0044	0.0105	0.0235	0.6902
	fep(0.5)	0.0000	0.0017	0.0043	0.0113	0.0383	0.6971
	fep(0.75)	0.0000	0.0000	0.0009	0.0113	0.0296	0.6745
	fep(0.9)	0.0000	0.0000	0.0000	0.0035	0.0261	0.6631
	fep(0.99)	0.0000	0.0000	0.0000	0.0035	0.0200	0.5439
LSCFG	—	0.2002	0.4256	0.4700	0.4866	0.9417	0.9861
	mep(0.5)	0.1332	0.3960	0.4439	0.4587	0.9434	0.9869
	mep(0.75)	0.0923	0.3847	0.4448	0.4639	0.9356	0.9861
	mep(0.9)	0.0575	0.3508	0.4135	0.4352	0.9373	0.9887
	mep(0.99)	0.0365	0.3630	0.4308	0.4491	0.9304	0.9861
	fep(0.5)	0.0801	0.3847	0.4404	0.4561	0.9400	0.9861
	fep(0.75)	0.0339	0.3630	0.4230	0.4430	0.9208	0.9843
	fep(0.9)	0.0131	0.3160	0.3743	0.4204	0.8442	0.9843
	fep(0.99)	0.0035	0.1497	0.2106	0.3325	0.5440	0.9730

(b) CSP_{freq} values (for selection principle MF struct.).

Approach	Errors	Shape Level					
		0	1	2	3	4	5
SCFG	—	0.0000	0.0000	0.0000	0.0000	0.0261	0.3821
	mep(0.5)	0.0000	0.0000	0.0000	0.0000	0.0209	0.3698
	mep(0.75)	0.0000	0.0000	0.0000	0.0000	0.0209	0.3559
	mep(0.9)	0.0000	0.0000	0.0000	0.0000	0.0131	0.3290
	mep(0.99)	0.0000	0.0000	0.0000	0.0000	0.0122	0.3003
	fep(0.5)	0.0000	0.0000	0.0000	0.0000	0.0252	0.3438
	fep(0.75)	0.0000	0.0000	0.0000	0.0000	0.0139	0.2463
	fep(0.9)	0.0000	0.0000	0.0000	0.0000	0.0070	0.1619
	fep(0.99)	0.0000	0.0000	0.0000	0.0000	0.0026	0.0444
LSCFG	—	0.1062	0.3891	0.4291	0.4378	0.9051	0.9835
	mep(0.5)	0.1010	0.3751	0.4134	0.4239	0.8921	0.9782
	mep(0.75)	0.0749	0.3647	0.4047	0.4282	0.8894	0.9774
	mep(0.9)	0.0470	0.3290	0.3769	0.3917	0.8834	0.9817
	mep(0.99)	0.0392	0.3429	0.3986	0.4213	0.8712	0.9791
	fep(0.5)	0.0740	0.3839	0.4239	0.4387	0.8877	0.9791
	fep(0.75)	0.0287	0.3516	0.3943	0.4134	0.8616	0.9713
	fep(0.9)	0.0139	0.2968	0.3490	0.3855	0.8120	0.9739
	fep(0.99)	0.0017	0.1358	0.1863	0.2942	0.4970	0.9634

(c) $\mathrm{CSP}_{\mathrm{freq}}$ values (for selection principle MEA struct.).

Approach	Errors	Shape Level					
		0	1	2	3	4	5
SCFG	—	0.0000	0.0000	0.0000	0.0000	0.0104	0.1097
	mep(0.5)	0.0000	0.0000	0.0000	0.0000	0.0104	0.1062
	mep(0.75)	0.0000	0.0000	0.0000	0.0000	0.0078	0.0923
	mep(0.9)	0.0000	0.0000	0.0000	0.0000	0.0044	0.0896
	mep(0.99)	0.0000	0.0000	0.0000	0.0000	0.0078	0.0827
	fep(0.5)	0.0000	0.0000	0.0000	0.0000	0.0061	0.0932
	fep(0.75)	0.0000	0.0000	0.0000	0.0000	0.0026	0.0696
	fep(0.9)	0.0000	0.0000	0.0000	0.0000	0.0017	0.0479
	fep(0.99)	0.0000	0.0000	0.0000	0.0000	0.0009	0.0078
LSCFG	—	0.0966	0.2916	0.3238	0.3316	0.8703	0.9686
	mep(0.5)	0.0879	0.3142	0.3516	0.3621	0.8625	0.9686
	mep(0.75)	0.0644	0.3029	0.3403	0.3551	0.8407	0.9678
	mep(0.9)	0.0427	0.2829	0.3194	0.3299	0.8451	0.9678
	mep(0.99)	0.0322	0.2924	0.3377	0.3595	0.8294	0.9651
	fep(0.5)	0.0662	0.3194	0.3551	0.3638	0.8512	0.9695
	fep(0.75)	0.0261	0.2907	0.3255	0.3516	0.8103	0.9608
	fep(0.9)	0.0113	0.2411	0.2872	0.3194	0.7650	0.9565
	fep(0.99)	0.0017	0.1053	0.1471	0.2219	0.4831	0.9339

(d) $\mathrm{CSP}_{\mathrm{freq}}$ values (for selection principle Centroid).

Approach	Errors	Shape Level					
		0	1	2	3	4	5
SCFG	—	0.0000	0.2855	0.4526	0.9852	0.9974	1.0000
	mep(0.5)	0.0000	0.2750	0.4256	0.9835	0.9991	1.0000
	mep(0.75)	0.0000	0.2367	0.3768	0.9774	0.9982	1.0000
	mep(0.9)	0.0000	0.2185	0.3394	0.9696	0.9965	1.0000
	mep(0.99)	0.0000	0.1715	0.2977	0.9756	0.9991	1.0000
	fep(0.5)	0.0000	0.2237	0.3543	0.9739	0.9991	1.0000
	fep(0.75)	0.0000	0.1584	0.2472	0.9574	0.9957	1.0000
	fep(0.9)	0.0000	0.0749	0.1497	0.9147	0.9930	1.0000
	fep(0.99)	0.0000	0.0174	0.0296	0.6763	0.9608	1.0000
LSCFG	—	0.6258	0.8912	0.9295	0.9504	0.9948	1.0000
	mep(0.5)	0.6084	0.8947	0.9286	0.9469	0.9948	1.0000
	mep(0.75)	0.5727	0.8886	0.9269	0.9521	0.9957	1.0000
	mep(0.9)	0.5231	0.8851	0.9252	0.9521	0.9948	1.0000
	mep(0.99)	0.4630	0.8894	0.9199	0.9452	0.9939	1.0000
	fep(0.5)	0.5553	0.8868	0.9234	0.9504	0.9948	1.0000
	fep(0.75)	0.4248	0.8894	0.9225	0.9521	0.9948	1.0000
	fep(0.9)	0.2393	0.8720	0.9077	0.9504	0.9957	1.0000
	fep(0.99)	0.0279	0.7580	0.8460	0.9617	0.9939	1.0000

(e) CSO_{freq} values.

Approach	Errors	Shape Level					
		0	1	2	3	4	5
SCFG	—	0.0000	0.5432	1.1811	20.640	51.834	573.72
	mep(0.5)	0.0000	0.4980	1.0481	19.498	49.614	566.13
	mep(0.75)	0.0000	0.3977	0.8859	18.385	47.128	556.17
	mep(0.9)	0.0000	0.3655	0.7353	16.331	43.735	544.22
	mep(0.99)	0.0000	0.2768	0.5850	14.689	40.062	527.12
	fep(0.5)	0.0000	0.3865	0.7982	17.401	45.868	552.82
	fep(0.75)	0.0000	0.2481	0.4961	13.092	38.088	507.97
	fep(0.9)	0.0000	0.0957	0.2141	8.7270	27.742	443.65
	fep(0.99)	0.0000	0.0191	0.0348	2.9269	12.064	285.20
LSCFG	—	42.599	347.33	421.29	455.78	881.11	983.88
	mep(0.5)	38.324	346.45	419.23	455.04	875.92	983.26
	mep(0.75)	31.890	338.26	411.90	451.93	865.28	983.84
	mep(0.9)	23.180	316.85	389.48	434.11	853.47	984.36
	mep(0.99)	17.873	312.64	386.51	436.20	832.96	983.29
	fep(0.5)	29.194	342.28	413.57	454.07	861.46	983.23
	fep(0.75)	14.829	304.49	376.16	430.85	811.76	980.03
	fep(0.9)	7.7946	250.74	312.98	391.21	713.62	980.94
	fep(0.99)	0.9219	93.694	133.47	260.11	418.20	970.01

(f) CS_{num} values.

Approach	Errors	Shape Level					
		0	1	2	3	4	5
SCFG	—	999.67	941.77	866.98	336.69	167.10	16.476
	mep(0.5)	999.59	943.47	871.08	345.35	171.57	16.766
	mep(0.75)	999.61	946.99	878.58	358.32	179.50	17.508
	mep(0.9)	999.53	949.65	884.73	372.57	188.32	18.198
	mep(0.99)	999.49	953.90	894.21	393.84	201.28	18.948
	fep(0.5)	999.53	947.08	879.39	363.20	182.17	17.663
	fep(0.75)	999.39	955.12	898.94	414.68	213.39	20.622
	fep(0.9)	998.86	962.12	917.65	484.74	258.19	25.174
	fep(0.99)	996.37	966.76	933.73	632.35	367.71	40.976
LSCFG	—	318.99	24.878	19.283	8.2879	4.4246	1.2088
	mep(0.5)	320.15	25.352	19.707	8.3590	4.4759	1.2245
	mep(0.75)	318.55	26.391	20.555	8.5940	4.6395	1.2271
	mep(0.9)	320.83	27.964	21.834	8.8192	4.7788	1.2219
	mep(0.99)	326.13	30.176	23.552	9.1464	4.9729	1.2463
	fep(0.5)	321.32	26.848	21.000	8.6976	4.6169	1.2202
	fep(0.75)	324.45	31.466	24.610	9.4810	5.1226	1.2445
	fep(0.9)	336.16	41.060	32.527	11.069	6.0148	1.2880
	fep(0.99)	401.88	84.057	69.689	18.023	9.1200	1.3690

(g) DS_{num} values.

Table S8: **Comparison of sampling quality for 5S rRNAs.** Tables record specific values related to shapes of predictions and sampled structures, obtained from our 5S rRNA database. All results were computed by 10-fold cross-validation procedures, using sample size 1000 and $\min_{hel} = \min_{HL} = 1$.

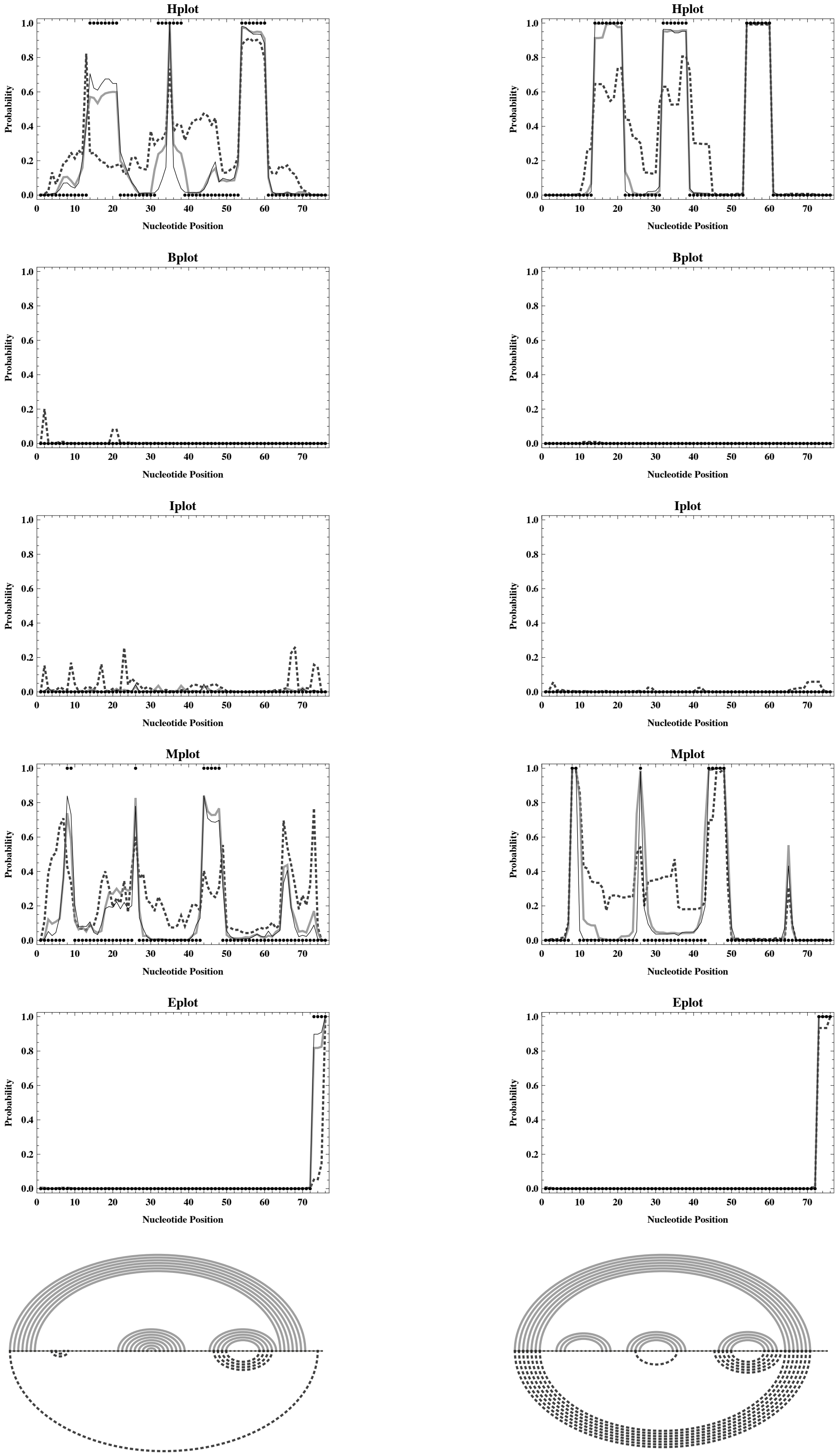

Figure S1: Loop profiles and centroid for *E.coli* tRNA^{Ala} derived according to mep($prob$) (thick gray lines) and fep($prob$) (thick dotted darker gray lines) under the assumption of the SCFG (figures on the left) and LSCFG (figures on the right) model, respectively, where percentage $prob = 0.99$ has been used for generating the relative errors. Hplot, Bplot, Iplot, Mplot and Eplot display the probability that an unpaired base lies in a hairpin, bulge, interior, multi-branched and exterior loop, respectively.

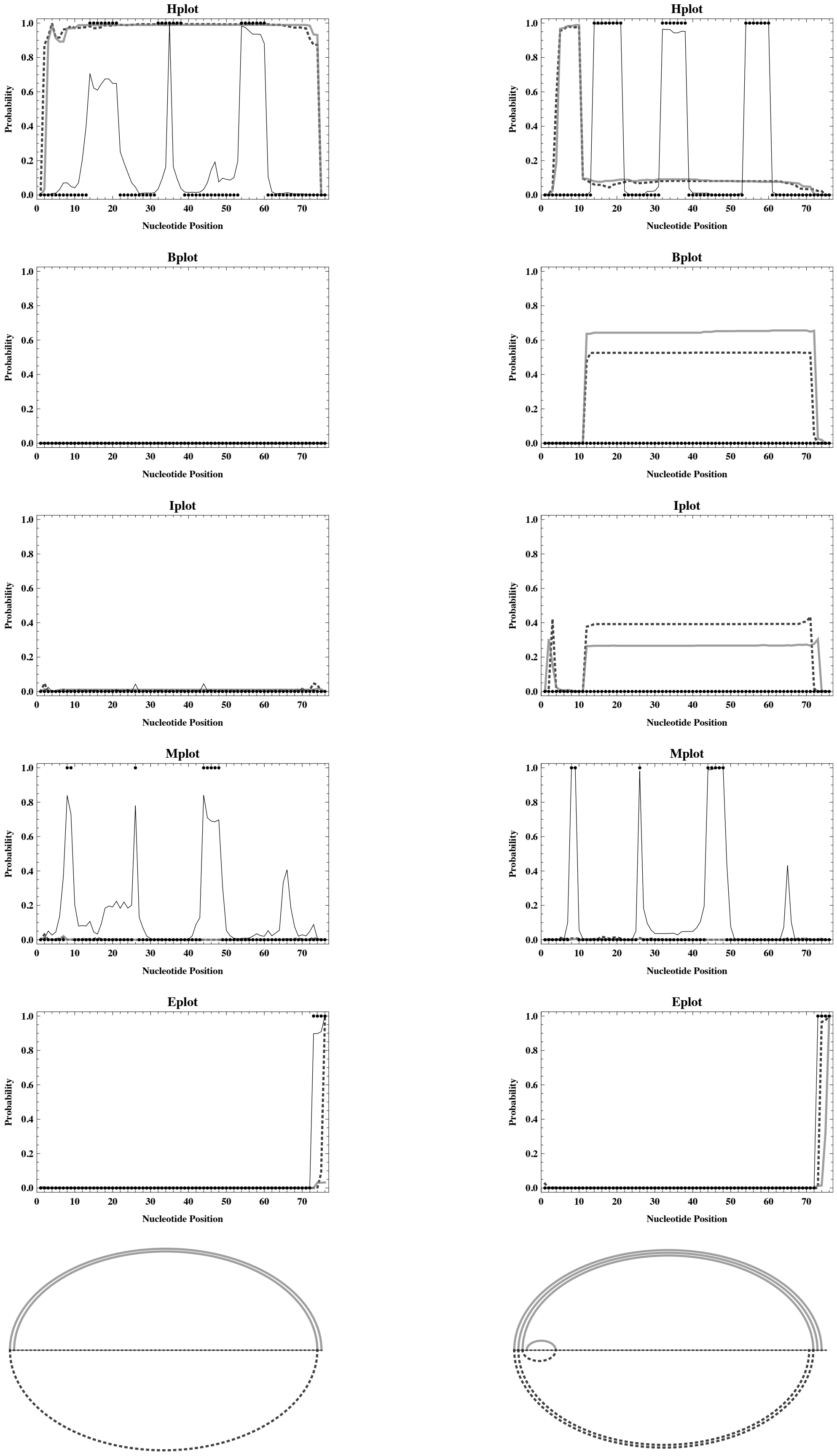

Figure S2: Loop profiles and centroid for *E.coli* tRNA^{Ala} derived according to mev($prob$) (thick gray lines) and fev($prob$) (thick dotted darker gray lines) under the assumption of the SCFG (figures on the left) and LSCFG (figures on the right) model, respectively, where fixed value $prob = 10^{-9}$ has been used for generating the absolute errors. Hplot, Bplot, Iplot, Mplot and Eplot display the probability that an unpaired base lies in a hairpin, bulge, interior, multi-branched and exterior loop, respectively.

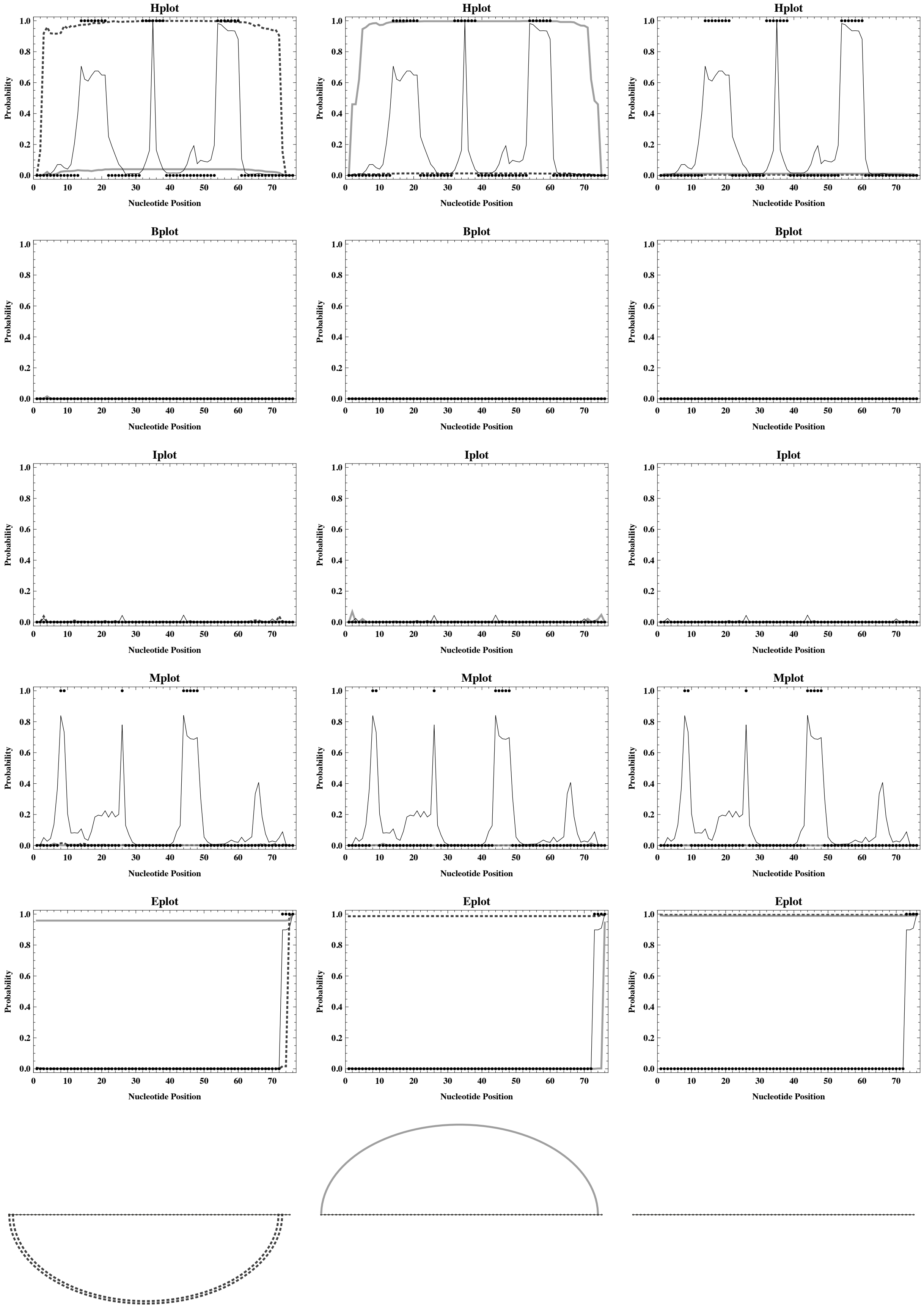

Figure S3: Loop profiles and centroid derived according to $\mathrm{mev}^{win,+}(prob)$ (thick gray lines) and $\mathrm{fev}^{win,+}(prob)$ (thick dotted darker gray lines) for the traditional SCFG model, respectively, where $prob = 10^{-9}$ and $win \in \{15, 38, 60\}$ (figures from left to right).

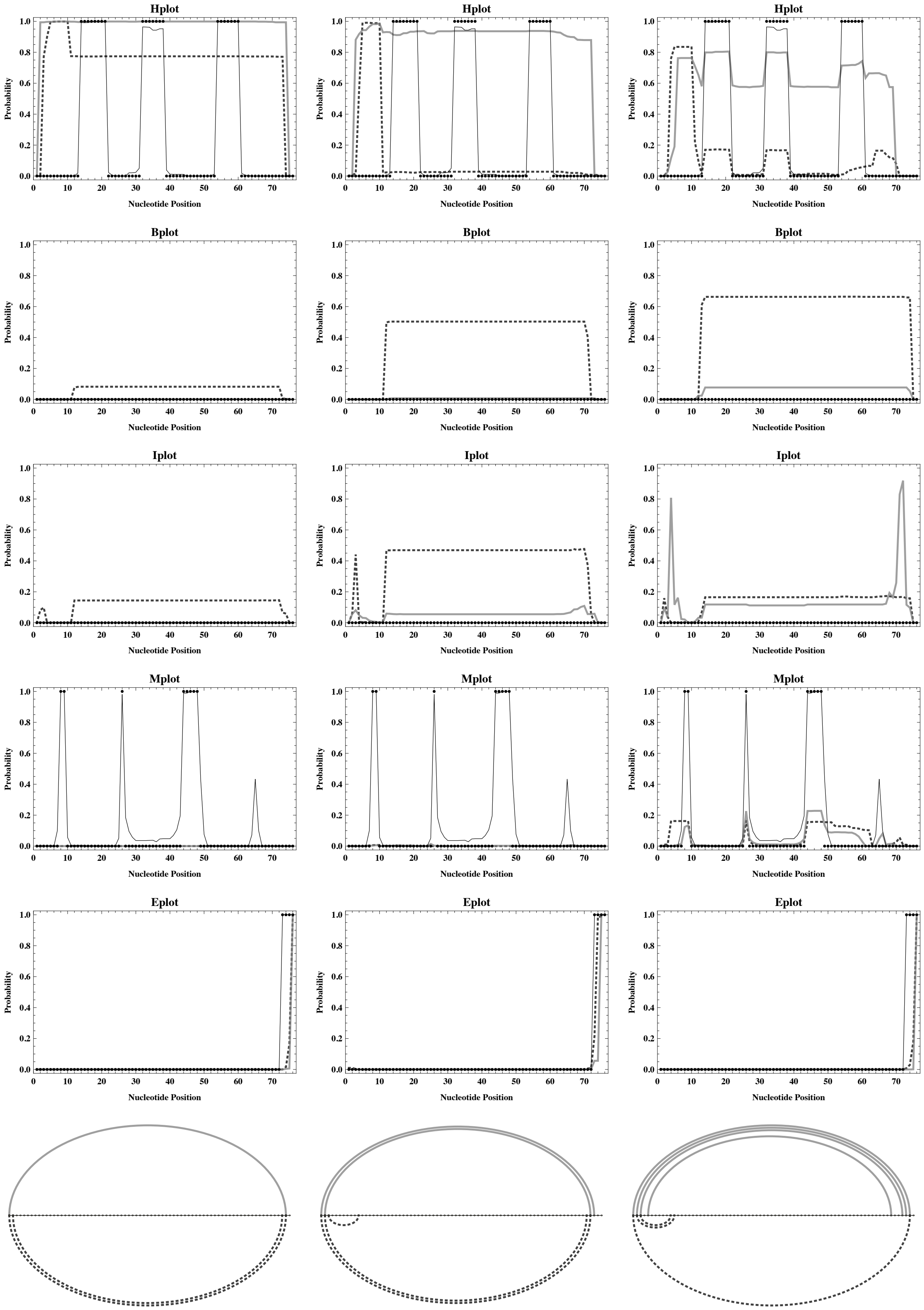

Figure S4: Loop profiles and centroid derived according to $\text{mev}^{win,+}(prob)$ (thick gray lines) and $\text{fev}^{win,+}(prob)$ (thick dotted darker gray lines) for the LSCFG model, respectively, where $prob = 10^{-9}$ and $win \in \{15, 38, 60\}$ (figures from left to right).

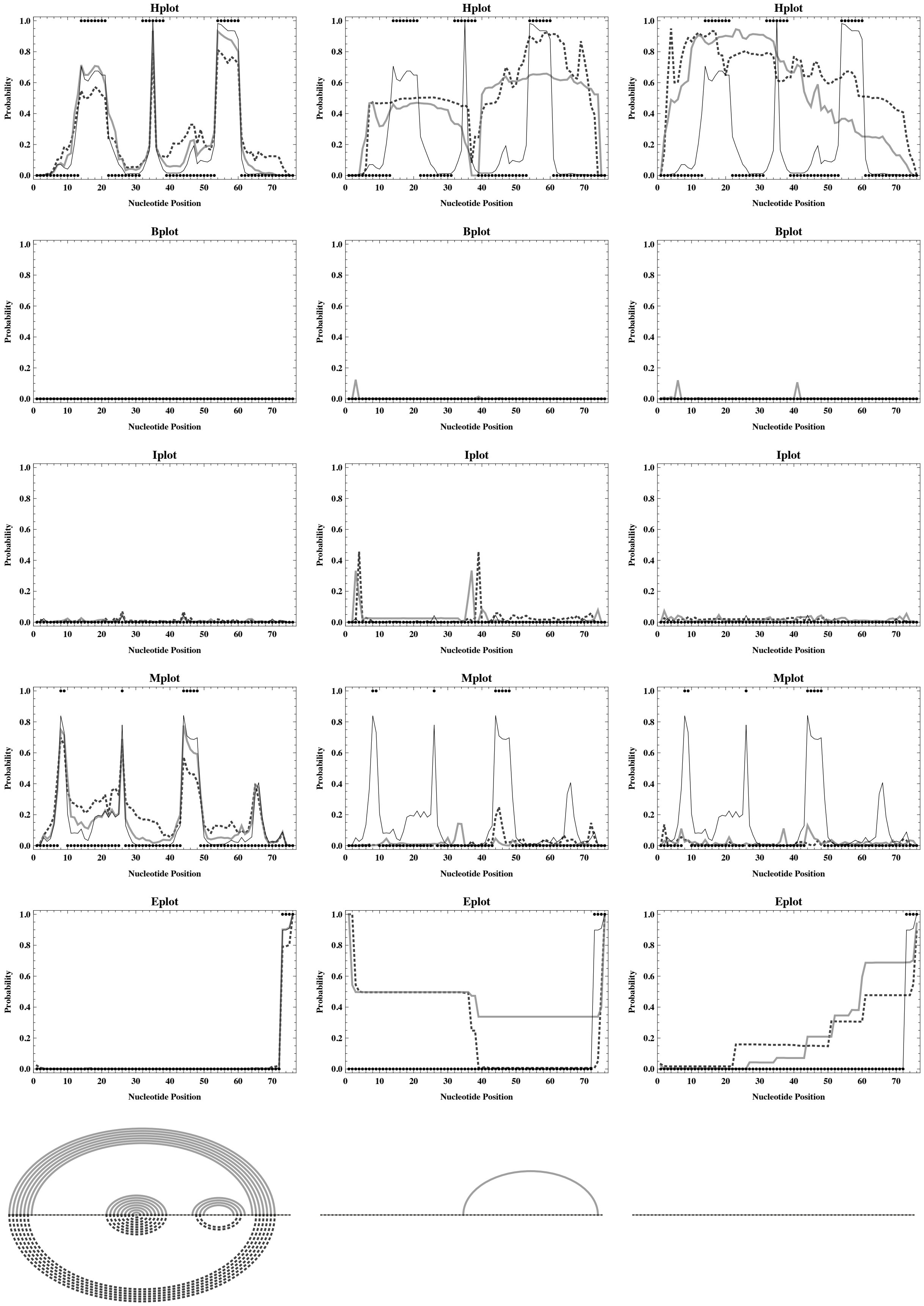

Figure S5: Loop profiles and centroid derived according to $\text{mev}^{win,-}(prob)$ (thick gray lines) and $\text{fev}^{win,-}(prob)$ (thick dotted darker gray lines) for the traditional SCFG model, respectively, where $prob = 10^{-9}$ and $win \in \{15, 38, 60\}$ (figures from left to right).

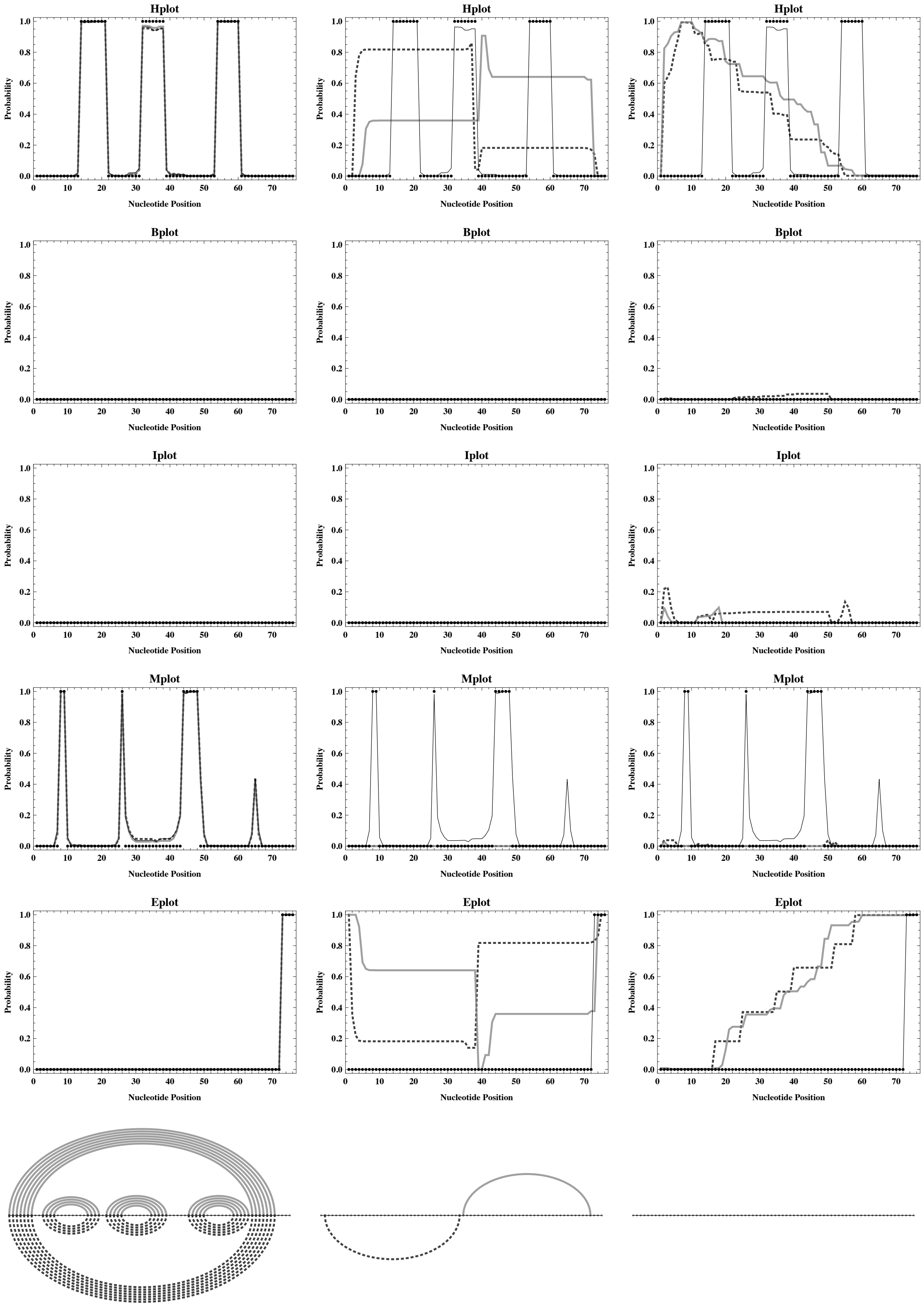

Figure S6: Loop profiles and centroid derived according to $\text{mev}^{win,-}(prob)$ (thick gray lines) and $\text{fev}^{win,-}(prob)$ (thick dotted darker gray lines) for the LSCFG model, respectively, where $prob = 10^{-9}$ and $win \in \{15, 38, 60\}$ (figures from left to right).

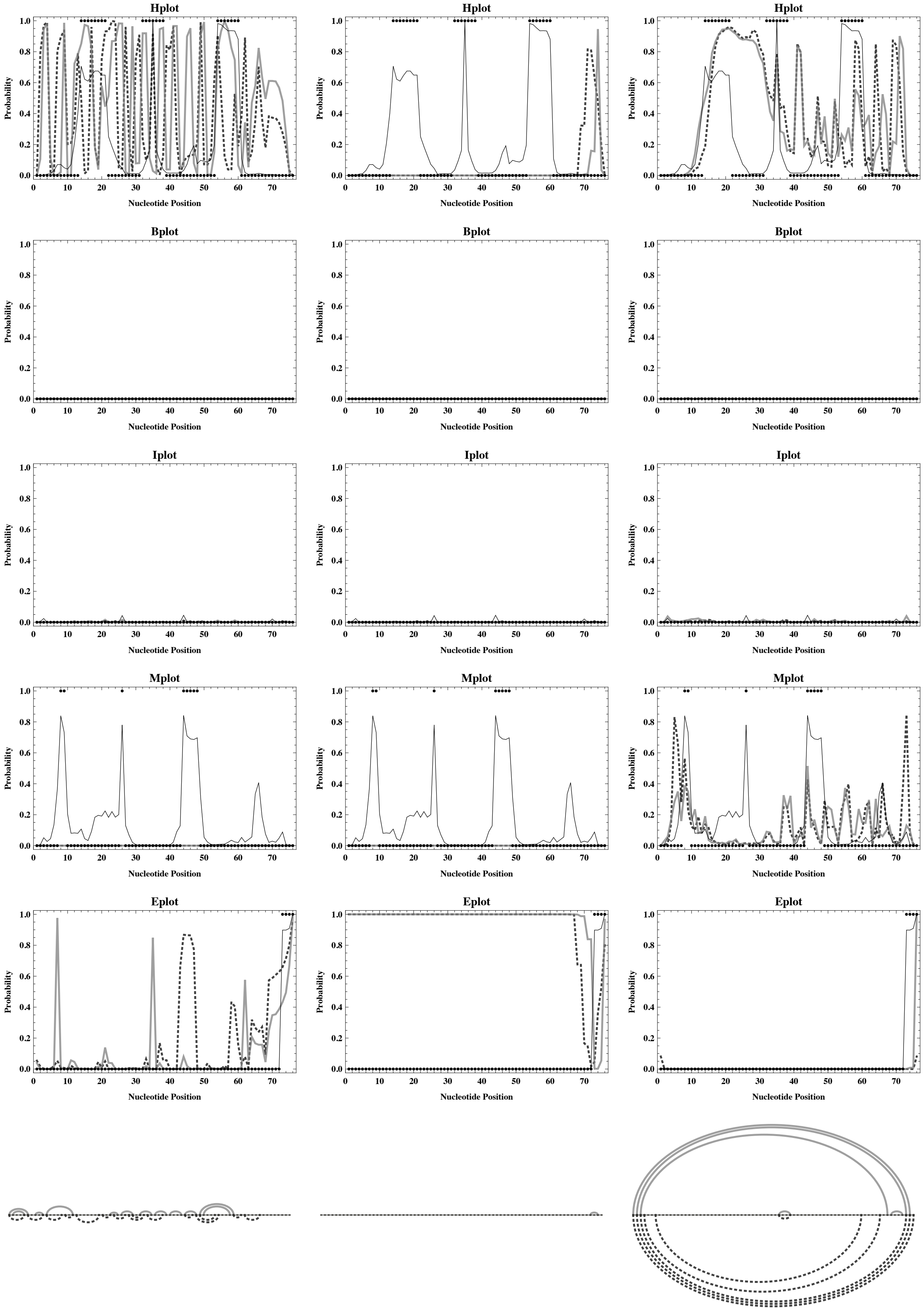

Figure S7: Loop profiles and centroid derived according to $\mathrm{mev}_{\mathcal{I}}(prob)$ (thick gray lines) and $\mathrm{fev}_{\mathcal{I}}(prob)$ (thick dotted darker gray lines) for the traditional SCFG model, respectively, where $prob = 10^{-9}$ and $\mathcal{I} \in \{\{T\}, \{C\}, \{A\}\}$ (figures from left to right).

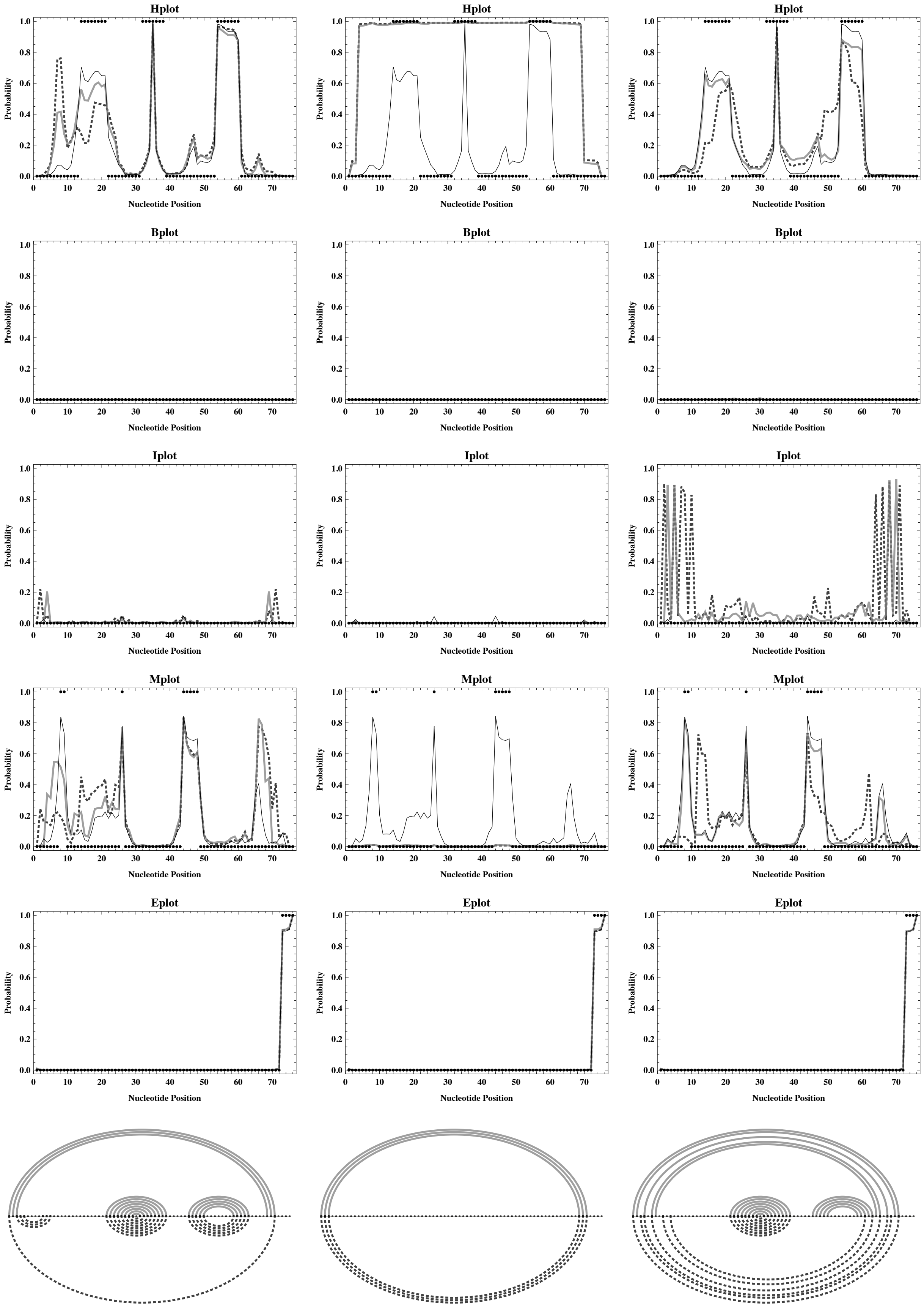

Figure S8: Loop profiles and centroid derived according to $\mathrm{mev}_{\mathcal{I}}(prob)$ (thick gray lines) and $\mathrm{fev}_{\mathcal{I}}(prob)$ (thick dotted darker gray lines) for the traditional SCFG model, respectively, where $prob = 10^{-9}$ and $\mathcal{I} \in \{\{P\}, \{F\}, \{G\}\}$ (figures from left to right).

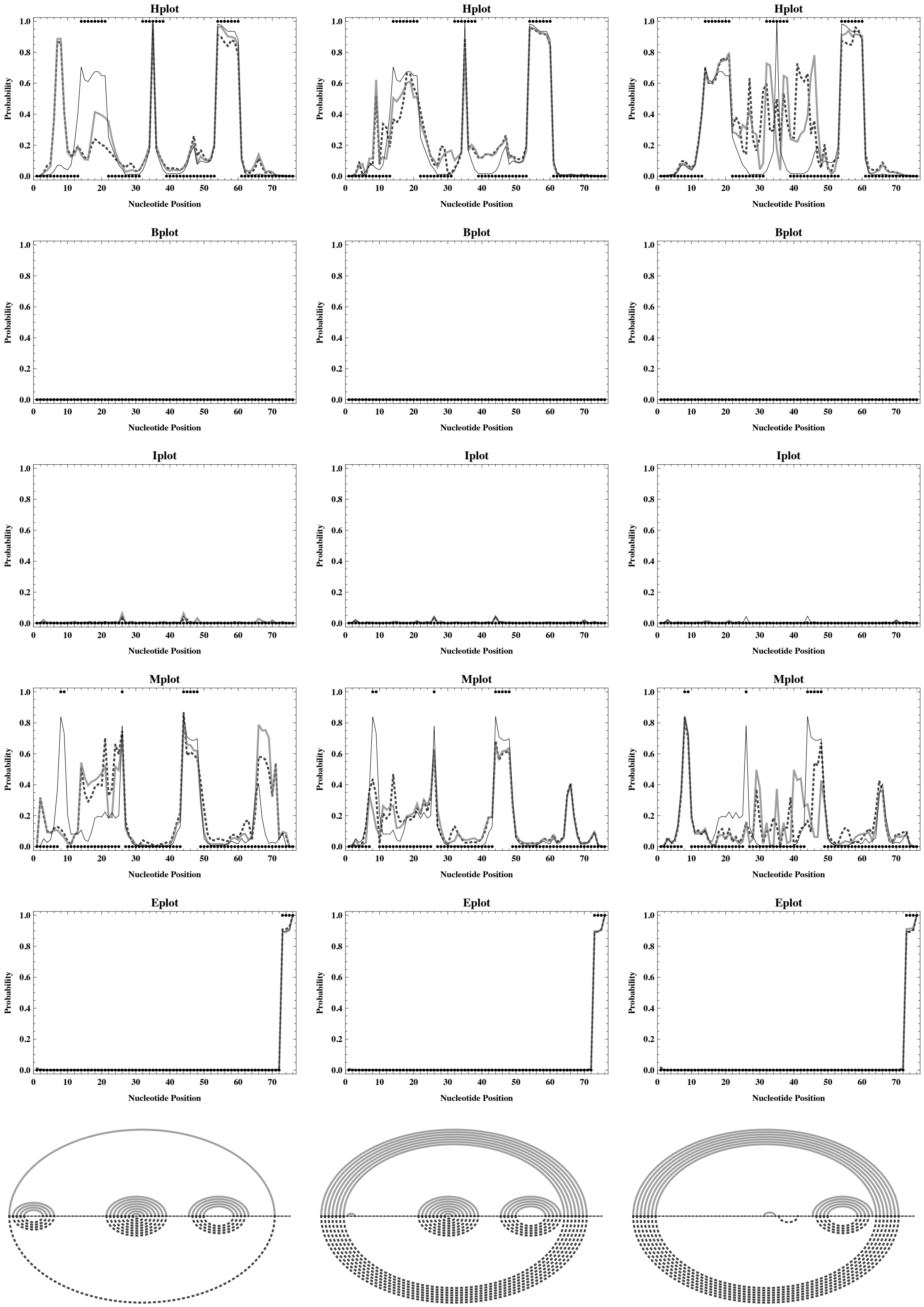

Figure S9: Loop profiles and centroid derived according to $\text{mev}_{\mathcal{I}}(prob)$ (thick gray lines) and $\text{fev}_{\mathcal{I}}(prob)$ (thick dotted darker gray lines) for the traditional SCFG model, respectively, where $prob = 10^{-9}$ and $\mathcal{I} \in \{\{M\}, \{O\}, \{N\}\}$ (figures from left to right).

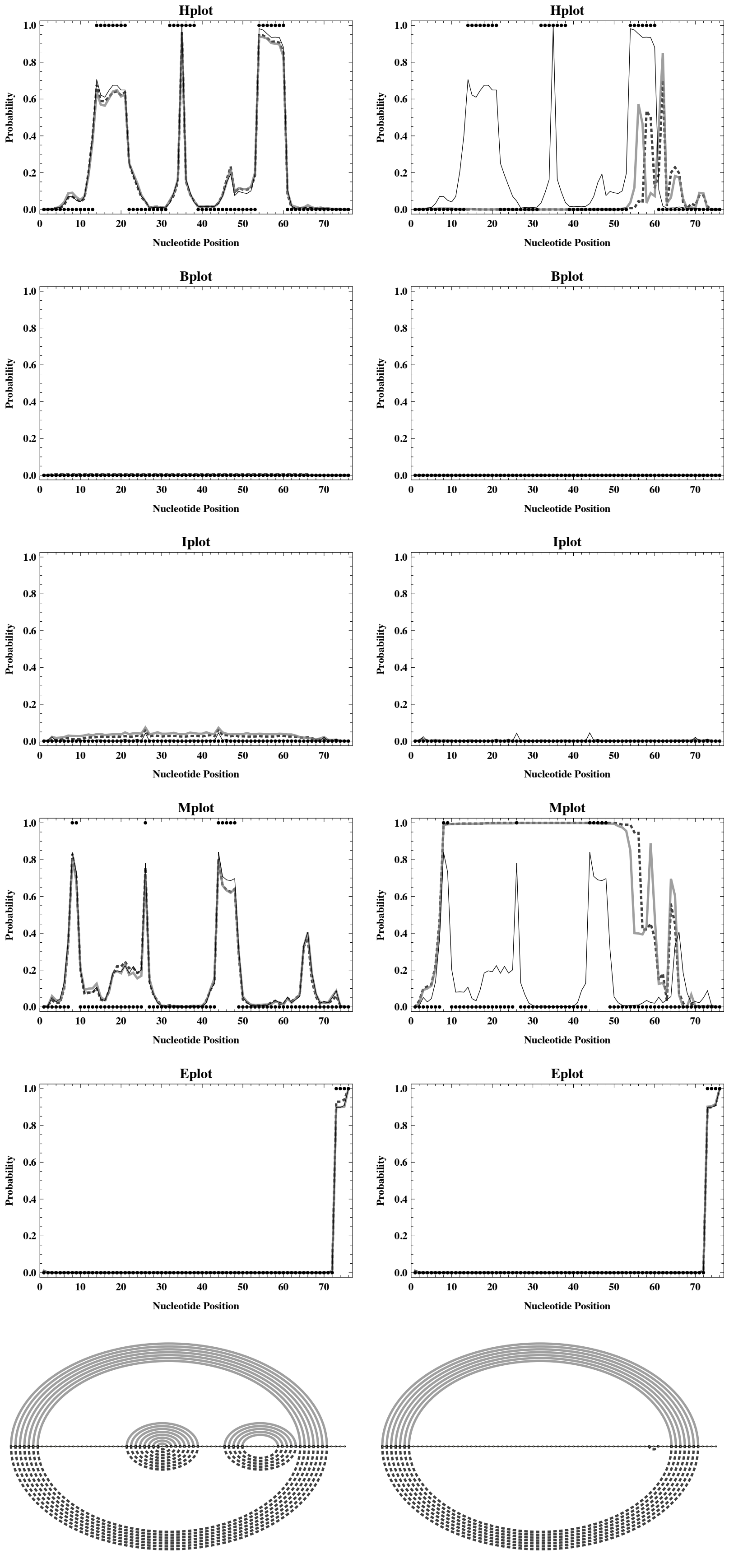

Figure S10: Loop profiles and centroid derived according to $\mathrm{mev}_{\mathcal{I}}(prob)$ (thick gray lines) and $\mathrm{fev}_{\mathcal{I}}(prob)$ (thick dotted darker gray lines) for the traditional SCFG model, respectively, where $prob = 10^{-9}$ and $\mathcal{I} \in \{\{B\}, \{U\}\}$ (figures from left to right).

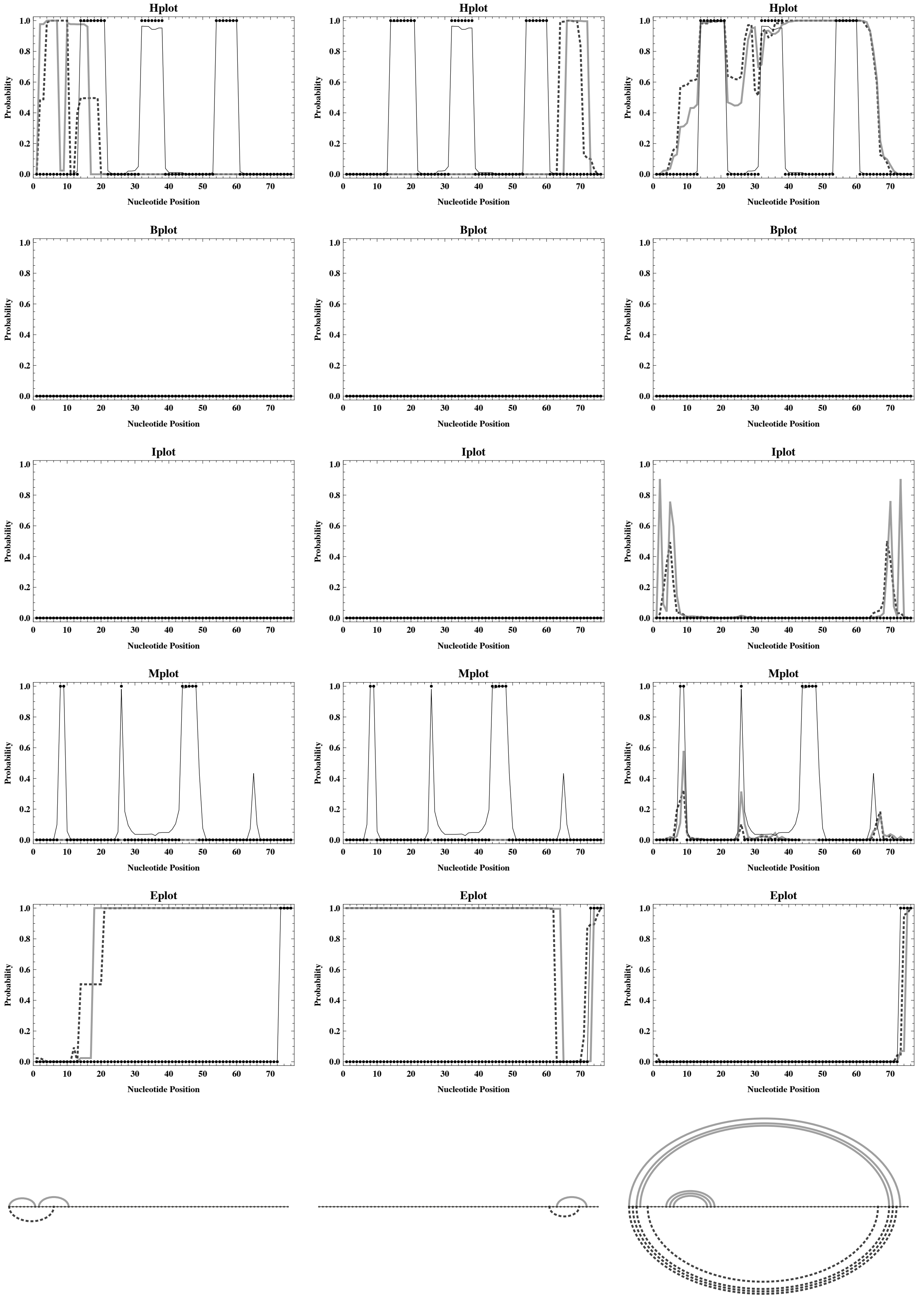

Figure S11: Loop profiles and centroid derived according to $\text{mev}_{\mathcal{I}}(prob)$ (thick gray lines) and $\text{fev}_{\mathcal{I}}(prob)$ (thick dotted darker gray lines) for the LSCFG model, respectively, where $prob = 10^{-9}$ and $\mathcal{I} \in \{\{T\}, \{C\}, \{A\}\}$ (figures from left to right).

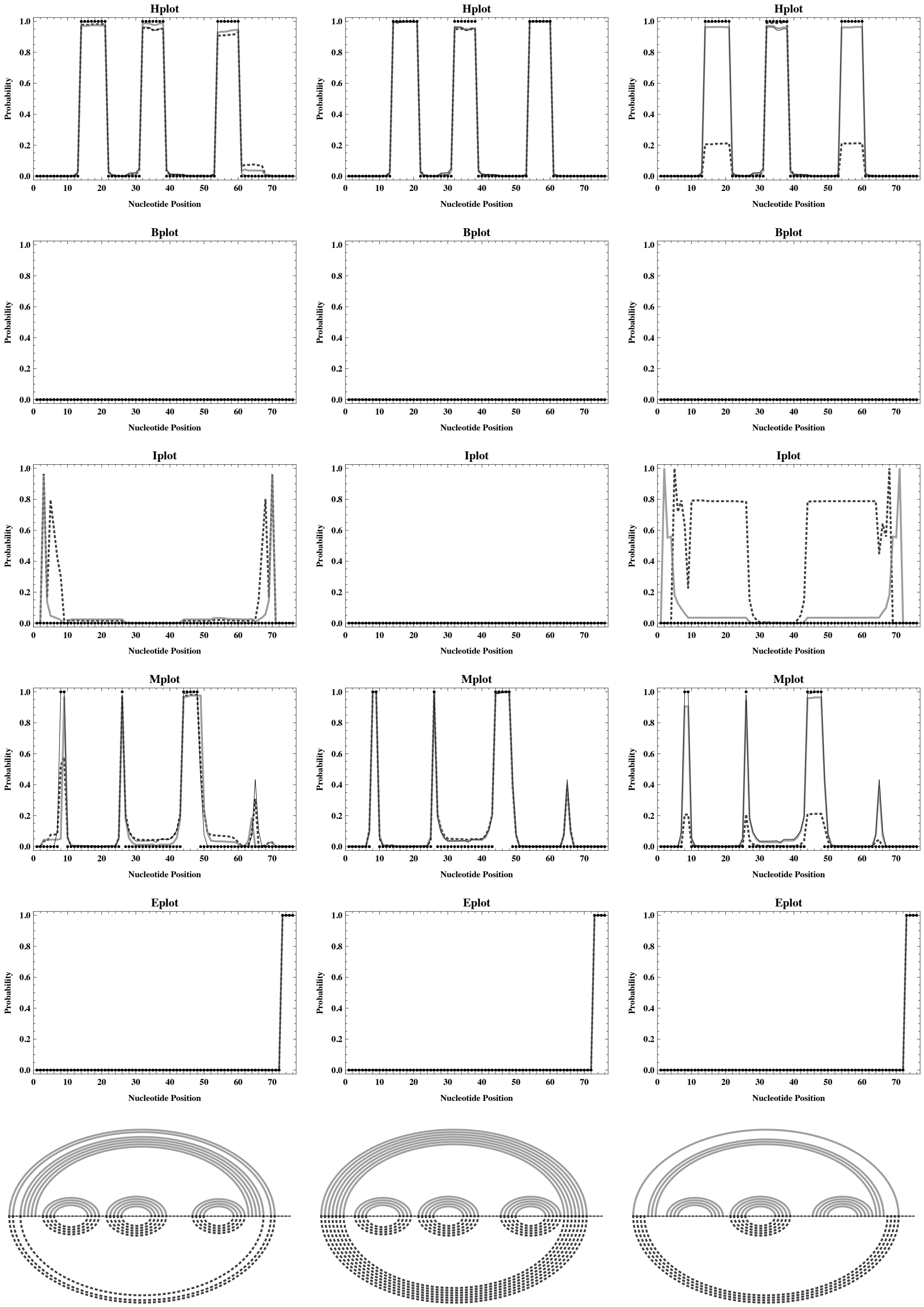

Figure S12: Loop profiles and centroid derived according to $\mathrm{mev}_{\mathcal{I}}(prob)$ (thick gray lines) and $\mathrm{fev}_{\mathcal{I}}(prob)$ (thick dotted darker gray lines) for the LSCFG model, respectively, where $prob = 10^{-9}$ and $\mathcal{I} \in \{\{P\}, \{F\}, \{G\}\}$ (figures from left to right).

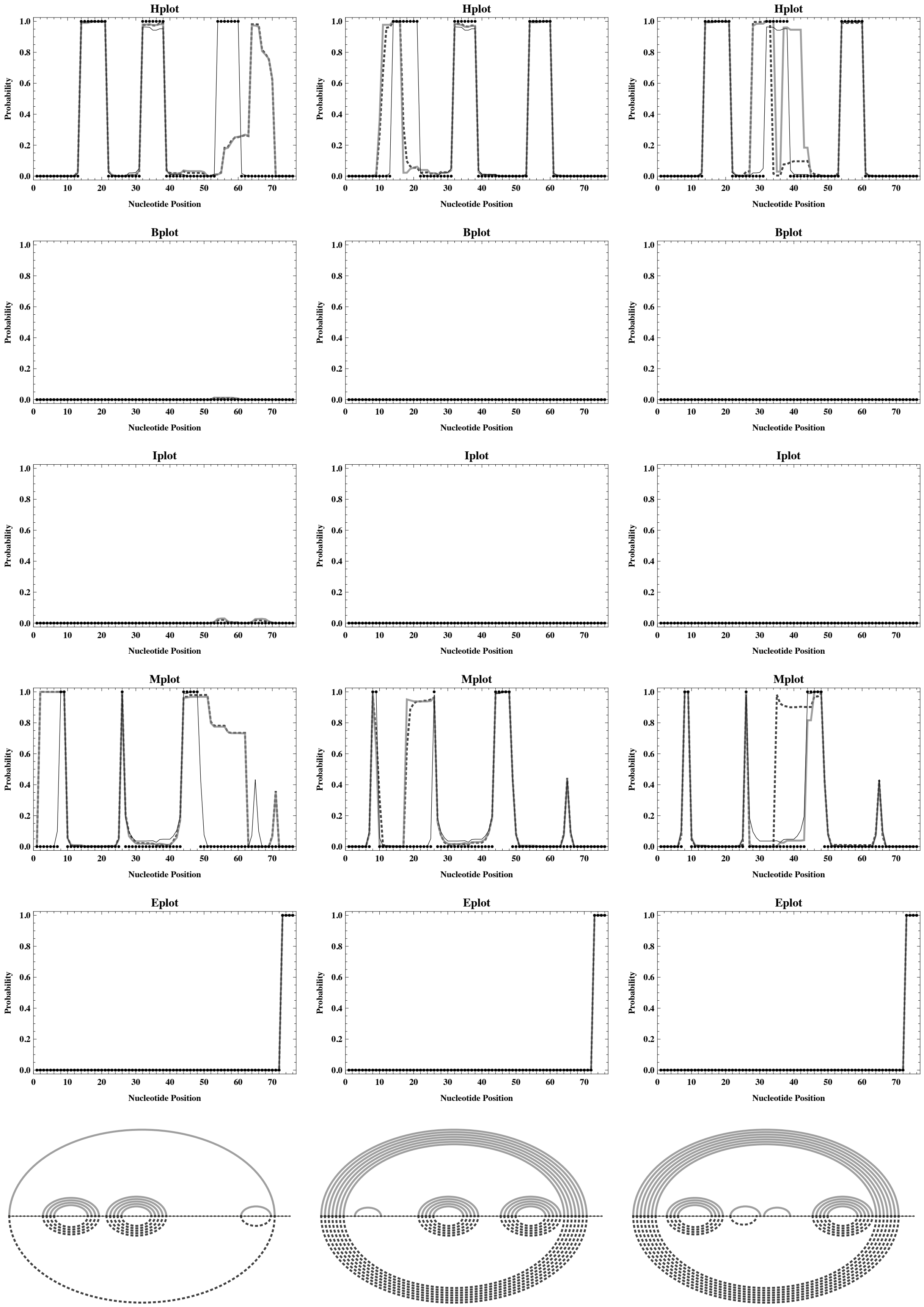

Figure S13: Loop profiles and centroid derived according to $\mathrm{mev}_{\mathcal{I}}(prob)$ (thick gray lines) and $\mathrm{fev}_{\mathcal{I}}(prob)$ (thick dotted darker gray lines) for the LSCFG model, respectively, where $prob = 10^{-9}$ and $\mathcal{I} \in \{\{M\}, \{O\}, \{N\}\}$ (figures from left to right).

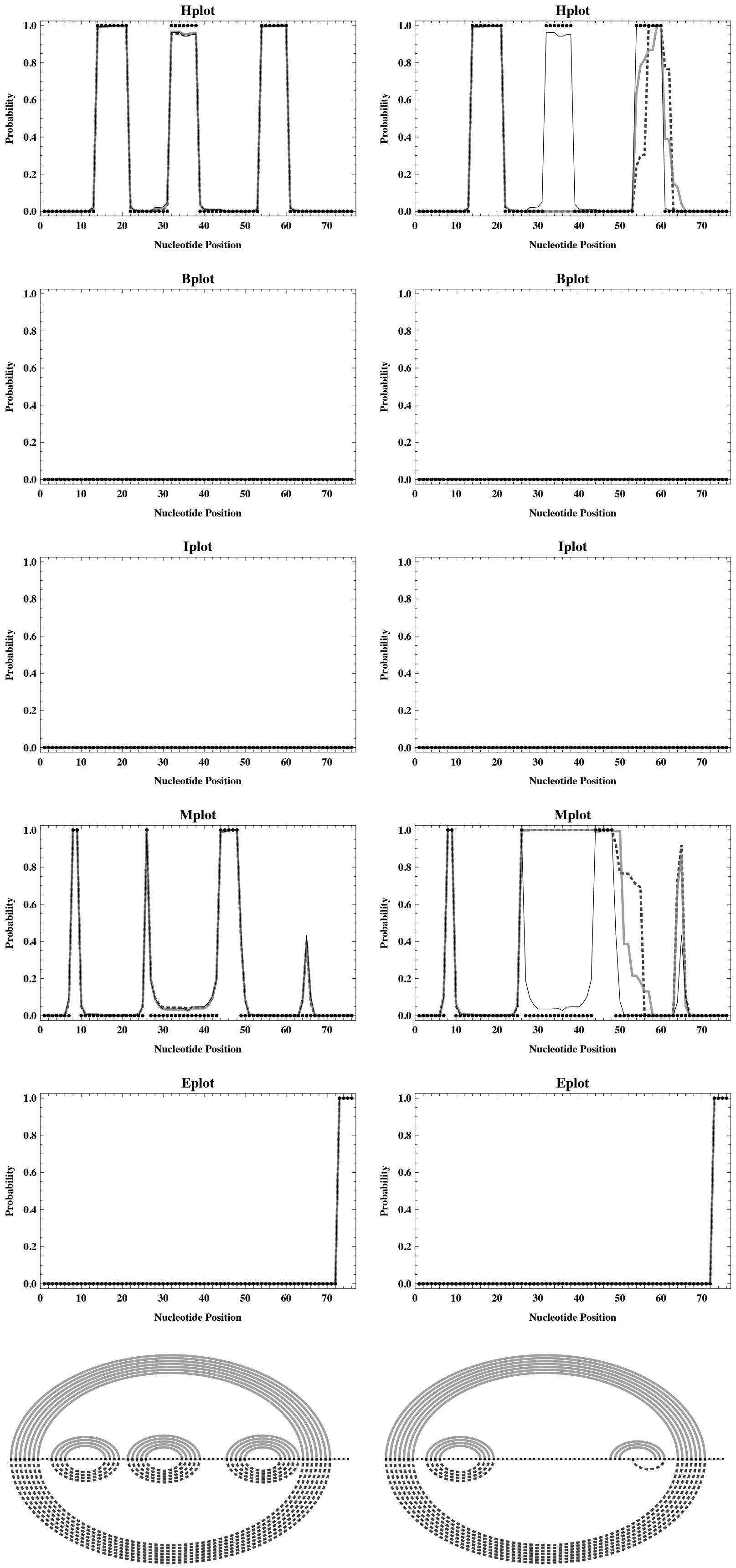

Figure S14: Loop profiles and centroid derived according to $\mathrm{mev}_{\mathcal{I}}(prob)$ (thick gray lines) and $\mathrm{fev}_{\mathcal{I}}(prob)$ (thick dotted darker gray lines) for the LSCFG model, respectively, where $prob = 10^{-9}$ and $\mathcal{I} \in \{\{B\},\{U\}\}$ (figures from left to right).

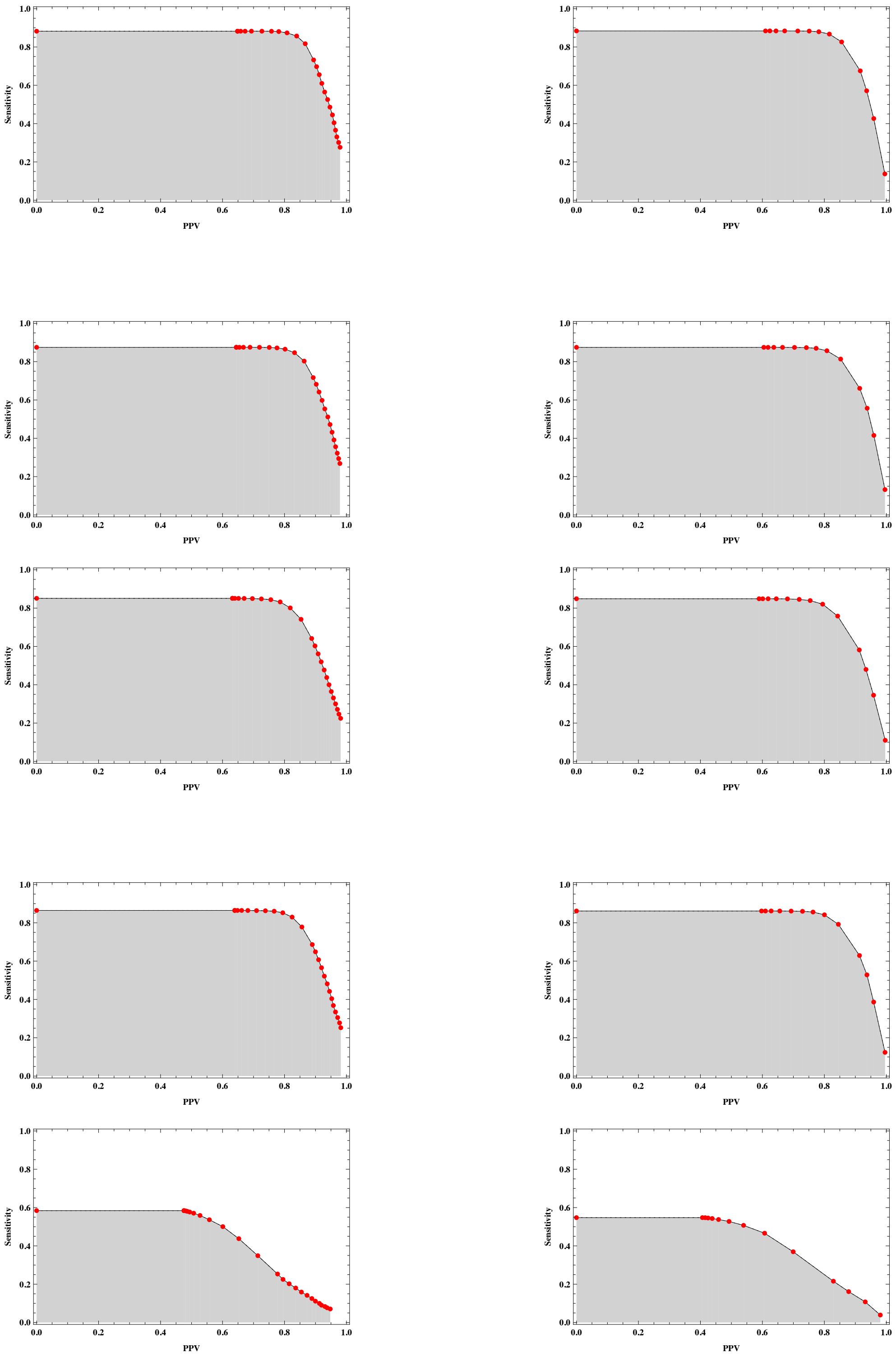

Figure S15: Comparison of the (areas under) ROC curves obtained for our tRNA database, derived without disturbances (top line) and by considering random relative disturbances according to mep(0.5), mep(0.99), fep(0.5) and fep(0.99) (from top to bottom line) under the assumption of the traditional SCFG model (for $\min_{\text{hel}} = 1$ and $\min_{HL} = 1$). For each preprocessing variant, corresponding ROC curves are shown for prediction principle MEA structure (figure on the left) and centroid (figure on the right), respectively.

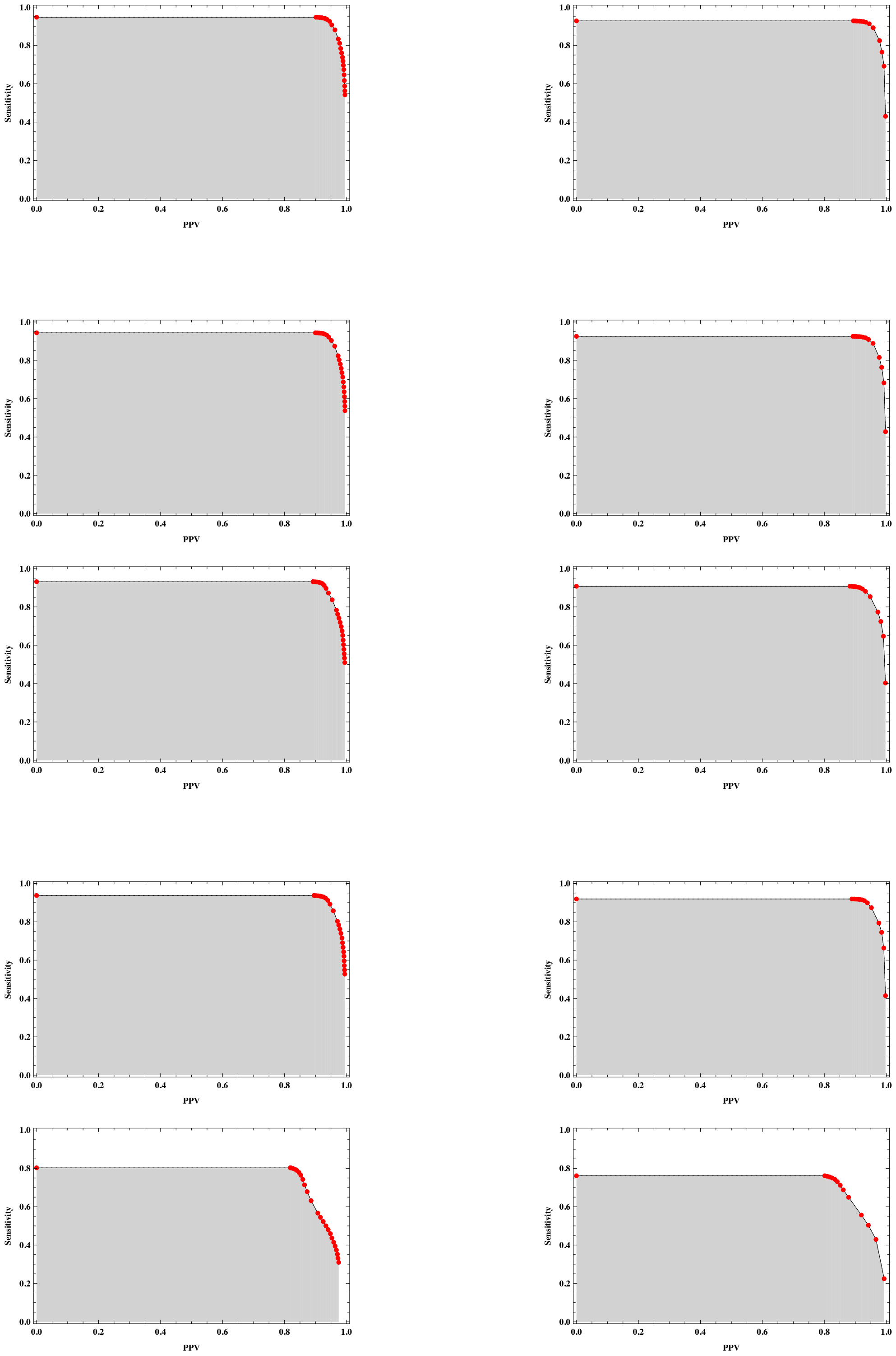

Figure S16: Results corresponding to those of Figure S15, derived under the assumption of the LSCFG model.

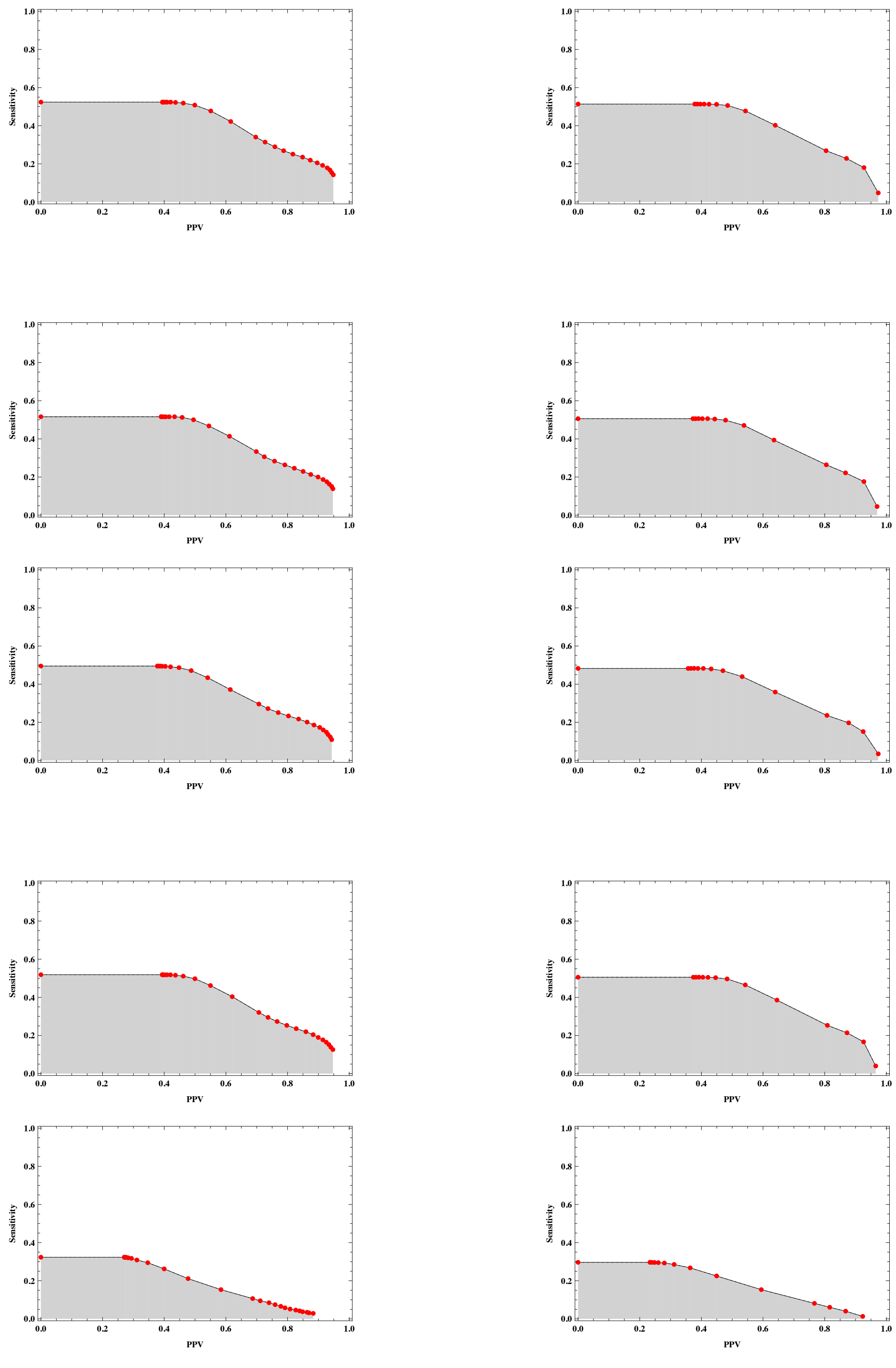

Figure S17: Comparison of the (areas under) ROC curves obtained for our 5S rRNA database, derived without disturbances (top line) and by considering random relative disturbances according to mep(0.5), mep(0.99), fep(0.5) and fep(0.99) (from top to bottom line) under the assumption of the traditional SCFG model (for $\min_{\text{hel}} = 1$ and $\min_{HL} = 1$). For each preprocessing variant, corresponding ROC curves are shown for prediction principle MEA structure (figure on the left) and centroid (figure on the right), respectively.

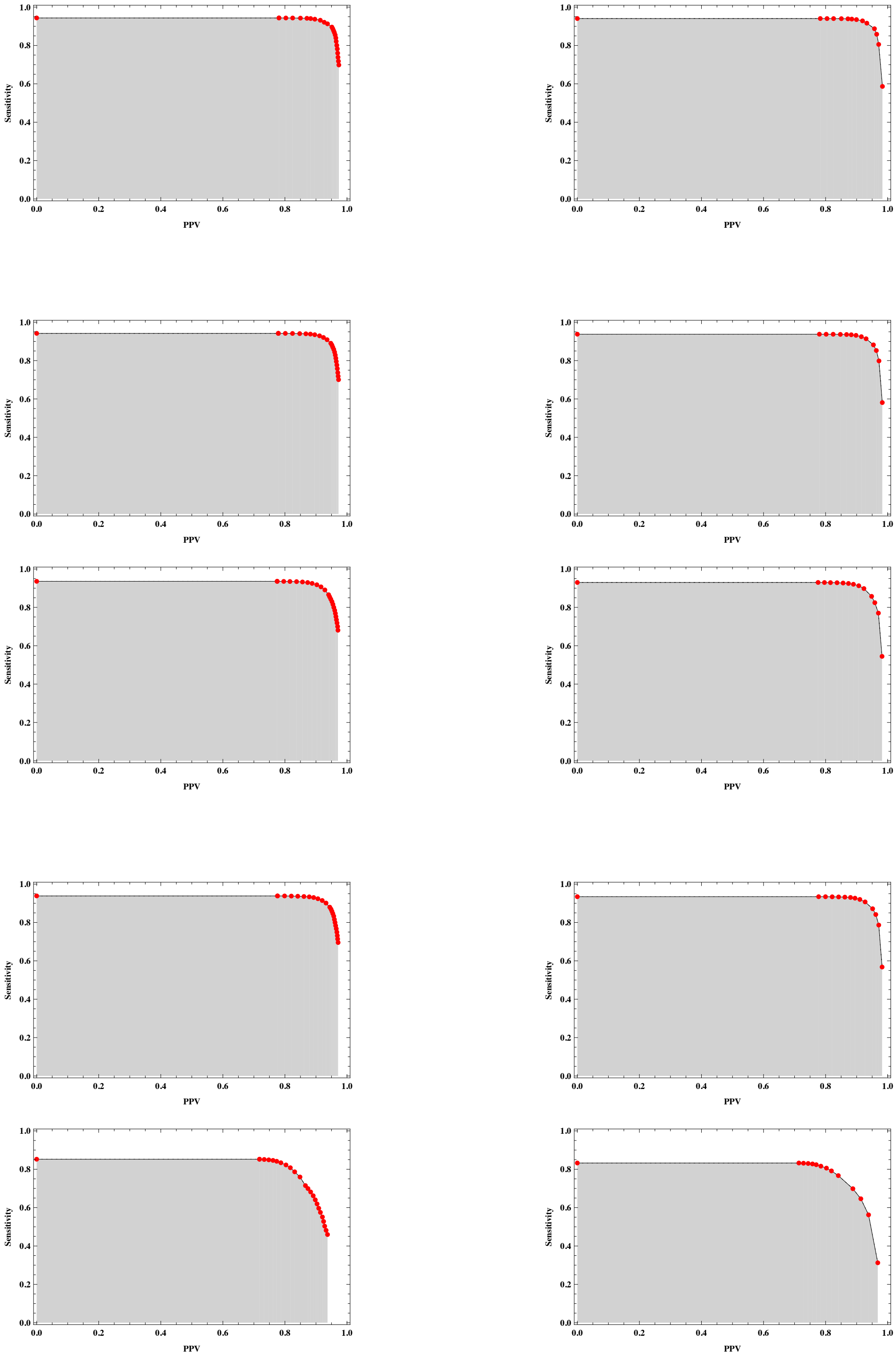

Figure S18: Results corresponding to those of Figure S17, derived under the assumption of the LSCFG model.
